# Targeting epigenetic features in clear cell sarcomas based on patient-derived cell lines

**DOI:** 10.1186/s12967-022-03843-4

**Published:** 2023-01-29

**Authors:** Christina Karner, Ines Anders, Djenana Vejzovic, Joanna Szkandera, Susanne Scheipl, Alexander J. A. Deutsch, Larissa Weiss, Klemens Vierlinger, Dagmar Kolb, Stefan Kühberger, Ellen Heitzer, Hansjörg Habisch, Fangrong Zhang, Tobias Madl, Birgit Reininger-Gutmann, Bernadette Liegl-Atzwanger, Beate Rinner

**Affiliations:** 1 Division of Biomedical Research, Core Facility Alternative Biomodels and Preclinical Imaging, Medical University of Graz, Roseggerweg 48, 8036 Graz, Austria; 2 Division of Oncology, Department of Internal Medicine, Medical University of Graz, Graz, Austria; 3 Department of Orthopedics and Trauma, Medical University of Graz, Graz, Austria; 4 Division of Hematology, Medical University of Graz, Graz, Austria; 5 Institute for Health Care Engineering With European Testing Center of Medical Devices, University of Technology, Graz, Austria; 6 Competence Unit Molecular Diagnostics, Center for Health and Bioresources, AIT Austrian Institute of Technology GmbH, Vienna, Austria; 7 Core Facility Ultrastructure Analysis, Center for Medical Research, Gottfried Schatz Research Center, Medical University of Graz, Graz, Austria; 8 Division of Cell Biology, Histology and Embryology, Gottfried Schatz Research Center, Medical University of Graz, Graz, Austria; 9 Diagnostic and Research Institute of Human Genetics, Diagnostic and Research Center for Molecular Biomedicine, Medical University of Graz, Graz, Austria; 10 Research Unit Integrative Structural Biology, Gottfried Schatz Research Center for Cell Signaling, Metabolism and Aging, Molecular Biology and Biochemistry, Medical University of Graz, Graz, Austria; 11 Key Laboratory of Ministry of Education of Gastrointestinal Cancer, Fujian Medical University, Fuzhou, China; 12 Diagnostic and Research Institute of Pathology, Medical University of Graz, Neue Stiftingtalstraße 6, 8010 Graz, Austria; 13 BioTechMed-Graz, Graz, Austria

**Keywords:** Clear cell sarcoma, Metastases, Epigenetic hall marks, Arginine methylation

## Abstract

**Background:**

Clear cell sarcomas (CCSs) are translocated aggressive malignancies, most commonly affecting young adults with a high incidence of metastases and a poor prognosis. Research into the disease is more feasible when adequate models are available. By establishing CCS cell lines from a primary and metastatic lesion and isolating healthy fibroblasts from the same patient, the in vivo process is accurately reflected and aspects of clinical multistep carcinogenesis recapitulated.

**Methods:**

Isolated tumor cells and normal healthy skin fibroblasts from the same patient were compared in terms of growth behavior and morphological characteristics using light and electron microscopy. Tumorigenicity potential was determined by soft agar colony formation assay and in vivo xenograft applications. While genetic differences between the two lineages were examined by copy number alternation profiles, nuclear magnetic resonance spectroscopy determined arginine methylation as epigenetic features. Potential anti-tumor effects of a protein arginine n-methyltransferase type I (PRMT1) inhibitor were elicited in 2D and 3D cell culture experiments using cell viability and apoptosis assays. Statistical significance was calculated by one-way ANOVA and unpaired t-test.

**Results:**

The two established CCS cell lines named MUG Lucifer prim and MUG Lucifer met showed differences in morphology, genetic and epigenetic data, reflecting the respective original tissue. The detailed cell line characterization especially in regards to the epigenetic domain allows investigation of new innovative therapies. Based on the epigenetic data, a PRMT1 inhibitor was used to demonstrate the targeted antitumor effect; normal tissue cells isolated and immortalized from the same patient were not affected with the IC_50_ used.

**Conclusions:**

MUG Lucifer prim, MUG Lucifer met and isolated and immortalized fibroblasts from the same patient represent an ideal in vitro model to explore the biology of CCS. Based on this cell culture model, novel therapies could be tested in the form of PRMT1 inhibitors, which drive tumor cells into apoptosis, but show no effect on fibroblasts, further supporting their potential as promising treatment options in the combat against CCS. The data substantiate the importance of tailored therapies in the advanced metastatic stage of CCS.

**Supplementary Information:**

The online version contains supplementary material available at 10.1186/s12967-022-03843-4.

## Background

Clear cell sarcomas (CCSs) are an extremely rare type of soft-tissue saromas arising from cells of the neural crest and occurring primarily in young adults. Patients with CCSs report a palpable tumor mass for months or years. Tumors larger than 5 cm, necrosis and lymph node invasion have an unfavorable outcome with a 5-year survival rate of 40–60% [1, 2]. CCSs are aggressive malignancies with a local recurrence rate of up to 40% and occurrence of pulmonary and lymph node metastases in up to 50% [3, 4]. However, metastatic spread is a complicated process and the molecular details of the cell’s metabolic transformation are still largely unknown despite its clinical importance [5].

CCSs are characterized by melanocytic differentiation including immunohistochemical positivity for S100 calcium-binding protein (S-100), SOX10, melan-A, and the melanoma-associated antigen human melanoma black 45 (HMB45) [6]. Histologically, CCSs harbor a distinctive nested or fascicular growth pattern with epithelioid to spindle-shaped cells separated by fibrous septa. Despite of its name, the cytoplasm of the tumor cells usually is pale, eosinophilic and the nuclei show prominent nucleoli. Only rarely true cytoplasmic clearing is seen in the vast majority of tumor cells. CCSs show characteristic fusions, predominantly the t(12;22)(q13;q12) translocation, leading to the *EWSR1::ATF1* gene fusion. In a smaller subset of cases a variant translocation t(2;22)(q34;q12) resulting in a *EWSR1::CREB1* fusion has been described [6].

Currently, the standard therapy remains wide surgical intervention. Novel therapeutic strategies as well as diagnostic and prognostic biomarkers are therefore urgently needed [7]. Several preclinical data suggest that epigenetic alterations including DNA methylation and histone acetylation may contribute to tumor pathogenesis through gene transcription alteration [8]. Thus, in bone and soft tissue tumors the observed alterations in the chromatin configuration and the associated modifications (such as methylation) appear to be related to tumorigenesis. A study in rhabdomyosarcoma cells demonstrated that the inhibition of the over-expressed protein arginine methyltransferases (PRMTs) using arginine methyltransferase inhibitor 1 (AMI-1) and S-adenosyl-I-homocysteine (SAH) led to reduced tumor growth and proliferation [9]. In another study in clear cell renal cell carcinoma, PRMT1 was shown to be suppressed by the novel PRMT1 inhibitor DCPT1061 both in vivo and in vitro. In addition, the combination of DCPT1061 and the first-line treatment with sunitinib improved the antiproliferative effect of the latter in vitro, and remission of tumor growth persisted even after sunitinib had been discontinued [10].

Arginine methylation (ArgMet) is a widespread post-translational modification that regulates a plethora of fundamental biological processes from transcription to RNA splicing and DNA damage signaling. Subsequently, it has emerged as the key regulator of the cell cycle in recent literature. In cells, three main types of methylated arginine residues are present including ω-N^G^ monomethylarginine (MMA), ω-N^G^-N^G^ asymmetric dimethylarginine (ADMA) and ω-N^G^-N’^G^ symmetric dimethylarginine (SDMA) [11, 12]. PRMTs catalyze the formation of MMA out of L-Arginine, and consecutively ADMA or SDMA. In detail, type I PRMTs including PRMT 1, 2, 3, 4 (also called CARM1), 6 and 8 catalyze MMA and ADMA, whereas type II PRMTs catalyze the formation of MMA and SDMA [13]. Small-molecule inhibitors of PRMTs represent a class of therapeutic agents affecting ArgMet levels and preventing protein ArgMet. Until recently, quantification of levels and dynamics of global ArgMet, and with this the impact of inhibitors on protein ArgMet, has been challenging due to the lack of suited methods. With the development of general methods for absolute, label-free quantification of (methylated) arginines in cells by using the high sensitivity and robustness of nuclear magnetic resonance (NMR) spectroscopy this bottleneck was overcome [14]. With these novel tools, the role of ArgMet and the impact of ArgMet inhibition on CCS treatment can now be studied.

The overall survival of CCS patients is still unsatisfactory, as the therapies used in clinical practice often do not provide the expected result. Based on the rarity of the disease it is difficult to come up with a robust homogenously treated patient cohort to draw and interpret conclusions [15]. Therefore, patient-derived cancer cell lines and the respective models are indispensable tools for screening drugs and exploring the biology of the tumor. The use of a suitable cultivation method is crucial, as 2D models represent tissue rather inaccurately in vitro, but are easier to handle, highly reproducible and very cost-efficient [16, 17]. Modelling 3D cell cultures brings significant improvement in terms of visualizing morphology, monitoring proliferation, response to stimuli, differentiation, drug metabolism and protein synthesis [18, 19]. The use of patient-derived models promotes a better understanding of the pathophysiological processes of tumor disease and especially of the metastatic process that is still elusive despite its clinical importance. A suitable model includes patient-derived cell lines with a detailed characterization, a comprehensive clinical description, and an exact therapeutic schedule [20]. The aim of this work was to establish a suitable patient-derived CCS cancer model to investigate the methylation of a primary CCS and lung metastasis cells from the same patient in detail. MUG Lucifer prim and MUG Lucifer met combined with healthy control cells from the same patient represent an appropriate CCS study model and enable translational research in terms of an innovative treatment strategy and investigation of the metastatic process.

## Methods

### Patient history

A 23-year-old woman was diagnosed with a CCS invading her left knee joint in December 2018. A biopsy revealed a CCS involving the *EWSR1::ATF1*-fusion gene. In January 2019, extra articular left knee resection was subsequently performed by the Department of Orthopedics and Trauma at the Medical University of Graz. Tumor tissue obtained from this surgery was used to establish MUG Lucifer prim and a piece of skin served to isolate non-tumorigenic control cells. In October 2019, the patient developed large soft tissue and lymph node metastases in the left groin and pelvis. At this point no further metastases could be detected and based on massive pain and increased risk of ulceration of the metastatic lesions the patient underwent a hemipelvectomy in December 2019. Additionally, a treatment with the tyrosine kinase inhibitor (TKI) crizotinib was initiated. In April 2020, the patient showed a disease progression with lung metastases, bone metastases and pleura sarcomatosis on both sides, and she received a palliative chemotherapy with doxorubicin. Unfortunately, after one cycle of doxorubicin her general condition deteriorated rapidly. In May 2020, she developed thoracic soft-tissue metastases and mediastinal lymph node metastases. Pleural fluid samples were taken in June 2020 and used for the establishment of MUG Lucifer met. The patient was treated with trofosfamide until June 2020. She died in the same month due to brain hemorrhage.

### Cell culture

A part of the excised primary tumor sample was available for cell culture to establish MUG Lucifer prim. The material was morphologically divided into tumor tissue and surrounding tissue. Both specimen were incubated for 10 min in a 10% concentrated penicillin (10000 U/mL) / streptomycin (10000 U/mL) (pen/strep) (Gibco, Life Technologies, Darmstadt, Germany) antibiotic bath in phosphate buffered saline (Gibco, Life Technologies; PBS) to avoid possible bacterial contamination. Afterwards, the pieces were dissociated using the human tumor dissociation kit (Miltenyi Biotec, Bergisch Gladbach, Germany), cultivated with DMEM/F12 phenol red free medium (Gibco, Life Technologies) supplemented with 10% FBS, 2 mM l-glutamine (Gibco, Life Technologies) and treated with 1% pen/strep during growth but not in the subsequent experiments. For the establishment of MUG Lucifer met, pleura fluid was collected from the patient, centrifuged (300 g for 4 min) and incubated with 10% concentrated pen/strep antibiotic bath in PBS for 10 min to avoid bacterial contamination. For a better comparability of all experiments, both cell lines were cultured in the same culture medium. A piece of healthy skin from the same patient was available for cell culture. After dissecting the skin in 1–2 mm^2^-sized pieces with a scalpel, the small pieces were incubated for 10 min in a 10%  pen/strep antibiotic bath in PBS to avoid bacterial contamination. Skin pieces were transferred into a 25 cm^2^ cell culture flask and cultured in DMEM (Gibco, Life Technologies) supplemented with 10% FBS, 2 mM l-glutamine and 1% pen/strep. Dermal fibroblasts were immortalized using the human telomerase reverse transcriptase (hTERT) technology. In brief, human dermal fibroblasts were seeded to a density of 50–70% and transduced with EX-Q0450-Lv201 (GeneCopoeia, Rockville, MD, US). Polybrene (Sigma Aldrich, Darmstadt, Germany) was added with a final concentration of 8 µg/ml and lentiviral enhancer (ABM, Richmond, Canada) in a dilution of 1:200. 1 µg/ml puromycin (Gibco, Life Technologies) was used for selection. All cells were maintained in a humidified incubator set to 5% CO_2_ atmosphere at 37 °C and regularly checked for mycoplasma. Both tumor cell lines were cultivated above passage 80 to confirm indefinite proliferation. To verify the tumor entity, original tumor samples, xenografts and both cell lines underwent immunhistochemistry (IHC) stainings HE, SOX10, S100 and HMB45. Cell culture microscopic pictures were taken at room temperature on an Eclipse Ti2 inverted microscope (Nikon, Tokyo, Japan), 10 × magnification, and numerical aperture 0.30 with a DS-Fi2 camera (Nikon, Tokyo, Japan). Pictures were analyzed with the NIS-Elements BR 5.02.00 software (Nikon, Tokyo, Japan).

### Cell line authentication

DNA from both, tumor tissue and cells, was prepared using the QIAamp DNA Mini kit (Qiagen, Hilden, Germany) in accordance with the manufacturer’s protocol. 0.5 ng purified DNA of each sample was amplified using the Power Plex^®^ 16 HS System (Promega Corporation, Walldorf, Germany). 1 μl of the product was mixed with Hi-Di formamide (Applied Biosystems Inc., Foster City, CA, USA) and Internal Lane Standard (ILS600), denatured and fractionated on an ABI 3730 Genetic Analyzer (Applied Biosystems Inc.). The resulting data were processed and evaluated using ABI Genemapper 3.7.

### Electron microscopy

MUG Lucifer prim and met cells were grown on an Aclar film (Gröpl, Tulln, Austria), fixed in 2.5% (wt/vol) glutaraldehyde and 2% (wt/vol) paraformaldehyde in 0.1 M cacodylate buffer, pH 7.4, for 2 h, postfixed in 2% (wt/vol) osmium tetroxide for 2 h at room temperature, dehydrated in graded series of ethanol and embedded in TAAB (Agar Scientific, Essex, GB) epoxy resin. Ultrathin (70 nm thick) sections were cut with a UC 7 Ultramicrotome (Leica Microsystems, Vienna, Austria) and stained with lead citrate for 5 min and with platinum blue for 15 min. Images were taken using a Tecnai G2 20 transmission electron microscope (Thermo Fisher Scientific, Waltham, MA, USA) with a Gatan ultrascan 1000 charge coupled device (CCD) camera (temperature −20 °C; acquisition software Digital Micrograph; Gatan, Munich, Germany). Acceleration voltage was 120 kV.

### Soft Agar Colony Formation (SACF) assay

The SACF assay assesses the ability of cancer-derived cells to grow without anchoring to a solid surface and/or their neighboring cells. This so-called anchorage-independent growth is characteristic for malignant transformation in cells and a major aspect for the tumor phenotype in general, especially with respect to metastatic potential [21]. Experiments were performed according to already published protocols [22]. In brief, in six-well plates MUG Lucifer prim and MUG Lucifer met were seeded in 0.3% agarose with medium at a density of 2000 cells per well on top of a bottom layer containing 0.6% agarose in medium. 1 ml of media was added on top of the agarose containing layers in order to prevent desiccation. Cells were cultured for three weeks including top layer media changes once a week. Colonies were stained with 0.01% crystal violet (in 10% ethanol) and destained by washing with ddH_2_O for several hours.

### Tumorigenicity study

CR ATH HO mice (Crl:NU(NCr)-Foxn1nu, Charles River Laboratories, Kent, UK) were maintained in-house (4–5 weeks of age, weight between 15 and 20 g). All mice were maintained under specific pathogen free (SPF) conditions in individually ventilated cages with *ad libitum* access to food and water. For cell injection and ultrasound imaging, mice were anesthetized with constant administration of 2% isoflurane in a constant airflow of 2.5 l per minute. In the pilot experiment, MUG Lucifer cell lines were injected in five mice (7 × 10^6^ cells in 100 µl PBS, MUG Lucifer prim in the right flank, MUG Lucifer met in the left flank). In the second experiment, the same amount of cells was injected, but using five mice each for MUG Lucifer prim and MUG Lucifer met. Inoculation of the tumor was monitored daily and ultrasound imaging started on day six post-injection once a week. After sacrification, histopathological examination was performed. The mice were dissected and the tumors extracted. All other organs were checked visually for structural changes. The tissue was then fixed in 4% paraformaldehyde solution for 24 h and further embedded in paraffin.

### Ultrasound imaging

High-frequency ultrasound (HF-US) was performed using a Vevo3100 HF-US system (Fujifilm VisualSonics Inc., Toronto, Canada) with a 50 MHz (MX700) or a 40 MHz (MX550D) transducer (Fujifilm VisualSonics Inc.) reaching a spatial resolution of approximately 30–40 µm. Sagittal and transversal images of the region of interest were obtained. Calculation of tumor volume was performed with VevoLab Software (Version 5.5.1, Fujifilm VisualSonics Inc.) using the formula displayed in Eq. 1 (Tumor volume calculation).

1 $$V\left[{mm}^{3}\right]=\frac{3.141*length\left[mm\right]*width\left[mm\right]*depth\left[mm\right]}{6}$$

### DNA methylation

DNA from cells was prepared using the QIAamp DNA Mini kit (Qiagen, Hilden, Germany) in accordance with the manufacturer’s protocol. After DNA concentration determination using NanoDrop Spectrophotometer (Thermo Fisher Scientific), samples were stored at −20 °C until measurement. Methylation of genomic DNA was quantified using Infinium Methylation EPIC BeadChip assay (Illumina, San Diego, CA, USA) which covers 865,918 CpGs [23]. After bisulfite-converting the genomic DNA samples with Zymo EZ DNA Methylation-Kit (Zymo Research, Orange, CA, USA), pre- and post-amplification laboratory protocols were performed according to the Infinium HD methylation Assay Guide version 15019519v07. Images were captured with the Illumina iScan. Raw methylation score for each CpG site expressed as β-values was calculated (β = intensity of the methylated allele (M)/intensity of the unmethylated allele (U) + intensity of the methylated allele (M) + 100) with Genome Studio software (version 2011.1). The output IDAT files were processed using ChAMP data package (version 2.20.1). After loading IDAT files, several filtering steps were preformed: Discarding the sample with a detection p > 0.01; filtering out probes with < 3 beads in at least 5% of samples per probe and all non-CpG probes contained in this dataset; excluding all SNP-related probes, multi-hit probes or probes located in chromosome X and Y [24]. Next, normalization with BMIQ function was conducted to adjust for the effects of type-I/type-II probe bias. To account for multiple testing and reduce the number of false positives, the false discovery rate (FDR) on the genome-wide analysis of DNA methylation (FDR < 0.01) was applied.

### Arginine methylation measurements

For ArgMet determination, MUG Lucifer prim and MUG Lucifer met cells were seeded in 175 cm^2^ cell culture flasks, each at a density of 1 × 10^7^ cells for treatments and 5 × 10^6^ cells for negative controls, and treated with the respective IC_50_ of GSK 3368715 for up to six days, periodate-oxidized adenosine (AdOx, Sigma Aldrich, Darmstadt, Germany), a methyltransferase inhibitor, for up to 72 h and appropriate negative controls at 37 °C in a humidified atmosphere containing 5% CO_2_. Cells were harvested, washed with PBS once and snap frozen. Samples were stored at −80 °C until NMR analysis.

Samples for quantification of arginine and methylated arginines were prepared as described elsewhere [14, 25]. Briefly, the precipitates were hydrolyzed with HCl to obtain (modified) amino acids, lyophilized and resuspended in 0.1 M HCl and chloroform to remove lipids, centrifuged and the supernatant subjected to solid-phase-extraction (SPE). Arginine and its derivatives were eluted, lyophilized and dissolved in 500 µl NMR buffer [0.08 M Na_2_HPO_4_, 5 mM 3-(trimethylsilyl) propionic acid-2,2,3,3-d_4_ sodium salt (TSP), 0.04 (w/v) % NaN_3_ in D_2_O, pH adjusted to 7.4 with 8 M HCl and 5 M NaOH] for NMR measurements.

All NMR experiments were carried out as described by Zhang et al*.* and Habisch et al. [14, 25]. 2D JRES (^1^H homo-nuclear J-resolved spectroscopy) spectra were acquired at 310 K on a Bruker 600 MHz Avance Neo spectrometer equipped with a TXI probe head and using the jresgpprqf pulse sequence (16 scans, size of fid 16384 (direct dimension F2)/256 (indirect dimension F1), 10000.00/78.042 Hz spectral width in F2 (chemical shift axis)/F1 (spin–spin coupling axis), recycle delay 2 s) with presaturation during the relaxation delay to obtain virtually decoupled spectra. Data were processed in Bruker Topspin version 4.0.9 using the SINE and QSINE window functions (SSB = 0) in F2/F1. Fourier transform was performed with 16384/256 F2/F1 points of the fid. 2D J-resolved experiments were processed using back prediction implemented in the Bruker au program proc_jres.be [26]. The JRES spectra were then projected along F2 and exported as 1D NMR spectra. Data of ^1^H 1D projections of 2D J-resolved, virtually decoupled NMR spectra were extracted using Matlab^®^ vR2014b (Mathworks, Natick, MA, USA) and quantification of arginine, MMA, ADMA and SDMA was carried out by integration of characteristic peaks as described elsewhere [14, 25].

### Viability and apoptotic determination for 2D cell culture

#### CellTiter 96^®^ aqueous non-radioactive cell proliferation assay (MTS)

To determine the IC_50_ of the inhibitors GSK 3368715, GSK 591 and AdOx, MUG Lucifer prim und MUG Lucifer met cells were each seeded in clear 96-well plates (Corning Incorporated, Corning, NY, USA) at a density of 2 × 10^4^ cells per well. The cells were treated with GSK 3368715 (MCE MedChemExpress, Monmouth Junction, NJ, USA), an uncompetitive type I PRMT inhibitor, in a concentration range of 0–10 µM for up to six days; with GSK 591 (MCE MedChemExpress), a potent and selective inhibitor of PRMT5, in a concentration range of 0–10 µM for up to six days; and with AdOx in a concentration range of 0–20 µM for up to three days in a humidified 5% CO_2_ atmosphere at 37 °C; appropriate negative controls were used. 20 µl of combined MTS/PMS solution (Promega Corporation) were added to each well. After 1–2 h incubation at 5% CO_2_ and 37 °C in a humidified atmosphere, absorbance was measured at 490 nm and 720 nm for correction of wavelength on a CLARIOstar microplate reader (BMG Labtech, Ortenberg, Germany). IC_50_ determination for crizotinib (MCE MedChemExpress) was performed in an analogous manner. Concentrations used ranged from 0.01–100 µM and incubation time was only 48 h. Three biological replicates of all experiments were performed, each in three technical replicates.

#### ApoTox-Glo™ triplex assay

To determine the viability, cytotoxicity, and late apoptosis simultaneously in 2D cell culture, ApoTox-Glo^™^ Triplex assays (Promega Corporation) were performed. Cells were seeded in white-walled 96-well plates (Corning Incorporated) using 2 × 10^4^ cells per well and treated with IC_50_ of PRMT inhibitors and dimethyl sulfoxide (DMSO) using the same concentrations and medium as controls. MUG Lucifer prim was treated with GSK 3368715 for six days and MUG Lucifer met was treated with AdOx for 72 h, as the inhibitors did not show any remarkable inhibition reversely. After incubation, 20 µl of viability/cytotoxicity reagent were added to each well and the plate was incubated for 30 min at 37 °C in a humidified atmosphere containing 5% CO_2_. Fluorescence was measured at 400_Ex_/505_Em_ for viability determination and at 485_Ex_/520_Em_ for cytotoxicity determination using CLARIOstar microplate reader. Accordingly, the plate was incubated for 30 min at RT after adding 100 µl of Caspase 3/7 Glo reagent to each well for late apoptosis assessment. Changes in apoptosis were determined by comparing the luminescence of treated samples to vehicle controls.

#### Annexin V/PI staining for flow cytometry

For early apoptosis assessment in 2D cell culture, cells were stained with Annexin V and propidium iodide (PI) using FITC Annexin V/Dead Cell Apoptosis Kit (Thermo Fisher Scientific). Cells were treated with IC_50_ + 20% PRMT inhibitor and DMSO using the same concentration as a vehicle control. Cells were harvested with Accutase Cell Dissociation Reagent (Thermo Fisher Scientific) after appropriate incubation time (six days for GSK 3368715 and 72 h for AdOx), and stained with Annexin V and PI according to the manufacturer’s protocol. Cell suspensions were measured within a short period of time using the CytoFLEX S Flow Cytometer (Beckman Coulter, Krefeld, Germany). Data were analyzed using CytExpert Software (version 2.3, Beckman Coulter).

### Viability and apoptotic determination for 3D cell culture

#### CellTiter-Glo^®^ 3D cell viability assay

To determine viability in 3D cell culture, CellTiter-Glo^®^ 3D cell viability assays (Promega Corporation) were performed. Cells were seeded in an ultra-low attachment (ULA) 96-well plate (Corning Incorporated) using 2500 cells per well. After 24 h, cells were treated with GSK 3368715 and the respective DMSO control for six days. After incubation, spheroids were transferred into a white 96-well plate (Corning Incorporated) with wide bore pipet tips (ThermoFisher Scientific) using a volume of 80 µl and 80 μl of CellTiter-Glo^®^ 3D Reagent was added to each well and the plate incubated for 30 min at RT. After incubation time, luminescence was measured using CLARIOstar microplate reader.

#### Caspase-Glo® 3/7 assay

To determine late apoptosis in 3D cell culture, Caspase-Glo^®^ 3/7 assays (Promega Corporation) were conducted. Cells were seeded in ULA 96-well plates to form spheroids subsequently using 2500 cells per well. After 24 h, cells were treated with GSK 3368715 and the respective DMSO control for six days. After incubation, spheroids were transferred with wide bore pipet tips into a white 96-well plate using a volume of 80 µl and 80 μl of Caspase-Glo^®^ 3/7 reagent was added to each well and the plate was incubated for 30 min at RT. After incubation time, luminescence was measured using CLARIOstar microplate reader.

### Copy number profiling

Genome-wide copy number alterations (CNA) were established using shallow whole genome sequencing (sWGS). Shotgun libraries were prepared using the TruSeq Nano HT Sample Preparation Kit (Illumina, San Diego, CA, USA). Briefly, 330 ng of MUG Lucifer prim tissue and MUG Lucifer met tissue and 110 ng of the respective cell lines were fragmented in a total volume of 130 µl 1 × TE (pH 8.0) using the Covaris System (Covaris, Woburn, MA, USA). After concentrating the volume to 50 µl using a SpeedVac (Eppendorf, Hamburg, Germany) end repair, A-tailing and adapter ligation were performed following the manufacturer’s instructions. For selective amplification of the library fragments that have adapter molecules on both ends, 15 PCR cycles were used. Libraries were quality checked on an Agilent Bioanalyzer using a DNA 7500 Chip (Agilent Technologies, Santa Clara, CA, USA) and quantified using qPCR (Applied Biosystems Inc., Foster City, CA, USA) with a commercially available PhiX library (Illumina) as a reference. Libraries were pooled equimolarily and sequenced on an Illumina MiSeq in a 75 bp paired end mode. BCL and FASTQ files were generated onboard using the integrated Illumina BaseSpace software. Genome-wide copy number calling was performed using a probabilistic HMM model called ichorCNA as previously described [27, 28].

### Cell cycle analysis and ploidy determination

Cell cycle experiments were performed by flow cytometry using PI as a DNA staining agent. 5 × 10^5^ of both cancer cells and peripheral blood mononuclear cells (PBMNCs) from buffy coat were harvested by centrifugation (300 g, 4 min) and washed two times with PBS. The obtained pellet was then incubated for 20 min at RT in 200 µl hypotonic lysis buffer (50 µg/ml PI, 0.1% Triton X-100, 0.1% sodium citrate, 100 µg/ml RNAse A). Samples were immediately measured after incubation on a CytoFLEX S flow cytometer. The data files were analyzed using ModFit LT^™^ 5.0.9 (Verity Software House, Topsham, ME, USA). The peaks correspond to the population of nuclei in various phases of the cell cycle. The cell populations were further subdivided into the cell-cycle phases based on the distribution of DNA content following PI staining. PBMNCs behave as reference to identify the position of cells with normal diploid amount of DNA [29]. DNA Index (DI) of each sample is calculated as the mean fluorescence level of G0/G1 tumor cells relative to the G0/G1 peak of normal diploid cells.

### Statistical analysis

Statistical significance was calculated by one-way ANOVA and unpaired t-test with confidence levels of 95% using GraphPad Prism 9 (GraphPad Software, San Diego, CA, USA). All data were reported as mean ± standard deviation, n denoting the number of independent experiments. P < 0.05 were considered as significant (* 0.01 to 0.05, **0.001 to 0.01, *** < 0.001).

## Results

### Cell line establishment and characterization

**Fig. 1:**
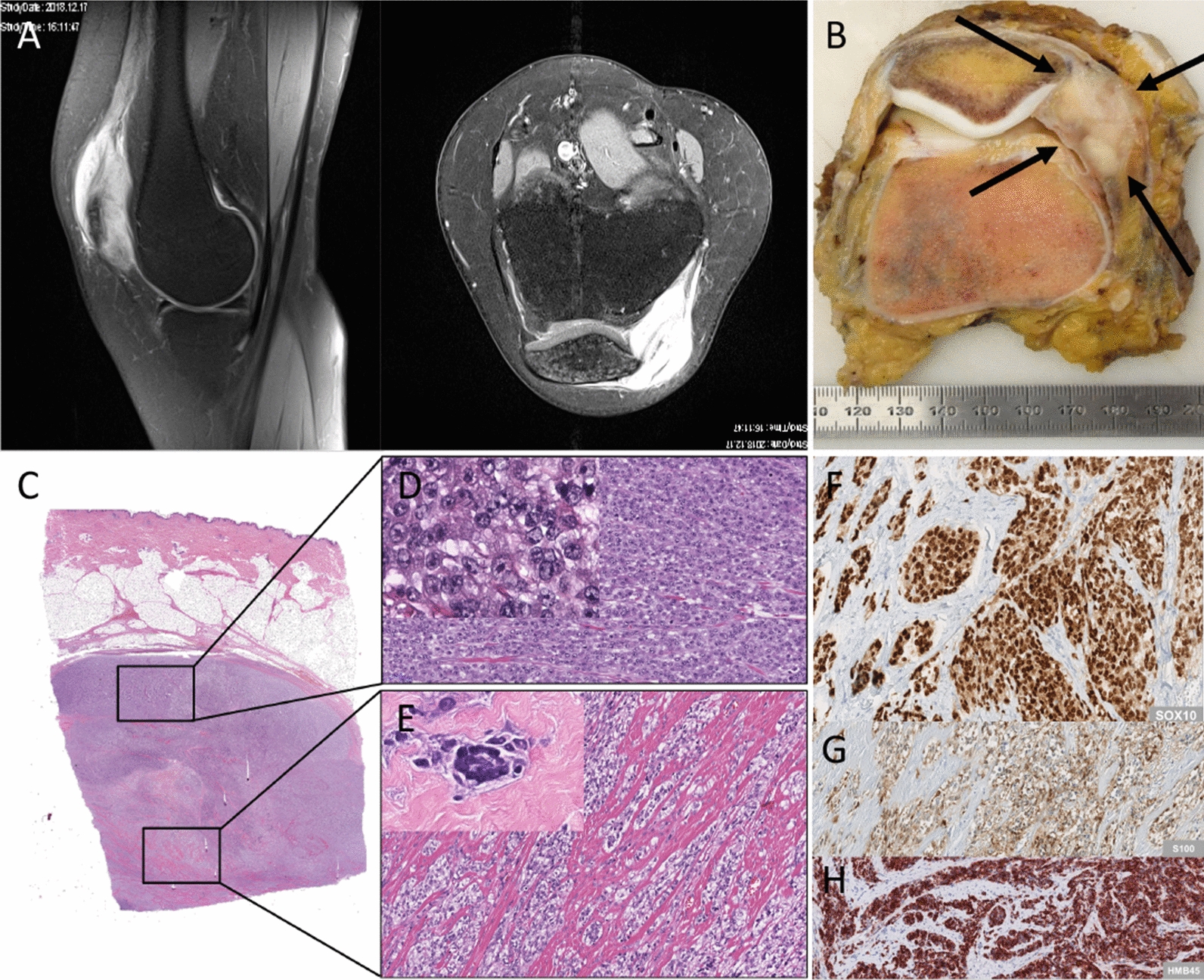
Patient history. Magnet resonance of the left knee with a solid contrast enhancing lesion 6 × 5 × 3 cm on the anteromedial circumference of the knee joint with intra and extra-articular infiltration (**A**). Resection specimen with a multinodular gray soft tissue on the anteromedial aspect of the knee joint with articular infiltration (**B**). HE with an overview of tumor tissue located in the soft tissues. Regular skin, dermis and subcutaneous fat (**C**). Solid sheets of a monotonous proliferation of epithelioid tumor cells with prominent nucleoli and eosinophilic to clear cytoplasm (inset) (**D**). Nests, bundles and fascicle of tumor cells separated by dense fibrous stroma. Focal wreath-like multinucleated giant cells (inset) (**E**). Strong nuclear expression of SOX10 in all tumor cells (**F**). Nuclear and cytoplasmic expression of S100 in tumor cells (**G**). Strong cytoplasmic expression of HMB45 (**H**)

MUG Lucifer prim had been isolated from the surgical specimen of the knee (Fig. 1A–H). First cell growth was observed after only three days in culture. One and a half years later, MUG Lucifer met cells from lung aspirate were isolated from the same patient. Cell line characterization included assessment of cell morphology, DNA ploidy, drug sensitivity as well as tumorigenicity in vitro and in vivo. Both cell lines also underwent IHC stainings and genetic analysis in order to identify further characteristic features.

**Fig. 2:**
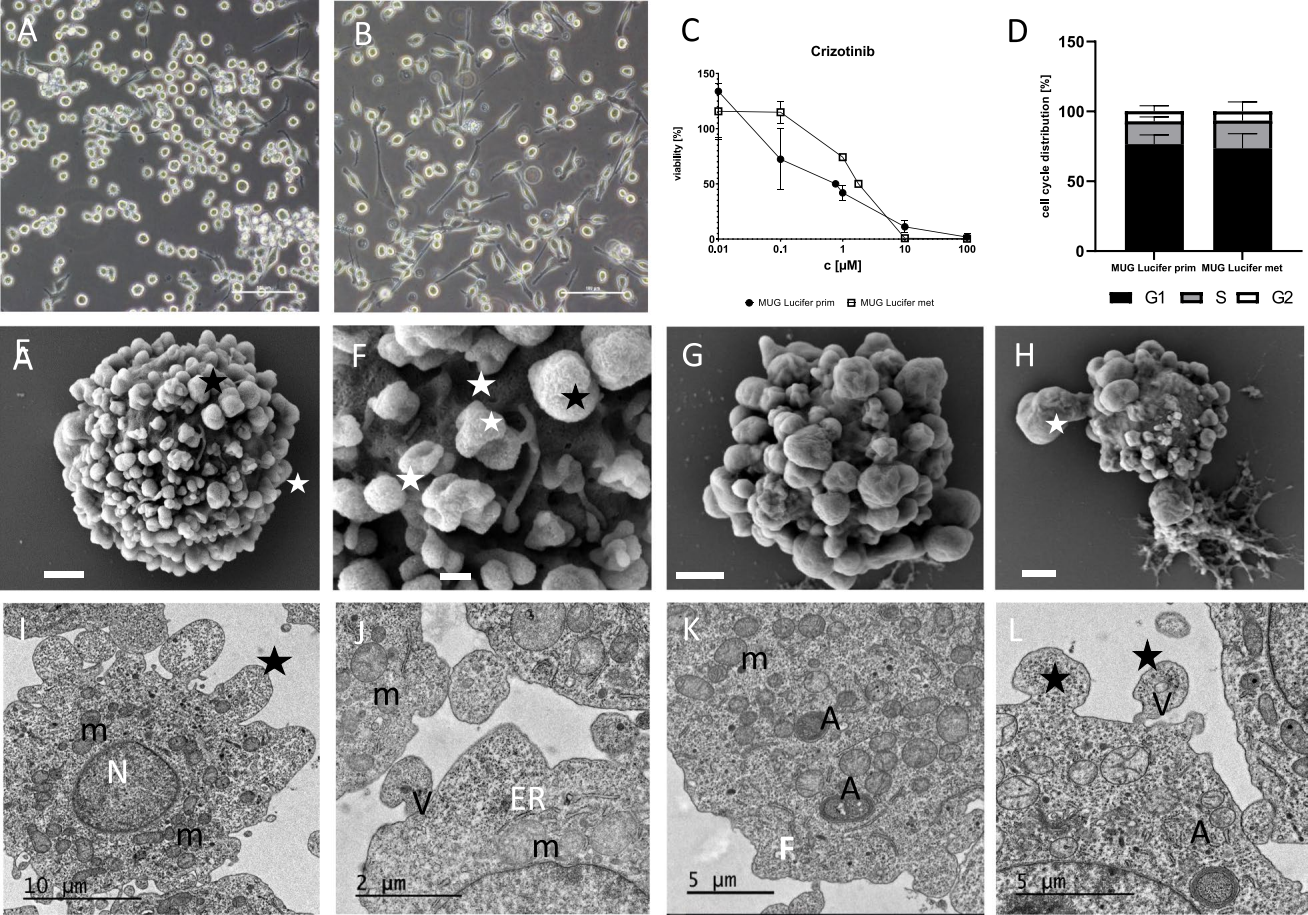
In vitro characterization and electron micrographs of isolated MUG Lucifer cell lines. Morphology of MUG Lucifer prim cells in 200× magnification using light microscopy. Scale bars represent 100 µm (**A**). Morphology of MUG Lucifer met cells in 200× magnification using light microscopy. Scale bars represent 100 µm (**B**). IC_50_ determination of MUG Lucifer cell lines treated with TKI crizotinib. Concentrations of crizotinib between 0.01 and 100 µM were used for cell viability assessment. Obtained values are normalized to the vehicle control (1% DMSO). IC_50_ was calculated using GraphPad Prism 9. Error indicators are the positive and negative standard deviations based on biological replicates (n = 3) (**C**). Cell cycle profiles of MUG Lucifer prim and MUG Lucifer met analysis by ModFit LT^™^ 5.0.9 (**D**). Scanning electron micrographs of MUG Lucifer prim and MUG Lucifer met cells; **E** and **F** show MUG Lucifer prim cells with the extended surface (asterisk), scale bar represents 1 µm. Micrograph F indicates at higher magnification the close network of these extensions (asterisk), scale bar represents 200 nm. Micrograph **G** and **H** show the MUG Lucifer met cells with similar surface, scale bar represents 2 µm; in micrograph H the dimension of these extension is clearly visible (asterisk), scale bar represents 1 µm. Transmission electron micrographs of MUG Lucifer prim (**I**). Looking closer, cells show a centrally located nucleus (N), a cluster of mitochondria (m) spread through the whole cell in micrograph **J**. Endoplasmatic reticulum (ER) next to mitochondria, small vesicles within these extensions (V) in micrograph **K**. In micrograph **L** autophagosomes (A) within the cells and extensions (asterisk) with different kinds of vesicles (V) are depicted at higher magnification

Morphology of both tumor cell lines presented small round cells and polygonal spindle-shaped cells, with round nuclei containing extremely prominent nucleoli (Fig. 2A–B and I), both growing in semi-adherent behavior. As soon as continuous growth had been achieved, IHC staining was performed. HE staining presented spindle-shaped tumor cells with mitoses and IHC staining positivity for SOX10 (Additional file 1: Figure S2I–J). When establishing 3D models, both lines showed a loose 3D structure with an uneven surface and loose cells, whereas by co-cultivating the CCS cells with patient-derived fibroblasts compact spheroids are presented (Additional file 1: Figure S2A–H). By using electron microscopy, both cell lines revealed characteristic surface structures similar to serrations (Fig. 2E–H). Higher resolution electron microscopy images for detailed investigation of these serrations showed large amounts of glycogen, but no premelanosomes or melanosomes in their cytoplasm. A strikingly large number of mitochondria and autophagosomes was detected in both cell lines (Fig. 2I–L, Additional file 1: Figure S1).

**Fig. 3:**
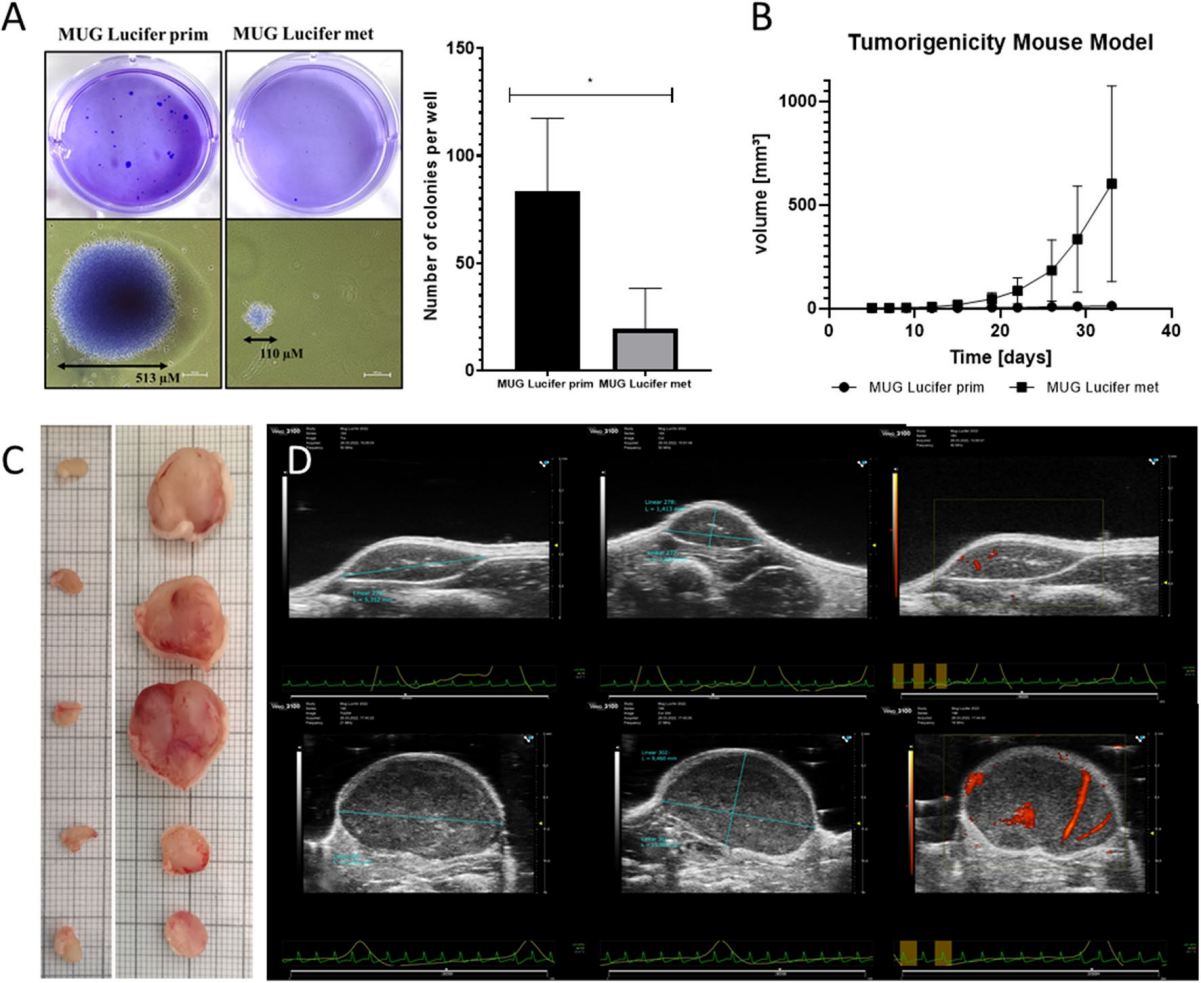
Tumorigenicity potential of MUG Lucifer cell lines. In vitro tumorigenicity determined by SACF. Differences in colony number and size were observed when compared to each other. Results represent the mean and standard deviation of three independent experiments (**A**). Tumorigenicity mouse model: plot of tumor increase in volume correlated to time of both cell lines (**B**); visualization of the excised tumors macroscopically (**C**) and by using ultrasound-imaging technology (**D**) of MUG Lucifer prim (**C**: left, **D**: top) and MUG Lucifer met (**C**: right, **D**: bottom)

Drug sensitivity of the cell lines served as another factor to classify and distinguish primary and metastatic cell line. MTS assays determined cell viability upon treatment with the TKI crizotinib in a concentration ranging from 0.01 µM to 100 µM, with 1% DMSO serving as a vehicle control. An IC_50_ of 0.77 µM was determined for MUG Lucifer prim, whereas an approximately two-fold higher IC_50_ was observed in the metastatic lesion (Fig. 2C). Subsequently, patient-derived cell lines were compared in terms of their potential to form colonies and their tumorigenicity in vivo. Interestingly, MUG Lucifer prim presented colonies larger in diameter and number per well compared to MUG Lucifer met (Fig. 3A). For the in vivo xenograft studies the same cell amount for both cell lines, namely 7 × 10^6^ cells, were used for better comparability. Both cell lines were injected into five mice (MUG Lucifer prim in the right flank, MUG Lucifer in the left flank) (Additional file 1: Figure S3) for the pilot experiment. In the second experiment, MUG Lucifer prim was applied to five mice and MUG Lucifer met to another five mice. MUG Lucifer met started to grow on day 20, whereas MUG Lucifer prim eight days later (Fig. 3B, C and Additional file 1: Figure S3) in both experiments confirming reproducibility of the results. By using high-resolution ultrasound, the tumors could be accurately detected in vivo. In addition, the blood vessels were also visualized. MUG Lucifer met showed significantly more blood supply compared to MUG Lucifer prim (Fig. 3D). Both xenografts recapitulated the morphology as well as the IHC marker profile of the patient’s tumor (Additional file 1: Figure S4).

**Fig. 4:**
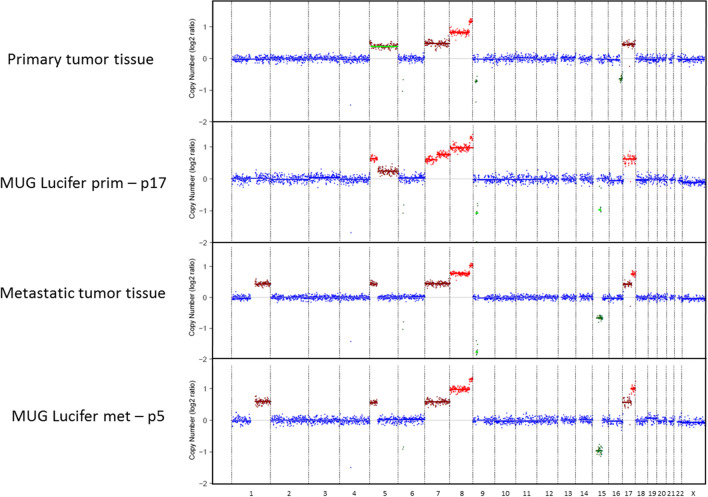
Copy number profiles of MUG Lucifer cell lines. Genome wide log2 ratios are shown for MUG Lucifer prim and MUG Lucifer met tumor tissues and corresponding passages p17 and p5 for each chromosome. Dots represent log2 ratios of genomic bins corresponding to a size of average 56 kb, where green, red and blue indicate regions with log2 ratios of −0.2 to 0.2; > 0.2 or < −0.2 respectively

At DNA level, STR analysis, CNA profiling and cell cycle analysis served as characterizing and cell line authentication tools. To authenticate the cell lines, STR analyses were performed which presented a 100% match in both cell lines from 16 loci; on CSF1PO, a loss of one allele [10] after higher passage (> 50) compared to corresponding tumor tissue was noted (Additional file 2: Table S1). In general, cells with a DNA index greater than one indicate an aneuploid DNA content, which in turn is a characteristic of malignancy and correlates with tumor staging [30]. DNA index was calculated using ModFit LT^™^ 5.0.9 software for cell cycle analysis. DNA indexes of 1.29 and 1.25 were calculated for MUG Lucifer prim and MUG Lucifer met, respectively. Both DNA indexes correspond to a hyperploid cell cycle. Percentage distribution of the cell cycle phases is portrayed in Fig. 2D. Representative cell cycle histograms of spiked patient-derived cell lines with PBMNCs are presented in Additional file 1: Figure S6. In order to characterize the patient-derived cell lines at a genetic level, copy number profiles were established using sWGS. Tissue/aspirate and corresponding lines showed an almost identical CNA profile, which clearly indicates a common origin of all samples (Fig. 4, Additional file 2: Table S2). In primary tissue and resulting cell line, chromosome arm and whole chromosomal gains and amplifications were found at chr. 5, 7, 8 and 17, while losses were present only focally. Metastatic tissue and resulting cell line presented gains and amplifications of chr. 1q, 5p, 7, 8, 17 and loss of chr. 15p.

For a better overview, the results of the characterization of MUG Lucifer prim and MUG Lucifer met are summarized in Table 1.

**Table 1:** Overview of the characterization of MUG Lucifer cell lines

	MUG Lucifer prim	MUG Lucifer met
Growth properties	Semi-adherent, more cells in suspension	Semi-adherent, more adherent cells
Gene fusion	*EWSR1::ATF1*	*EWSR1::ATF1*
CNA profile	Gain in Chr 5, 7, 8, 17	Gain in Chr 1, 5, 7, 8, 17; loss in Chr 15
IHC marker	S100, Sox10, MelanA, HMB45	S100, Sox10, MelanA, HMB45
SACF assay	Bigger and more colonies	Smaller and less colonies
In vivo tumorigenicity	Lower tumor volume and less blood supply, tumor growth starts on day 28	Higher tumor volume and more blood supply, tumor growth starts on day 20

### Epigenetic profiling

**Fig. 5:**
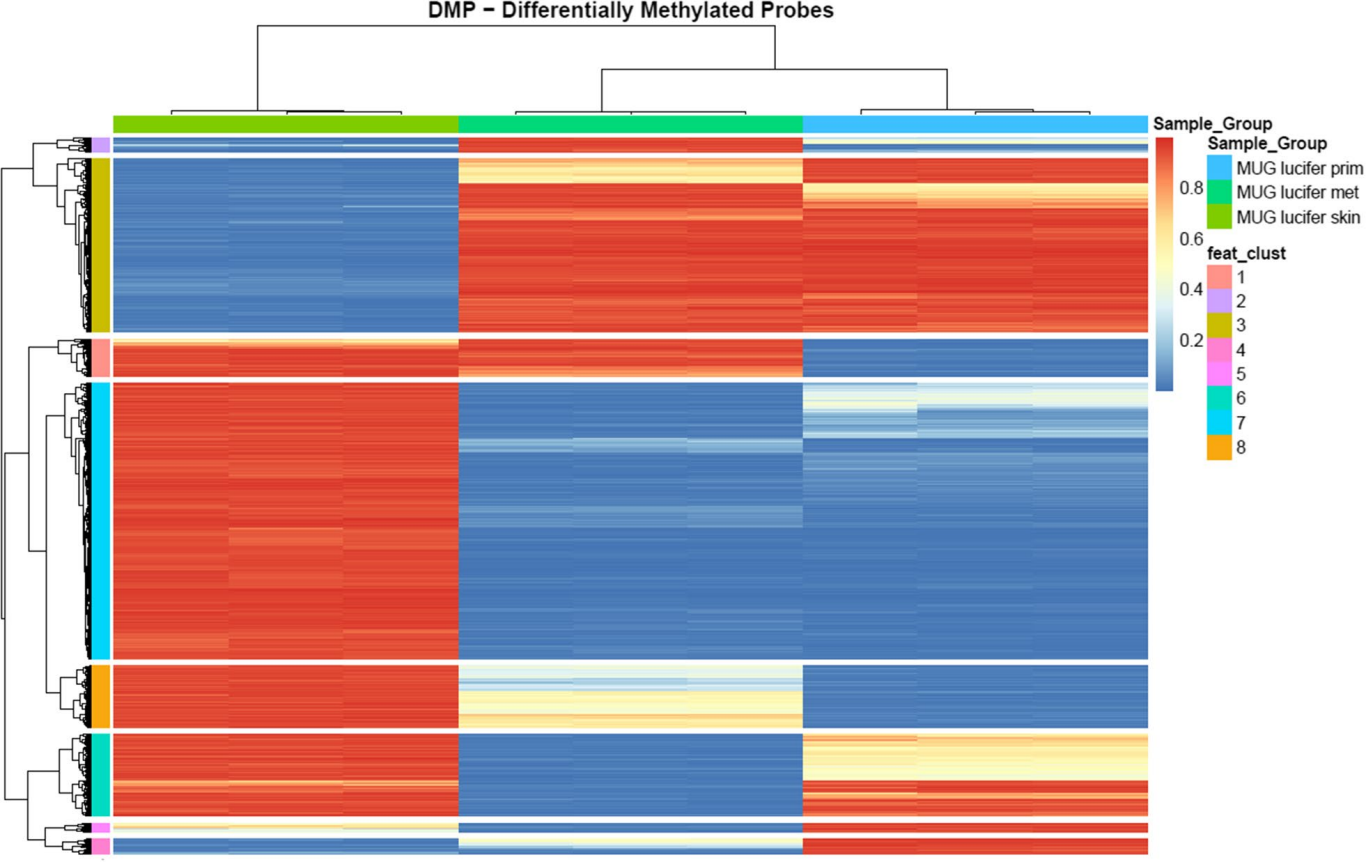
DMP-probe-level heatmap displays eight distinct clusters of differentially methylated CpG sites which are further characterised in Table 2. The largest differences can be observed between skin and the two tumor cell lines (cluster numbers 3 and 7). Nevertheless there are striking similarities between skin and metastasis (cluster number 1) and skin and primary tumor (cluster numbers 2 and 6)

Both hyper- and hypomethylation of DNA are associated with carcinogenesis [31]. DNA methylation profiling with comprehensive coverage of CpG islands, genes and enhancers was carried out and analyzed for the two established CCS cell lines and compared to isolated skin fibroblasts from the same patient. Focusing only on changes from fully methylated to fully unmethylated and vice versa (absolute beta-difference > 0.9 and BH-adjusted p-value cut-off < 0.01) for all three comparisons, a total of 10,017 hyper- and hypo-methylated sites, corresponding to 7,634 unique CpG sites were identified (see Table 2). The numbers of CpG sites and their overlap between the three different comparisons are shown in a heat map of differential methylation probes (DMPs) that significantly segregate skin fibroblasts, MUG Lucifer prim and MUG Lucifer met (Fig. 5). CpG sites were grouped into eight feature clusters by hierarchical clustering. To identify the genomic locations in which differential methylation occurs, DMPs were characterized with respect to CpG islands and gene annotations (see Additional file 1: Figure S5 and Additional file 2: Table S2). Hypomethylation occurs predominantly at a distance from CpG islands (open sea) and in intergenic regions (IGR), whereas hypermethylation is found in CpG islands (and their shores), and at transcription start sites (TSS) and gene bodies.

**Table 2:** Summary of eight feature clusters

Feature cluster	Number of CGs	MUG Lucifer fibroblasts	MUG Lucifer prim	MUG Lucifer met
1	428	High	High	Low
2	162	Low	High	Low-mid
3	1983	Low	Mid-high	Mid-high
4	173	Low	Mid-low	High
5	104	Mid	Low	High
6	938	High	Low	Mid-high
7	3145	High	Low	Low
8	701	High	Mid	Low

Number of cytosine-guanine (CG) dinucleotides compared to MUG Lucifer skin and resulting CCS cell lines

For calculation of differentially methylated regions (DMRs), a focus was placed on the comparison of MUG Lucifer met versus MUG Lucifer prim and MUG Lucifer skin fibroblasts. 36 DMRs were identified with an adjusted p-value of < 0.01. For illustration purposes, probe-level methylation data of only the top 22 DMRs (fwer < 0.05) are summarized in Fig. 6 (in detail Additional file 1: Figures S7-S42). Genes covered by these DMRs include CCDC125, CLDN14, CMYA5, COL4A1, DLG2, FAM83E, KCNIP4, KIF25, MIIR124-2, MIR886, MLC1, OSM, PHOX2B, RAB17, RUNX3, S100P, TINAGL1, TMEM161B.

**Fig. 6:**
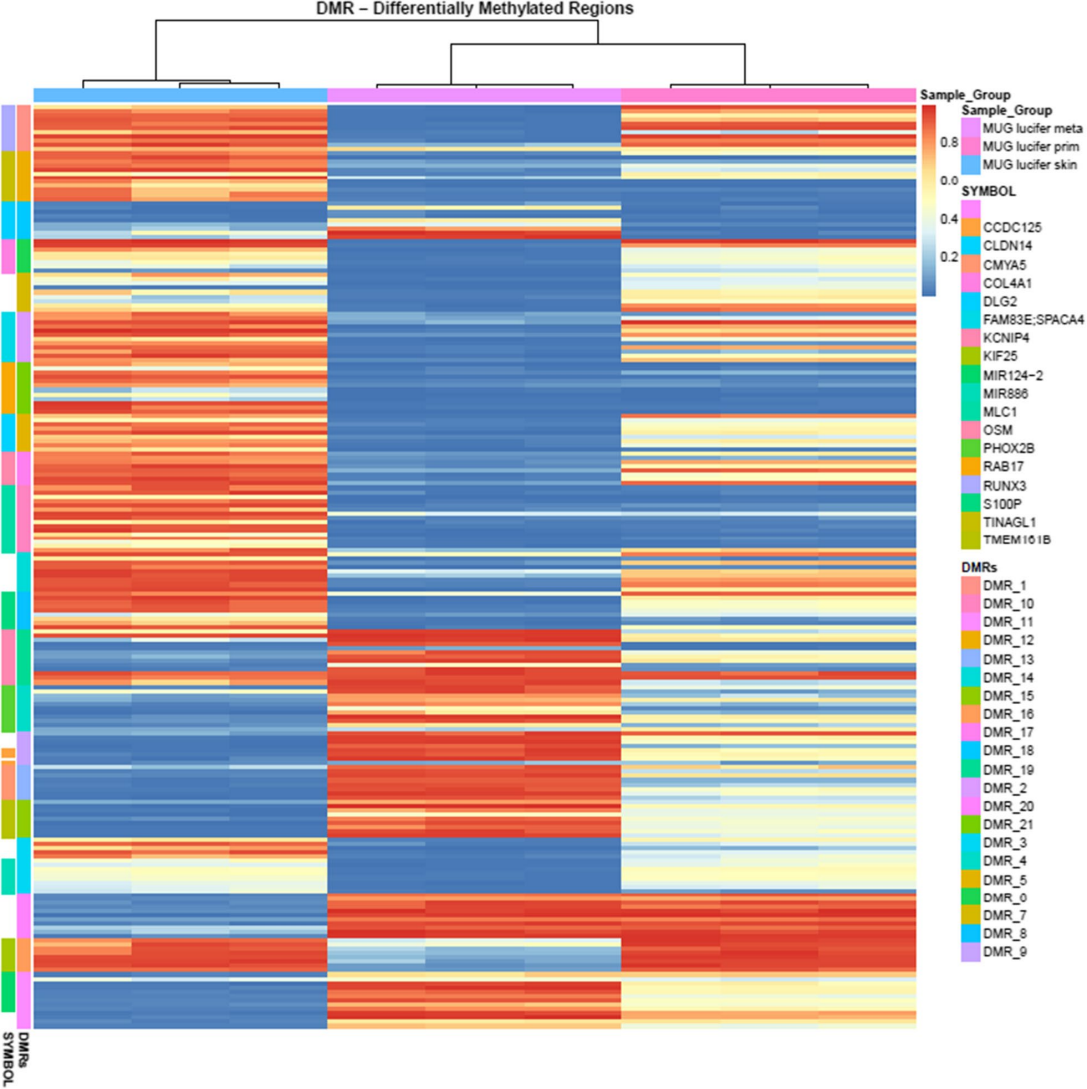
Heatmap of methylation levels of differentially methylated regions (DMRs): Methylation profiles of MUG Lucifer prim and MUG Lucifer met are more correlated to each other than to MUG Lucifer skin fibroblasts. Annotations of DMRs are available in supplementary materials

### NMR-based ArgMet profiling and the use of PRMT1 inhibitors

**Fig. 7:**
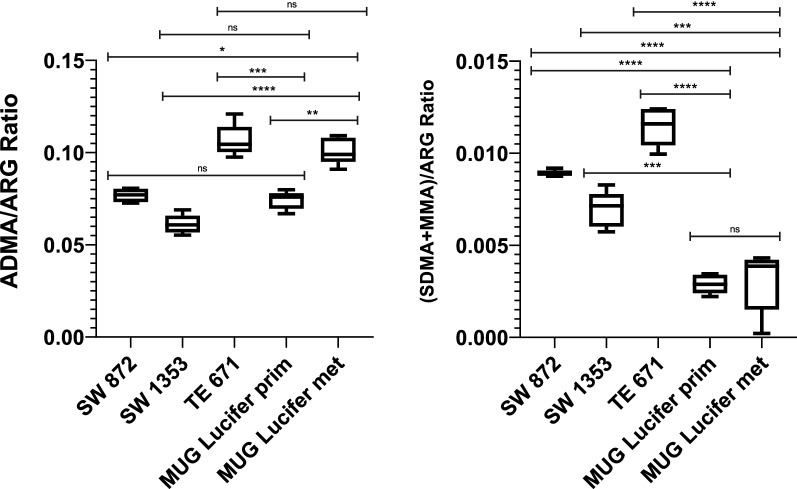
ArgMet profiles of commercially available sarcoma cell lines and patient-derived MUG Lucifer cells characterized by ADMA/ARG and (SDMA + MMA)/ARG ratios

**Fig. 8:**
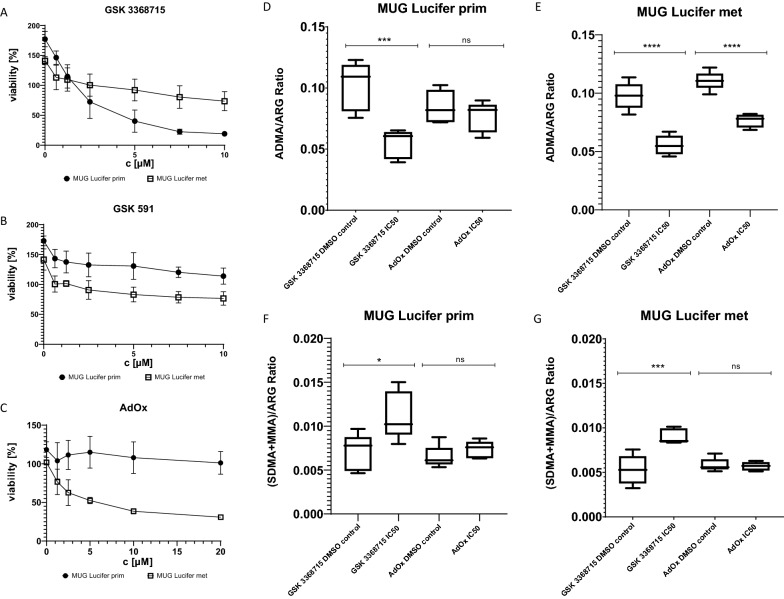
PRMT inhibitor treatment and ArgMet profiles: viability determination of MUG Lucifer prim and MUG Lucifer met cells treated with 0–10 µM GSK 3368715 for six days normalized to the DMSO control. IC_50_ was calculated using GraphPad Prism 9. Error indicators are the positive and negative standard deviations based on biological replicates (n = 3) (**A**). Viability determination of MUG Lucifer prim and MUG Lucifer met cells treated with 0–10 µM GSK 591 for six days normalized to the DMSO control. Error indicators are the positive and negative standard deviations based on biological replicates (n = 3) (**B**). Viability determination of MUG Lucifer prim and MUG Lucifer met cells treated with 0–20 µM AdOx for 72 h normalized to the DMSO control. IC_50_ was calculated using GraphPad Prism 9. Error indicators are the positive and negative standard deviations based on biological replicates (n = 3) (**C**). ADMA/ARG ratios of MUG Lucifer prim cells treated with 3.8 µM GSK 3368715 (IC_50_) and 6.8 µM AdOx (IC_50_) and the respective DMSO controls (**D**). ADMA/ARG ratios of MUG Lucifer met cells treated with 3.8 µM GSK 3368715 (IC_50_) and 6.8 µM AdOx (IC_50_) and the respective DMSO controls (**E**). (SDMA + MMA)/ARG ratios of MUG Lucifer prim cells treated with 3.8 µM GSK 3368715 (IC_50_) and 6.8 µM AdOx, (IC_50_) and the respective DMSO controls (**F**). (SDMA + MMA)ARG ratios of MUG Lucifer met cells treated with 3.8 µM GSK 3368715 (IC_50_) and 6.8 µM AdOx (IC_50_) and the respective DMSO controls (**G**)

As CCSs in general lack genetic aberrations, epigenetic alterations like changes in ArgMet portray the next potential target for anticancer drugs. To assess these changes in MUG Lucifer prim and met and three other sarcoma cell lines, absolute quantification of protein ArgMet was prepared by using NMR spectroscopy. Levels of ADMA, MMA and SDMA are presented normalized to the total arginine content to allow a direct comparison of ArgMet concentrations between patient-derived cell lines and commercially available sarcoma cells. MUG Lucifer met showed a higher ADMA/ARG ratio compared to SW872, SW1353 and MUG Lucifer prim, and a similar level compared to TE671. MUG Lucifer prim exhibited similar ADMA/ARG ratios as SW872 and SW1353, but significantly lower levels than TE671 cells. However, both MUG Lucifer cell lines depicted a lower (SDMA + MMA)/ARG ratio compared to SW872, TE671 and SW1353 (Fig. 7). To verify the importance of epigenetic features regarding cell survival and tumor progression, arginine methyltransferase inhibitors were used. First, AdOx, an adenosine analogue and an indirect methyltransferase inhibitor [32], inhibits adenosylhomocysteine hydrolase (SAHH) which is an important enzyme for the SAM metabolism. It acts as a general methylation inhibitor, blocking all SAM-dependent methylations like ArgMet, LysMet or DNAMet [33, 34]. Second, PRMT inhibitors, namely PRMT Type I inhibitor (ADMA/ARG) GSK 3368715 and PRMT Type II inhibitor ((SDMA + MMA)/ARG) GSK 591 were tested in the present study. Both are, unlike AdOx, specific inhibitors of only protein ArgMet. IC_50_ of the respective inhibitors was detected using concentrations in the range of 0–10 µM for GSK 591 and GSK 3368715 and 0–20 µM for AdOx. After a defined incubation time of three days for AdOx and of up to six days for GSK 3368715 and GSK 591, IC_50_s were calculated. AdOx showed no effect with the used concentrations on MUG Lucifer prim. In contrast, an IC_50_ of 6.8 ± 1.1 µM on MUG Lucifer met was determined (Fig. 8C). GSK 3368715 affected MUG Lucifer prim resulting in an IC_50_ of 3.8 ± 1.5 µM but no effect on MUG Lucifer met (Fig. 8A). GSK 591 presented no effect in the chosen concentration range on both cell lines (Fig. 8B). To verify the potential of GSK 3368715 on ADMA/ARG and (SDMA + MMA)/ARG, levels were re-assayed using NMR spectroscopy after treatment. Results revealed a significant decrease of ADMA/ARG levels and an increase of (SDMA + MMA)/ARG levels in MUG Lucifer prim (Fig. 8D, F). Surprisingly, significantly reduced ADMA/ARG and decreased (SDMA + MMA)/ARG levels were observed, after GSK 3368715 treatment, in MUG Lucifer met as well despite of the missing growth inhibition in 2D experiments (Fig. 8E, G).

### Effect of PRMT inhibitors on patient-derived cell lines

**Fig. 9:**
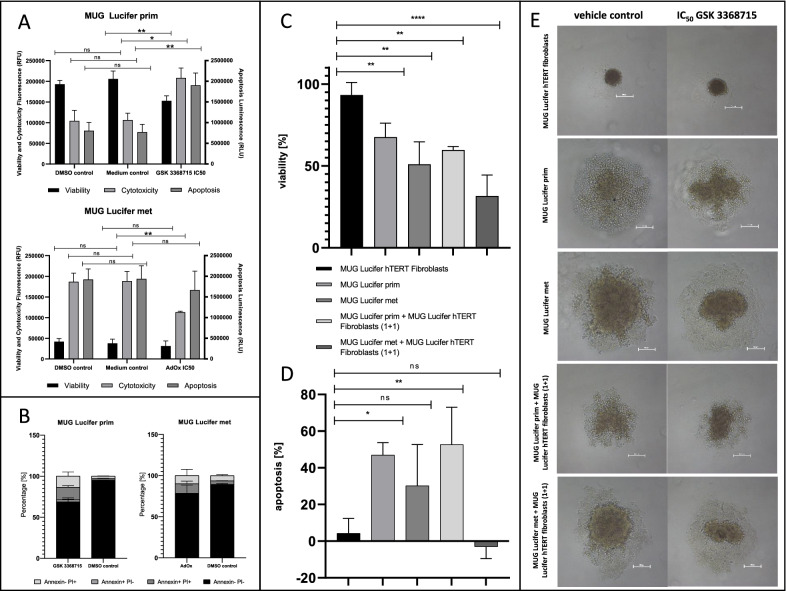
Viability and apoptosis assays in 2D and 3D cell culture. 2D fluorescence/luminescence-based viability, cytotoxicity and apoptosis determination of MUG Lucifer prim cells treated with 3.8 µM GSK 3368715 (IC_50_) for six days and MUG Lucifer met cells treated with 6.8 µM AdOx (IC_50_) for 72 h including the respective DMSO and medium controls (**A**). Early apoptosis determination of MUG Lucifer prim cells treated with IC_50_ + 20% GSK 3368715 (4.56 μM) for six days and MUG Lucifer met cells treated with IC_50_ + 20% AdOx (8.12 μM) for 72 h in 2D cell culture using Annexin V/PI staining for flow cytometry. Annexin- PI + represent necrotic cells, Annexin + PI- cells represent early apoptotic cells, Annexin + PI + represent late apoptotic cells and Annexin- PI- cells represent viable cells (**B**). Viability determination of MUG Lucifer prim, MUG Lucifer met, MUG Lucifer hTERT fibroblasts and co-cultures of MUG Lucifer prim with MUG Lucifer hTERT fibroblasts (1 + 1 ratio) and MUG Lucifer met with MUG Lucifer hTERT fibroblasts (1 + 1 ratio) spheroids treated with IC_50_ + 20% GSK 3368715 (4.56 μM) for six days normalized to the respective DMSO control (DMSO control = 100% viability) using CellTiter-Glo^®^ 3D Cell Viability Assay (**C**). Apoptosis determination of MUG Lucifer prim, MUG Lucifer met, MUG Lucifer hTERT fibroblasts and co-cultures of MUG Lucifer prim with MUG Lucifer hTERT fibroblasts (1 + 1 ratio) and MUG Lucifer met with MUG Lucifer hTERT fibroblasts (1 + 1 ratio) spheroids treated with IC_50_ + 20% GSK 3368715 (4.56 μM) for six days normalized to the respective DMSO (DMSO control = 0% apoptosis) control using Caspase-Glo^®^ 3/7 Assay (**D**). Error indicators are the positive and negative standard deviations based on biological replicates (n = 3) (**A**–**D**). Spheroids of MUG Lucifer cell lines treated with IC_50_ + 20% GSK 336815 (4.56 μM) for six days (right) and the respective DMSO controls (left). Scale bars represent 100 μm (**E**)

To determine the mode of cell death, the ApoTox-Glo^™^ Triplex assay was performed using MUG Lucifer prim cells treated with 3.8 µM (IC_50_) GSK 3368715 for six days and of MUG Lucifer met cells treated with 6.8 µM AdOx (IC_50_) for 72 h versus vehicle controls. After blank correction, a significant decrease of approximately 20% in viability, an increase of 186% in cytotoxicity and a 136% increase in apoptotic effect were noted for MUG Lucifer prim (Fig. 9A). However, after treatment with 6.8 µM AdOx for 72 h, MUG Lucifer met presented a decrease in terms of cytotoxicity of approximately 40% with constant viability levels and no change in apoptotic signals (Fig. 9B). Annexin V/PI staining was performed to verify the apoptotic signal; a concentration of 4.6 µM (IC_50_ + 20%) GSK 3368715 for six days and 8.1 µM (IC_50_ + 20%) AdOx for 72 h was used (Fig. 9C). To conclude the studies of the effect of GSK 3368715 on the MUG Lucifer cell lines, cell viability and apoptosis were assessed in autologous 3D models. For the 3D models, spheroids of single cultures and co-cultures with the healthy immortalized skin cells, the MUG Lucifer hTERT fibroblasts, were used and results normalized to the vehicle control. In comparison to 2D experiments, GSK 3368715 induced decreased viability levels in both MUG Lucifer spheroid models compared to the healthy fibroblast single culture (Fig. 9D). Also, GSK 3368715 treated spheroids exhibited significantly higher caspase activity levels in MUG Lucifer prim cells and their co-culture with MUG Lucifer hTERT fibroblasts in comparison to the MUG Lucifer hTERT fibroblast single culture, whereas no effect in caspase activities could be observed for the respective MUG Lucifer met models (Fig. 9E).

## Discussion

CCSs are ultra-rare, aggressive and difficult to treat. Due to their low incidence, there is also a lack of suitable in vitro and in vivo models.

In the present study, a primary CCS cell line named MUG Lucifer prim and a metastatic CCS cell line named MUG Lucifer met were established and characterized in detail. Additionally, normal skin fibroblasts from the same patient were immortalized and used as healthy control cells. Both CCS cell lines grow in a semi-adherent state with the primary cell line presenting more suspension cells compared to the metastatic cells, meaning that both lines are morphologically well distinguishable.

Survival and proliferative capability of both cell lines were analyzed by using the SACF assay and in vivo models. Interestingly, SACF assays of MUG Lucifer prim presented bigger and more colonies compared to MUG Lucifer met. However, in vivo, MUG Lucifer met is shown to have a massive growth advantage accompanied by a higher tumor volume and more blood supply compared to MUG Lucifer prim. Most remarkably, both cell lines retain the characteristic *EWSR1::ATF1* fusion in long-term cultivation. According to literature, there are only few published cell lines that possess tumorigenicity in immunodeficient mice and keep the *EWSR1::ATF1* fusion [35].

A movement towards personalized medicine and therapy tailored to individual patients often only takes the primary tumor entity into consideration but not the progression or metastasis of the tumor. However, based on the present data, more attention should be given to tumor progression in the context of metastasis and treatment, as it was clearly demonstrated that primary cells are genotypically and phenotypically different from metastatic cells. In the established MUG Lucifer cell lines, the lineages genetically match the respective parent tumor. Only Chr 1 presented a difference between primary and metastatic tissue and their corresponding cell lines, by presenting a gain in the metastatic tumor which covers the region of, protooncogene ABL2 and tyrosine kinase DDR2 (RTKs) protein families. RTKs play a key role in the communication, and are involved in cell adhesion, proliferation, and extracellular matrix remodeling [36].

The fact that genetic factors hardly play a role in CCS therapy moved the focus of investigation to the epigenetics level. Here, the aim was to gain a deeper understanding of this complex biological domain and in turn find innovative tailored treatments. DNA methylation analyses identified genes differentially methylated in both cell lines and facilitated possible tailor-made therapies. MUG Lucifer prim and MUG Lucifer met possessed distinct DNA methylation profiles, presenting among others a hypermethylation of RUNX3, which functions as a tumor suppressor [37] and of miR-886, which plays an important role in cell growth regulation [38].

However, finding adequate therapy options with DNA methylation was not successful. In order to understand the epigenetic differences in both cell lines, a more detailed analysis was conducted by measuring ArgMet levels with NMR spectroscopy [14]. MUG Lucifer prim and MUG Lucifer met showed elevated ADMA/ARG ratios compared to healthy fibroblasts indicating an increased PRMT Type I activity. However, (SDMA + MMA)/ARG ratios of MUG Lucifer prim and MUG Lucifer met cells were not increased in comparison with healthy fibroblasts, which indicates a normal or even decreased PRMT Type II activity. GSK 3368715, a small molecule PRMT Type I inhibitor with an already implemented phase I trial in 2018 (NCT03666988), could potentially be suitable for treating MUG Lucifer prim and MUG Lucifer met cells due to their elevated ADMA/ARG levels [39, 40]. This potent, reversible Type I PRMT inhibitor exerts anti-proliferative effects on various tumor cell lines in vitro and in vivo [41] and its safety, tolerability, and PK profile are currently under clinical investigation to determine its potential therapeutic benefit. GSK 591 is a PRMT5 (Type II) inhibitor [39, 40] that was used as a control.

To predict the efficacy of PRMT1 inhibitors in CCSs, a 3D culture model is used that mimics the microenvironment of a tissue, where cells are able to proliferate, aggregate and differentiate [42]. Furthermore, compared to 2D culture, 3D culture systems reflect cellular heterogeneity, cell–cell interactions and molecular divergence in a more physiological way [43]. The differences between 2D and 3D cell culture experiments is, that 3D cultures maintain normal morphology leading to a different organization of surface receptors on the cell. This is important for resembling the in vivo condition as drugs often target specific cell surface receptors. Furthermore, cells cultured in 2D are often in the same cell cycle stage, whereas 3D cells have different cell stages and thus better reflect the situation in vivo [46]. Furthermore, proliferating cells in 3D models are located in the outer region of the model in most cases, and many drugs require cell proliferation to be more effective [45].

ApoTox-Glo™ Triplex assays were conducted to determine viability, cytotoxicity, and late apoptosis of PRMT inhibitor treated MUG Lucifer cells simultaneously. While the viability of MUG Lucifer prim cells decreases after GSK 3368715 treatment, cytotoxicity and apoptosis increase significantly confirming the apoptotic effect of GSK 3368715 on MUG Lucifer prim cells. The high cytotoxicity levels could be explained by the unfavorable incubation time of six days, as cytostatic effects should not increase cytotoxicity levels [9, 47]. Viability, cytotoxicity, and apoptosis, however, did not change at all in MUG Lucifer met cells after AdOx treatment. MUG Lucifer met cells exhibit a high caspase 3/7-activity even without PRMT inhibitor treatment. Liu et al*.* already described caspase activities regarding tumorigenesis as a two-sided sword; low caspase levels are associated with cancer and treatment resistance, while caspase overexpression in apoptotic cancer cells has a growth stimulating effect on the non-apoptotic tumor cells making an increased cell survival possible [48].

To mimic CCS as closely as possible and to avoid animal experiments, autologous 3D models were conducted. Since MUG Lucifer prim but also MUG Lucifer met do not form completely compact, solid spheroids, co-cultivation with MUG Lucifer hTERT fibroblasts was performed. Interestingly, compared to 2D experiments, GSK 3368715 showed an effect on both MUG Lucifer prim and MUG Lucifer met spheroids. These results indicate that GSK 3368715 inhibitor does induce growth inhibition in both types of CCS cells and proves that 3D cell culture is preferable to 2D cell culture, as it more accurately represents the in vivo situation regarding for instance gene expression and drug uptake [49, 50]. Moreover, the apoptosis determination of GSK 3368715 treated spheroids showed significantly elevated levels of caspase activity in MUG Lucifer prim cells and their co-culture with MUG Lucifer hTERT fibroblasts in comparison to the MUG Lucifer hTERT fibroblast single culture, which demonstrates that GSK 3368715 does induce apoptosis in cancer cells, but not in healthy fibroblasts. The caspase activities in MUG Lucifer met cells and their co-culture with MUG Lucifer hTERT fibroblasts, do not show significant changes compared to MUG Lucifer hTERT fibroblasts alone, which further confirms the results of the 2D experiments. This renders GSK 3368715 a suitable novel treatment option for CCS patients exhibiting elevated ADMA/ARG levels as manifested in our autologous tumor model.

## Conclusions

The established cell lines MUG Lucifer prim and MUG Lucifer met form a suitable CCS model that reliably reflects the in vivo process and recapitulates aspects of clinical multistep carcinogenesis. Based on the epigenetic data, a PRMT Type I inhibitor was used to demonstrate the targeted antitumor effect, with no effect on the respective fibroblasts isolated from the same patient. Based on these data and the promising effects observed in the present study, tailored therapies should be considered, especially in the advanced metastatic stage of CCS.

## Supplementary Information

**Additional file 1: Figure S1.** A transmission electron micrograph montage of MUG Lucifer cells. Cells show a cluster of mitochondria (m) and a strong folded surface with a high amount of elongated extensions (asterisk), scale bar represents 5 µm. **Figure S2.** Spheroids (2500 cells) of MUG Lucifer prim (A), MUG Lucifer met (B), MUG Lucifer hTERT fibroblasts (C), MUG Lucifer hTERT fibroblasts GFP (D), MUG Lucifer prim + MUG Lucifer hTERT fibroblasts (1 + 1; E), MUG Lucifer prim + MUG Lucifer hTERT fibroblasts (1 + 1, GFP; F), MUG Lucifer met + MUG Lucifer hTERT fibroblasts (1 + 1; G) and MUG Lucifer met + MUG Lucifer hTERT fibroblasts (1 + 1, GFP; H) in 100 × magnification. Scale bar represents 100 µm. Figure S3: Pilot in vivo xenograft experiment of MUG Lucifer cell lines: plot of tumor increase in volume correlated to time of both cell lines (A); macroscopical visualization of the excised tumors (B). **Figure S4**. HE, MelanA, HMB45 and SOX10 staining of MUG Lucifer prim and MUG Lucifer met xenografts. **Figure S5**. Location of DMPs with respect to CpG Island and gene annotations. **Figure S6**. Cell cycle histograms of MUG Lucifer prim and MUG Lucifer met spiked with PBMNCs. **Figure S7-42**. Locations on chromosomes and in relation to genes and CpG islands of 36 regions (DMRs) which are differentially methylated (p < 0.01). Probe-level methylation data is shown as heatmaps and lineplots.

**Additional file 2: Table S1**. STR profiles of MUG Lucifer prim tumor tissue, tissue from the patient’s skin, MUG Lucifer prim passages p17 and p70 and MUG Lucifer met passages p5 and p62. **Table S2.** Copy number profiles of MUG Lucifer cell lines. **Table S3**. Number of DMPs with respect to CpG Island locations and gene annotations.

## Data Availability

The data sets used and/or analyzed during the current study are available from the corresponding authors on reasonable request.

## Abbreviations

ADMA: Asymmetric dimethylarginine
AdOx: Adenosine periodate oxidized
ArgMet: Arginine methylation
ARG: Arginine
CCS: Clear cell sarcoma
hTERT: Human telomerase reverse transciptase
MMA: Monomethylarginine
NMR: Nuclear magnet resonance
PBMNCs: Peripheal blood mononuclear cells
PRMT: Protein arginine methyltransferases
SACF: Soft agar colony formation assay
SAH: s-adenosyl-l-homocysteine
SDMA: Symmetric dimethylarginine
STR: Short tandem repeats
TKI: Tyrosine kinase inhibitor
