# Supplementary material for: Targeting epigenetic features in clear cell sarcomas based on patient-derived cell lines
Source: J Transl Med. 2023 Jan 29;21:54. doi: 10.1186/s12967-022-03843-4 (PMC9884415; doi:10.1186/s12967-022-03843-4)
Supplement: Supplementary file 1 — Additional file 1: Figure S1. A transmission electron micrograph montage of MUG Lucifer cells. Cells show a cluster of mitochondria (m) and a strong folded surface with a high amount of elongated extensions (asterisk), scale bar represents 5 µm. Figure S2. Spheroids (2500 cells) of MUG Lucifer prim (A), MUG Lucifer met (B), MUG Lucifer hTERT fibroblasts (C), MUG Lucifer hTERT fibroblasts GFP (D), MUG Lucifer prim + MUG Lucifer hTERT fibroblasts (1 + 1; E), MUG Lucifer prim + MUG Lucifer hTERT fibroblasts (1 + 1, GFP; F), MUG Lucifer met + MUG Lucifer hTERT fibroblasts (1 + 1; G) and MUG Lucifer met + MUG Lucifer hTERT fibroblasts (1 + 1, GFP; H) in 100 × magnification. Scale bar represents 100 µm. Figure S3: Pilot in vivo xenograft experiment of MUG Lucifer cell lines: plot of tumor increase in volume correlated to time of both cell lines (A); macroscopical visualization of the excised tumors (B). Figure S4. HE, MelanA, HMB45 and SOX10 staining of MUG Lucifer prim and MUG Lucifer met xenografts. Figure S5. Location of DMPs with respect to CpG Island and gene annotations. Figure S6. Cell cycle histograms of MUG Lucifer prim and MUG Lucifer met spiked with PBMNCs. Figure S7-42. Locations on chromosomes and in relation to genes and CpG islands of 36 regions (DMRs) which are differentially methylated (p < 0.01). Probe-level methylation data is shown as heatmaps and lineplots. [file 12967_2022_3843_MOESM1_ESM.pptx]

## Slide 1
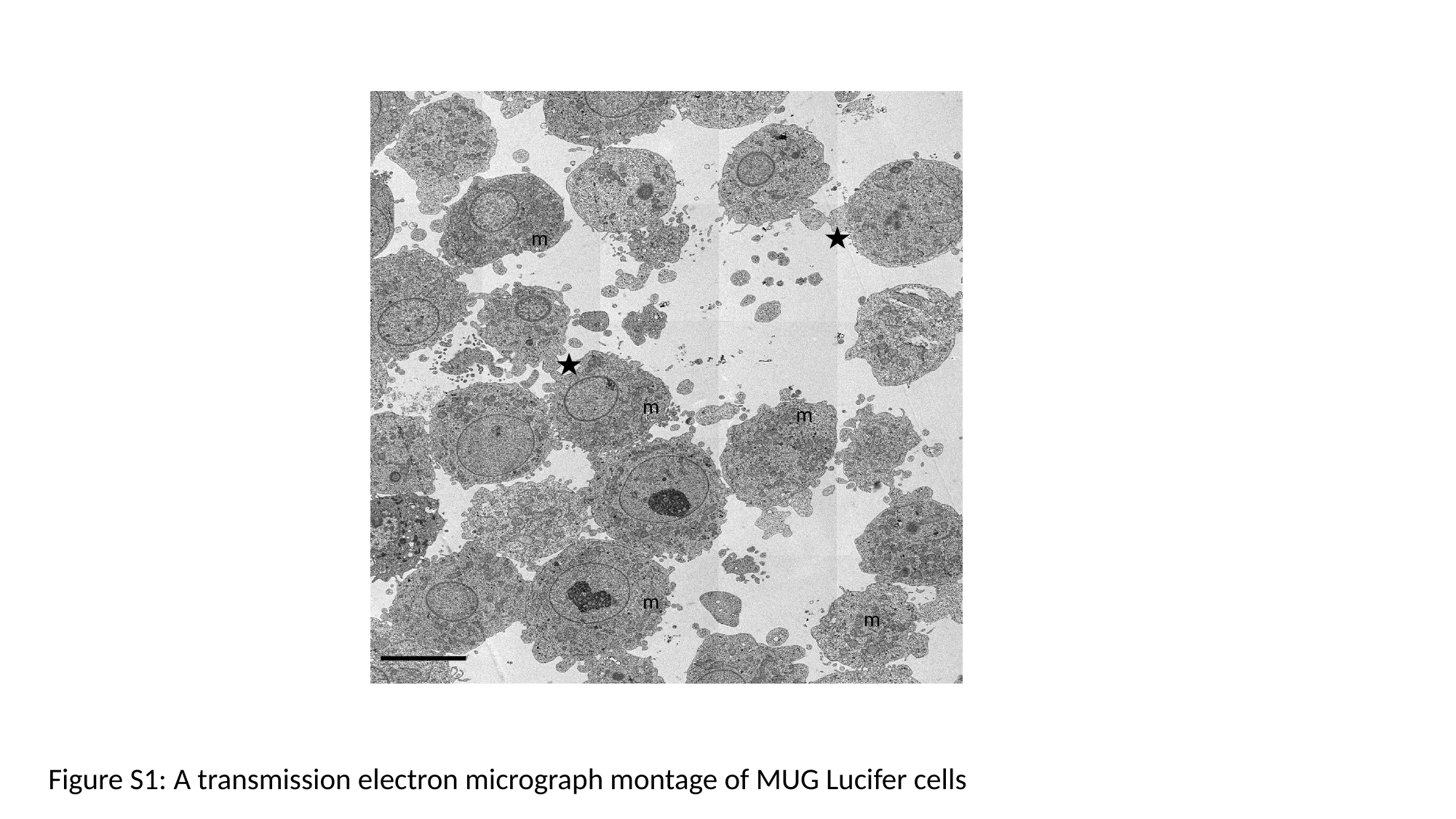

m
m
m
m
m
Figure S1: A transmission electron micrograph montage of MUG Lucifer cells

## Slide 2
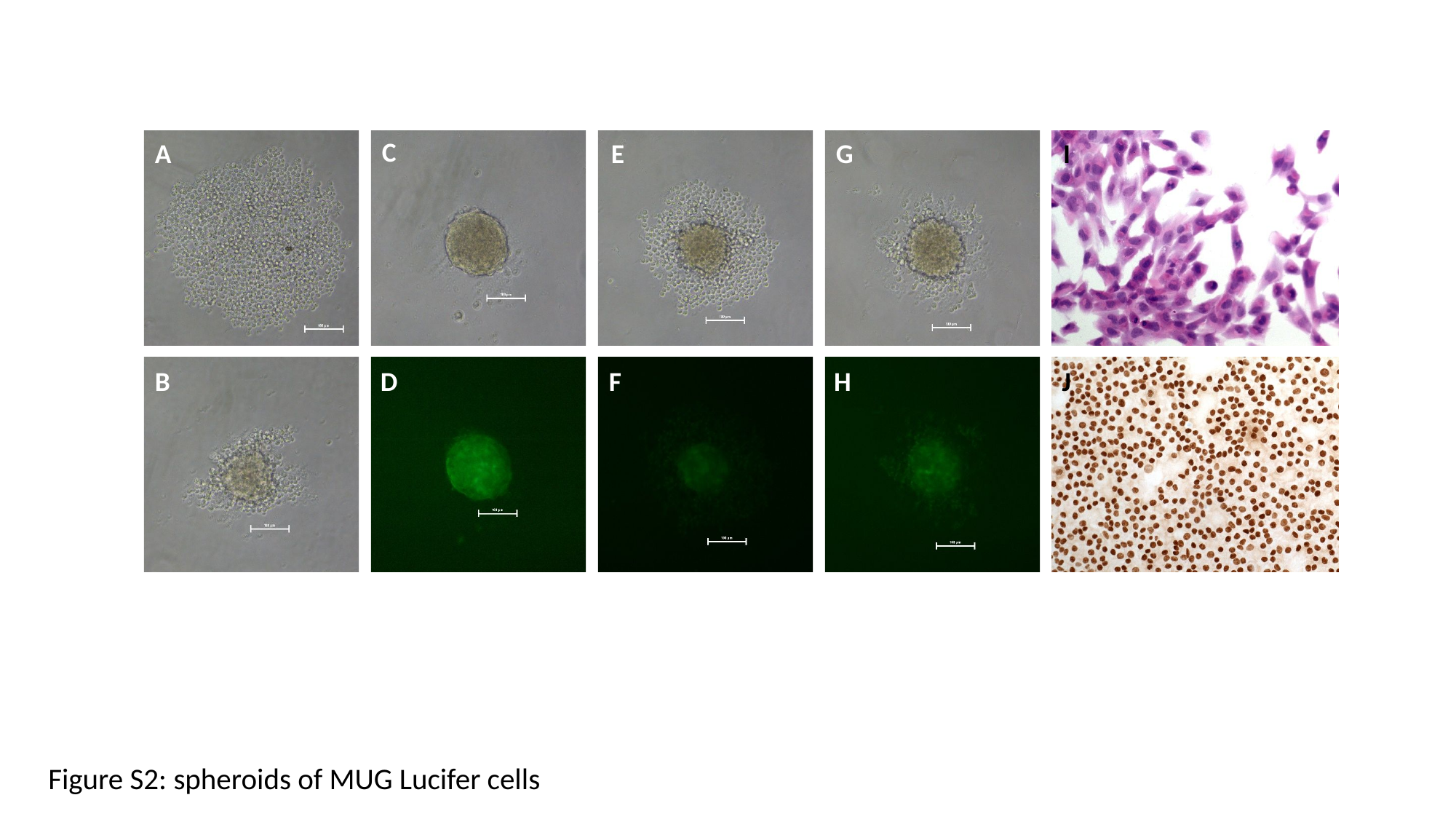

C
A
I
E
G
B
D
F
H
J
Figure S2: spheroids of MUG Lucifer cells

## Slide 3
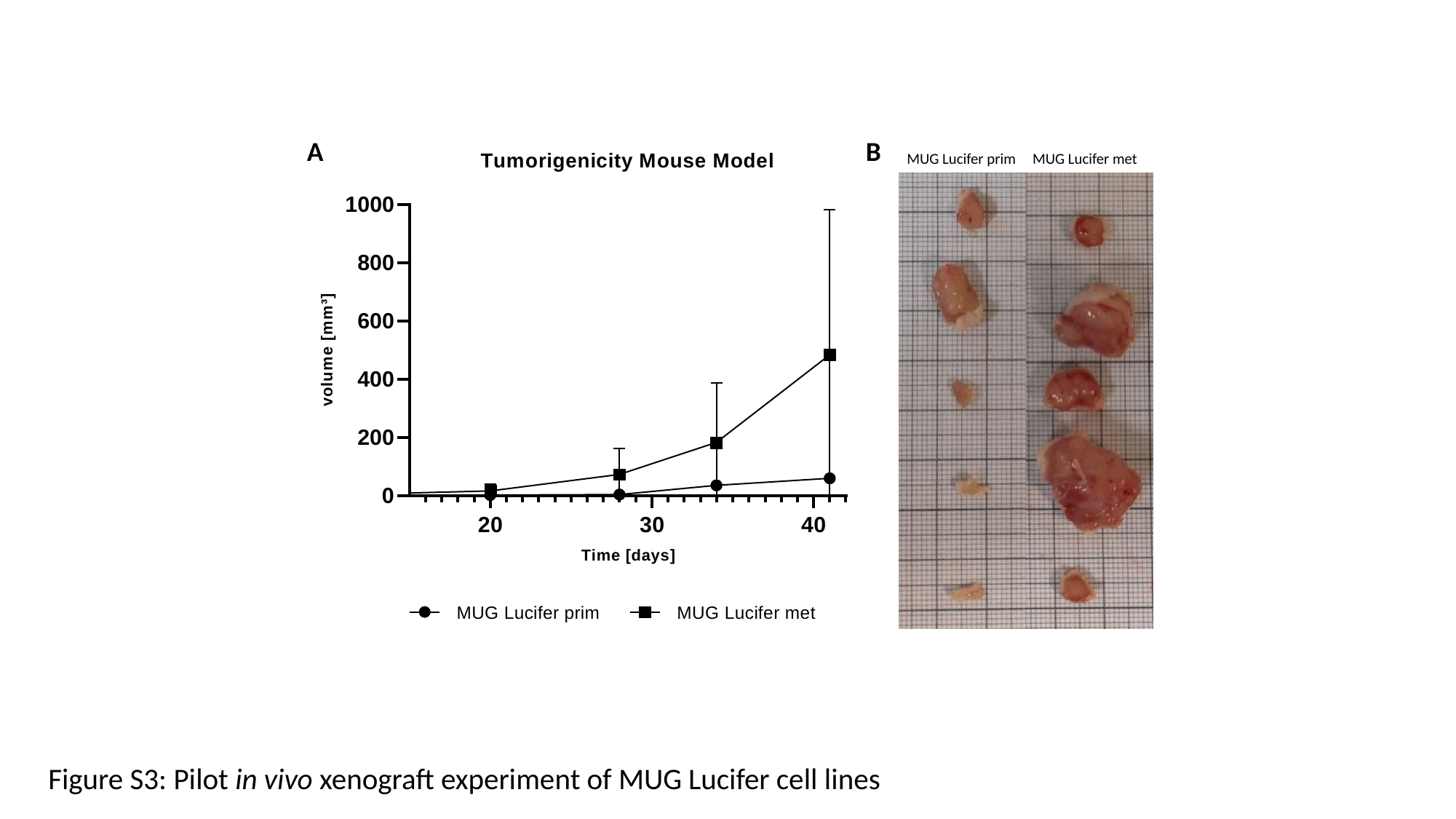

A
B
MUG Lucifer met
MUG Lucifer prim
Figure S3: Pilot in vivo xenograft experiment of MUG Lucifer cell lines

## Slide 4
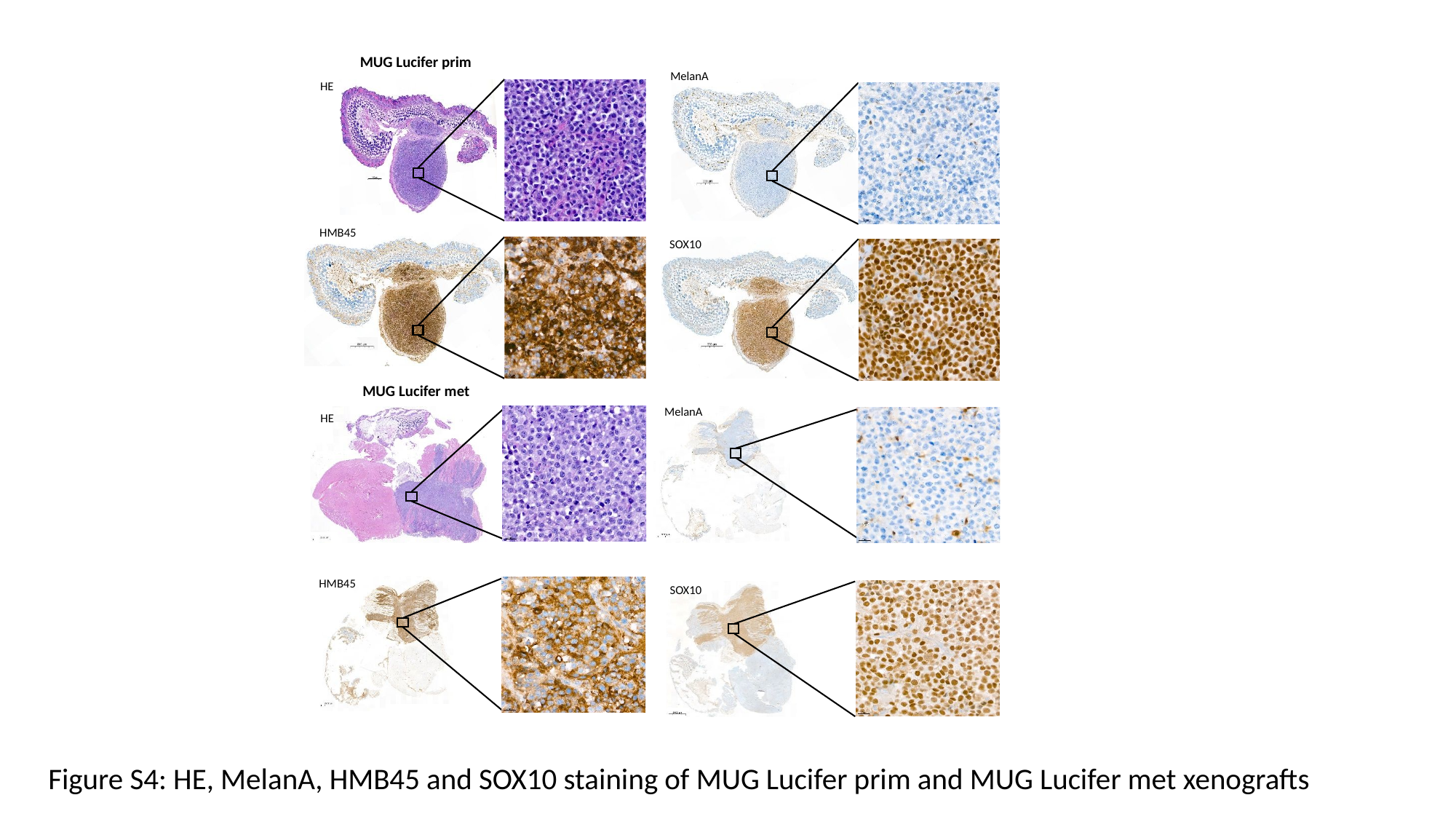

MUG Lucifer prim
MelanA
HE
HMB45
SOX10
MUG Lucifer met
MelanA
HE
HMB45
SOX10
Figure S4: HE, MelanA, HMB45 and SOX10 staining of MUG Lucifer prim and MUG Lucifer met xenografts

## Slide 5
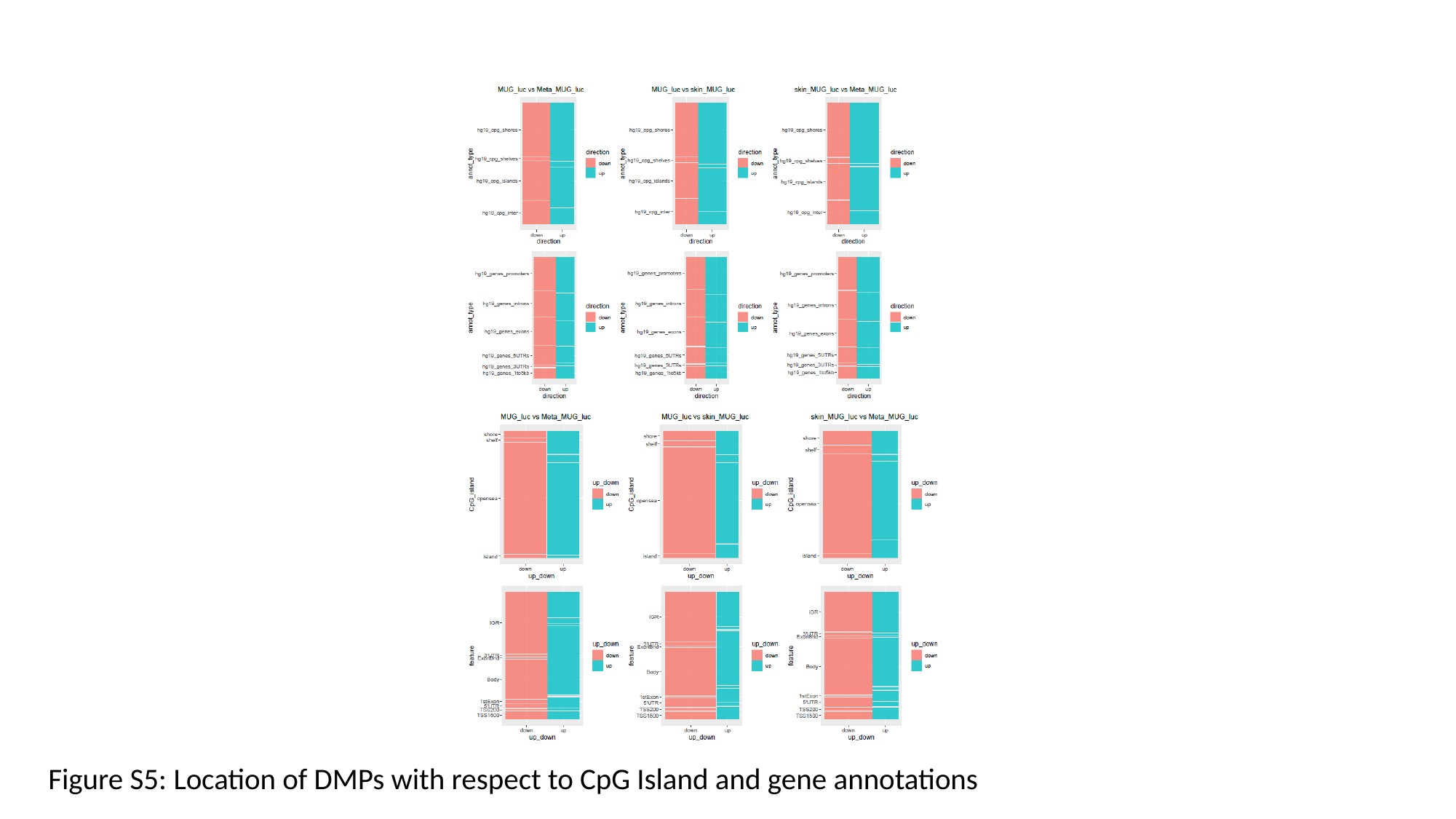

Figure S5: Location of DMPs with respect to CpG Island and gene annotations

## Slide 6
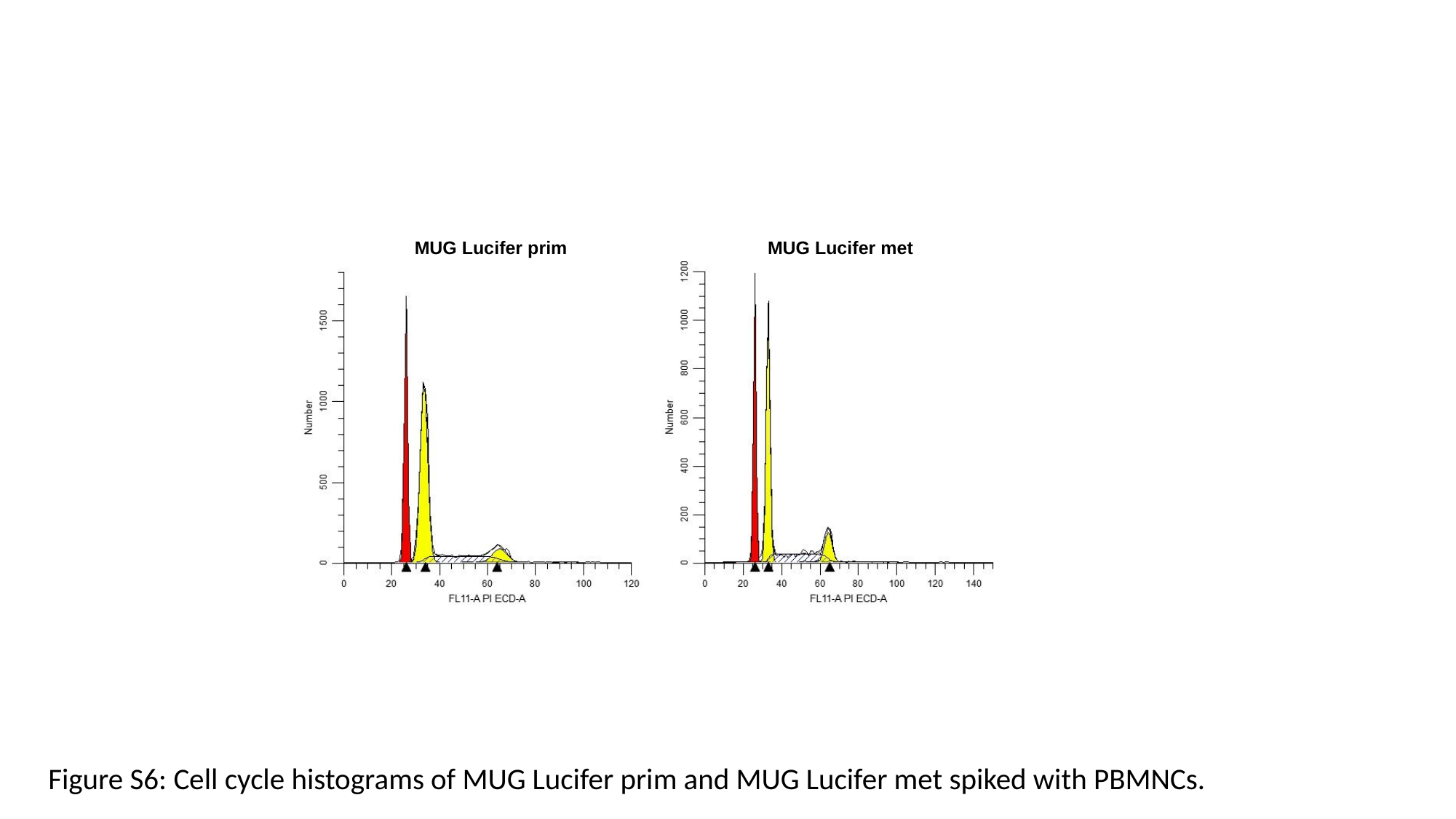

MUG Lucifer met
MUG Lucifer prim
Figure S6: Cell cycle histograms of MUG Lucifer prim and MUG Lucifer met spiked with PBMNCs.

## Slide 7
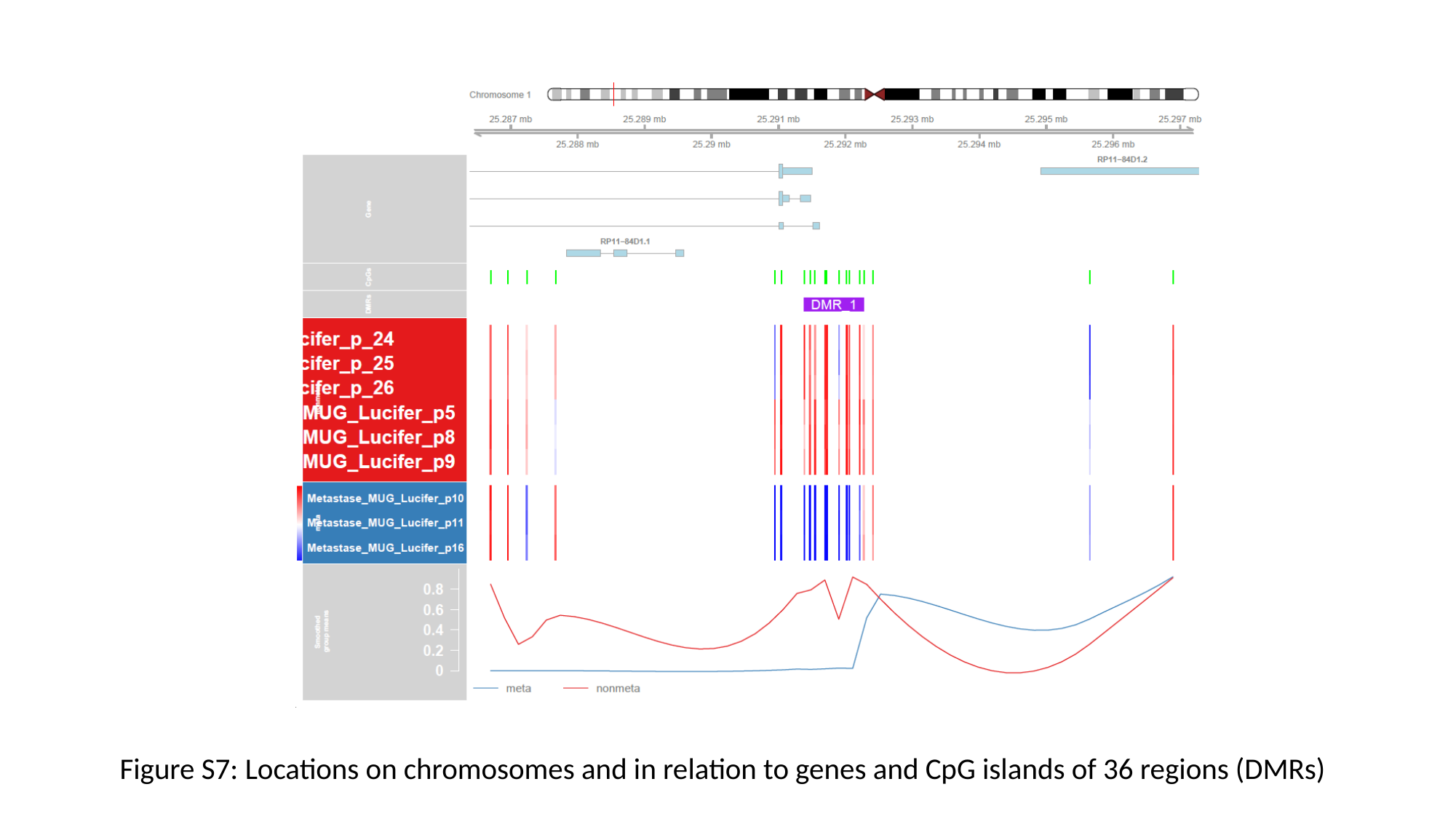

Figure S7: Locations on chromosomes and in relation to genes and CpG islands of 36 regions (DMRs)

## Slide 8
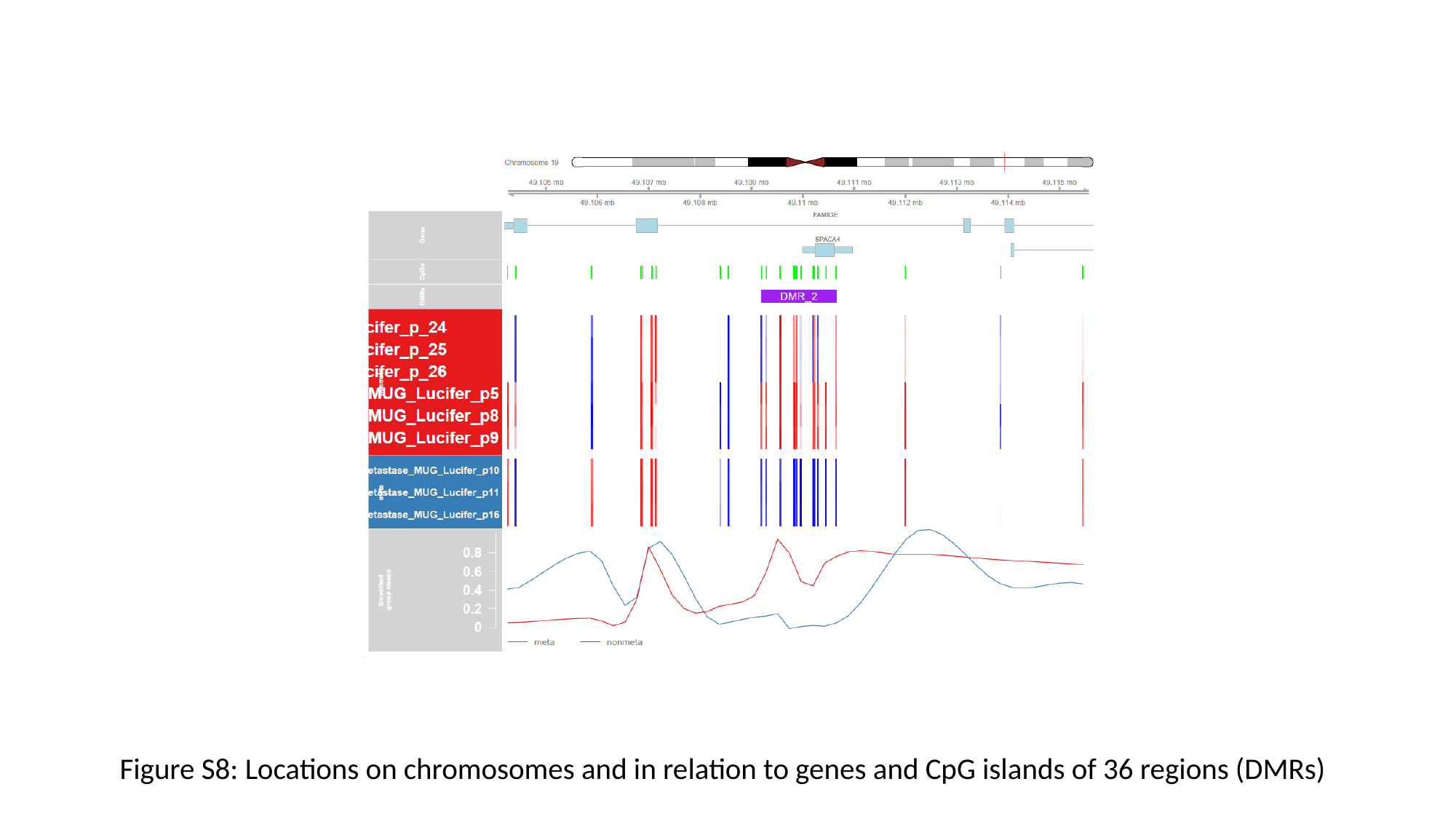

#
Figure S8: Locations on chromosomes and in relation to genes and CpG islands of 36 regions (DMRs)

## Slide 9
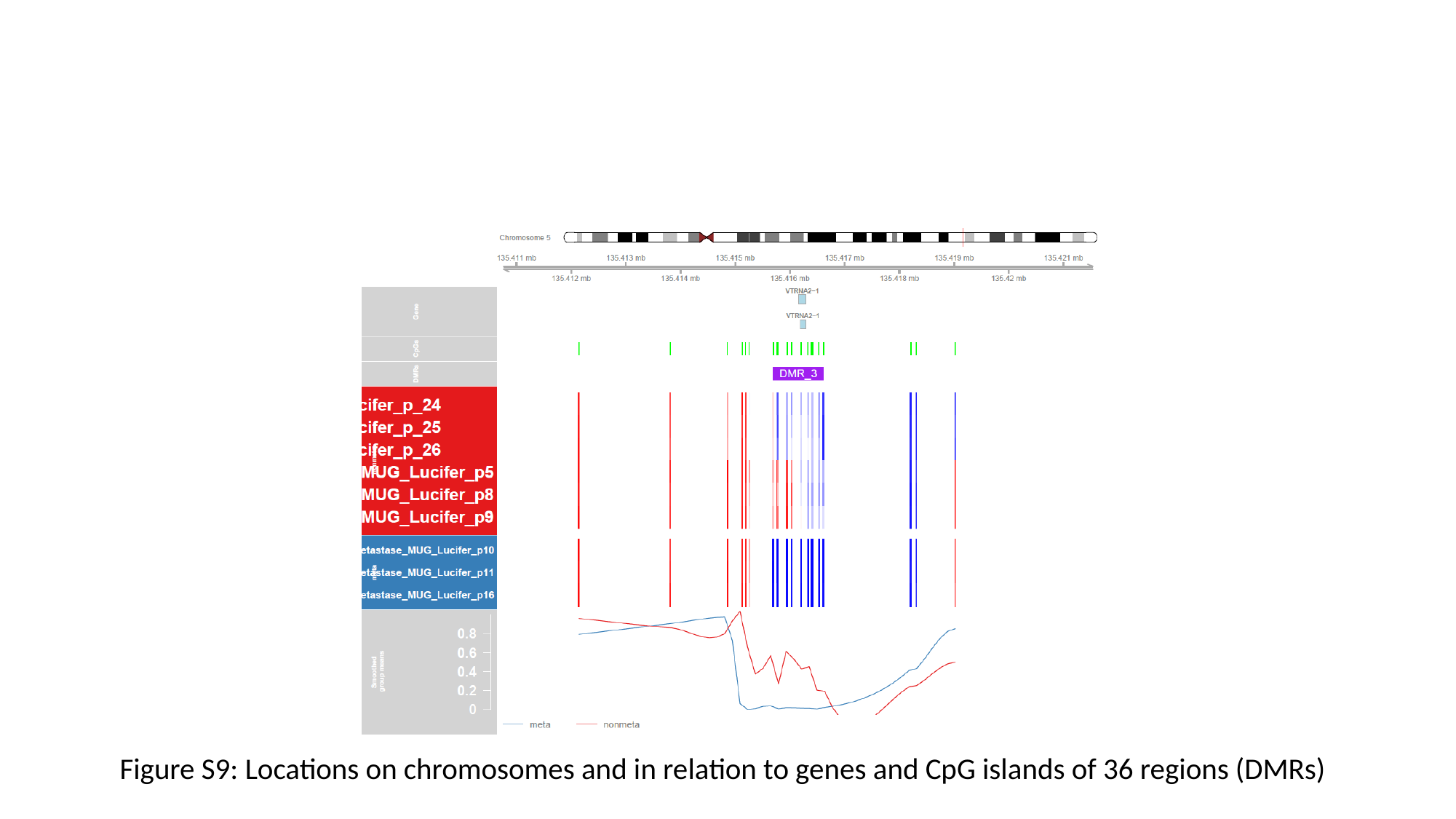

#
Figure S9: Locations on chromosomes and in relation to genes and CpG islands of 36 regions (DMRs)

## Slide 10
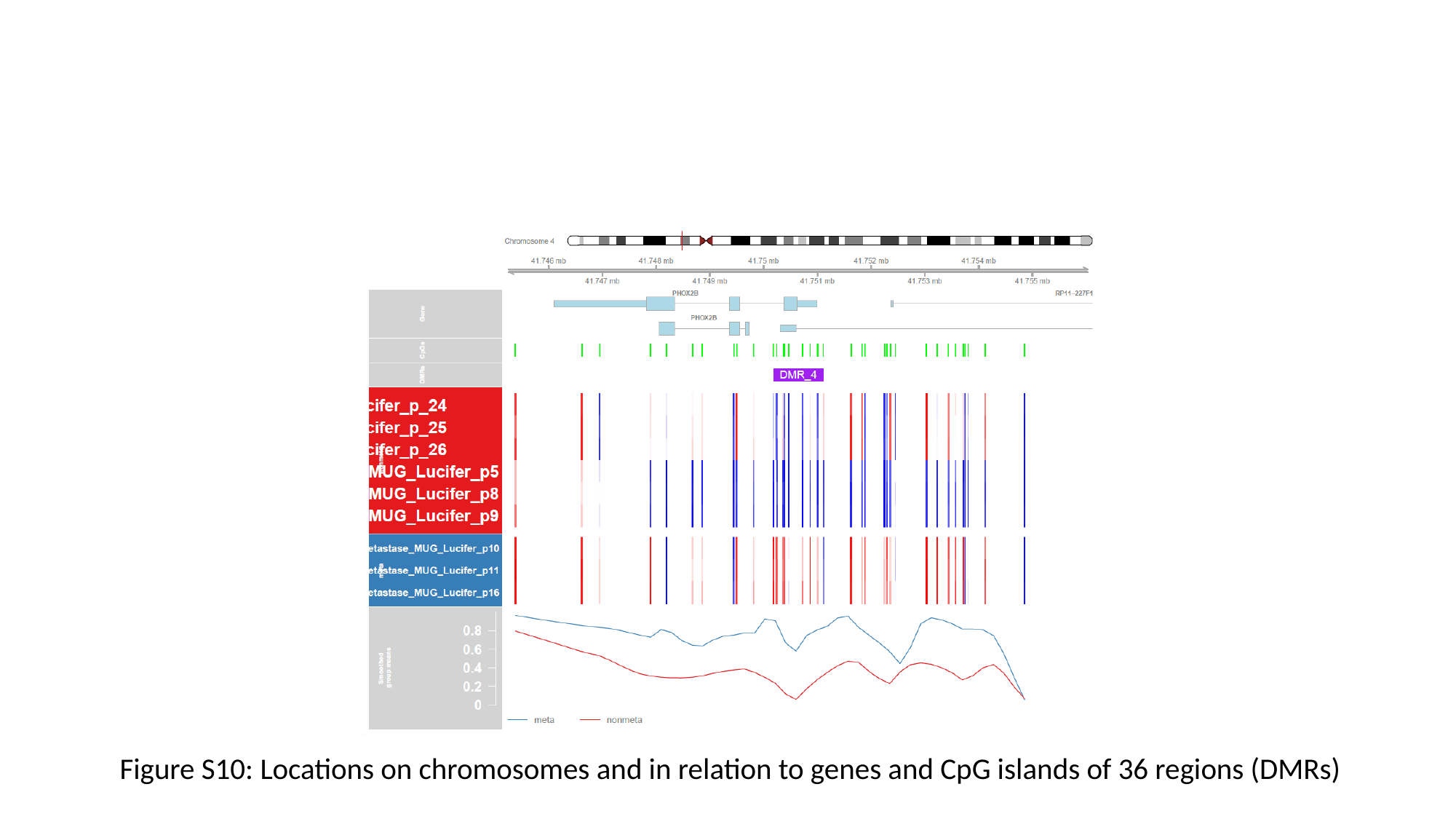

#
Figure S10: Locations on chromosomes and in relation to genes and CpG islands of 36 regions (DMRs)

## Slide 11
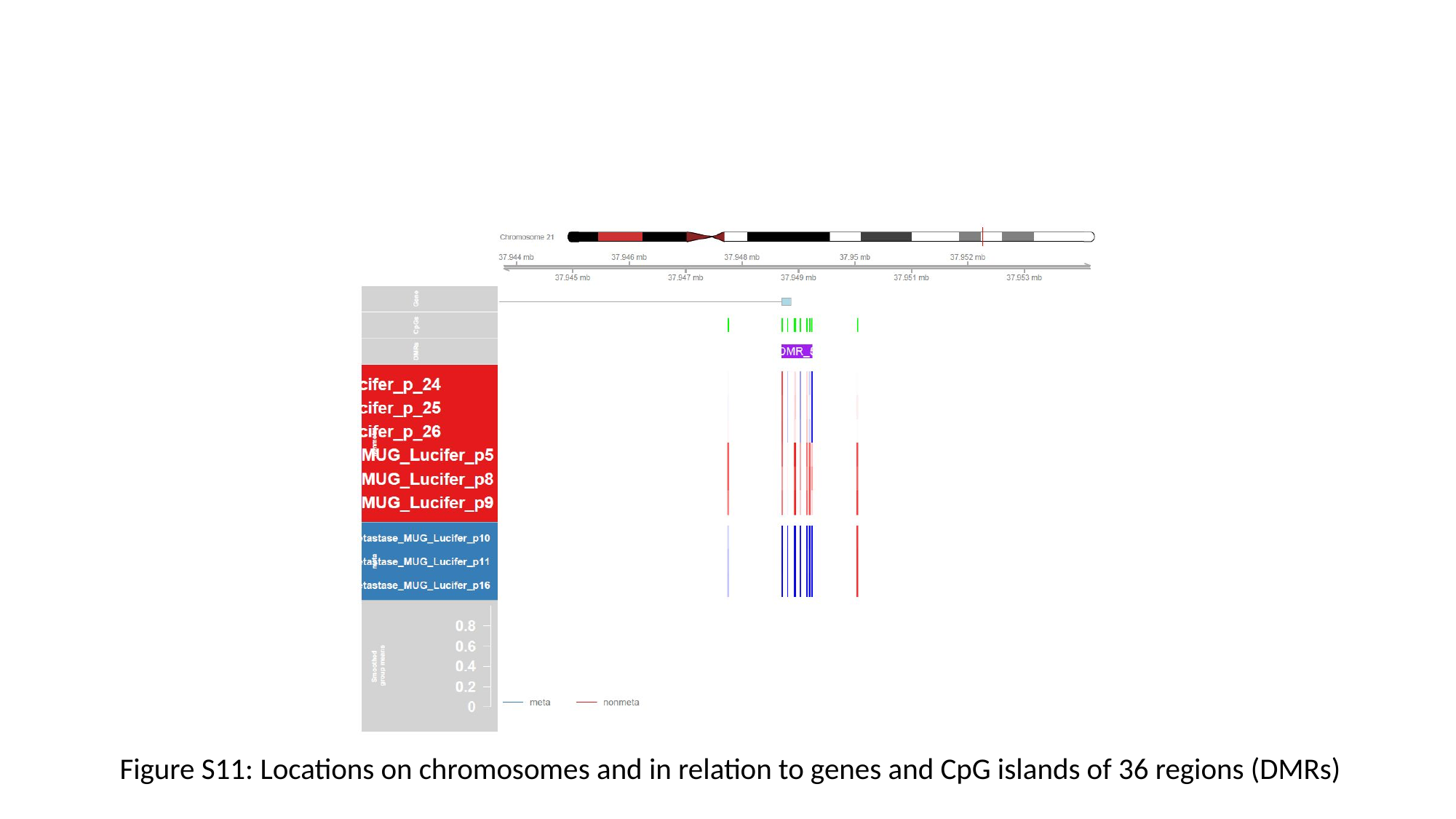

#
Figure S11: Locations on chromosomes and in relation to genes and CpG islands of 36 regions (DMRs)

## Slide 12
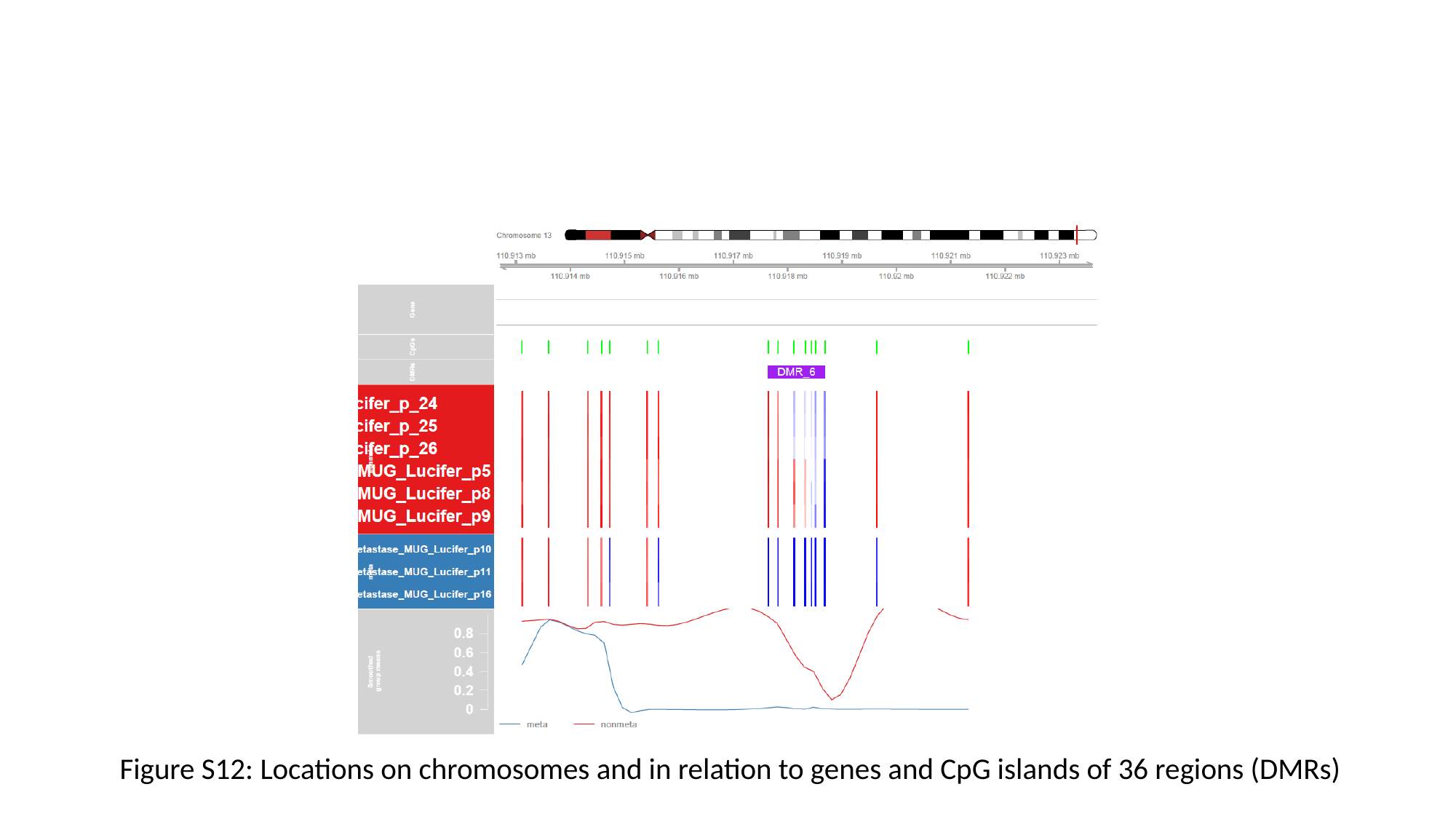

#
Figure S12: Locations on chromosomes and in relation to genes and CpG islands of 36 regions (DMRs)

## Slide 13
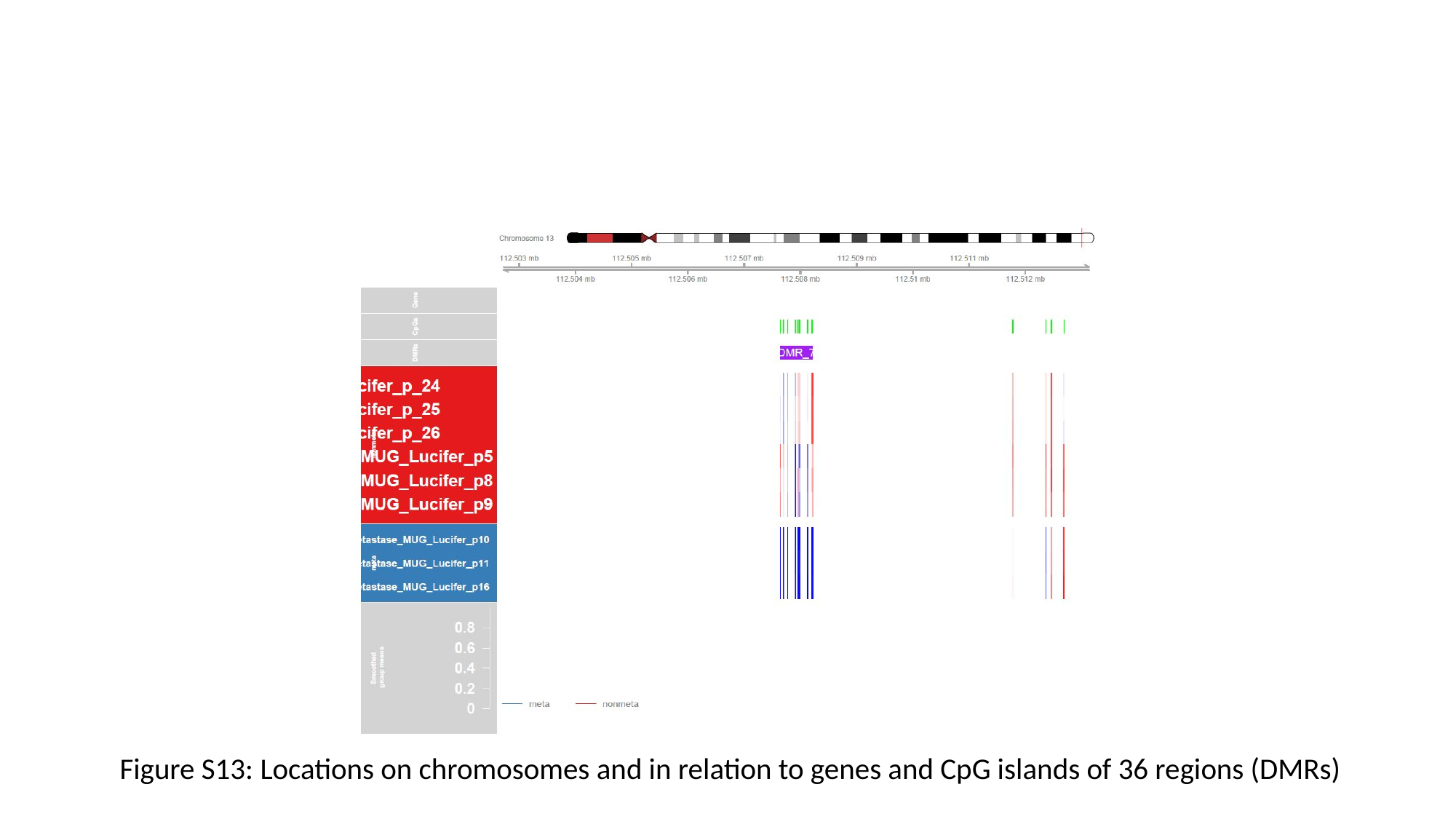

#
Figure S13: Locations on chromosomes and in relation to genes and CpG islands of 36 regions (DMRs)

## Slide 14
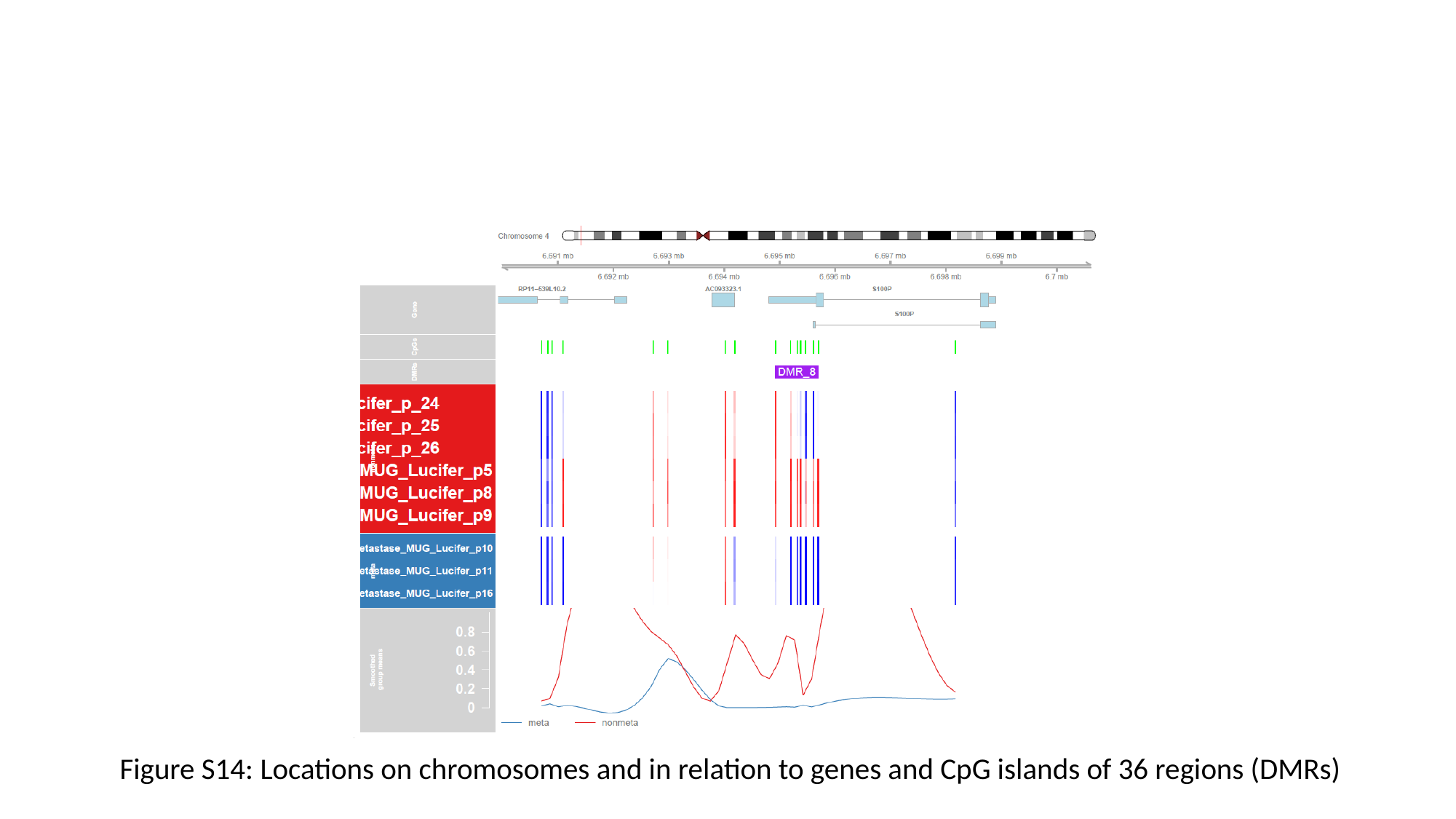

#
Figure S14: Locations on chromosomes and in relation to genes and CpG islands of 36 regions (DMRs)

## Slide 15
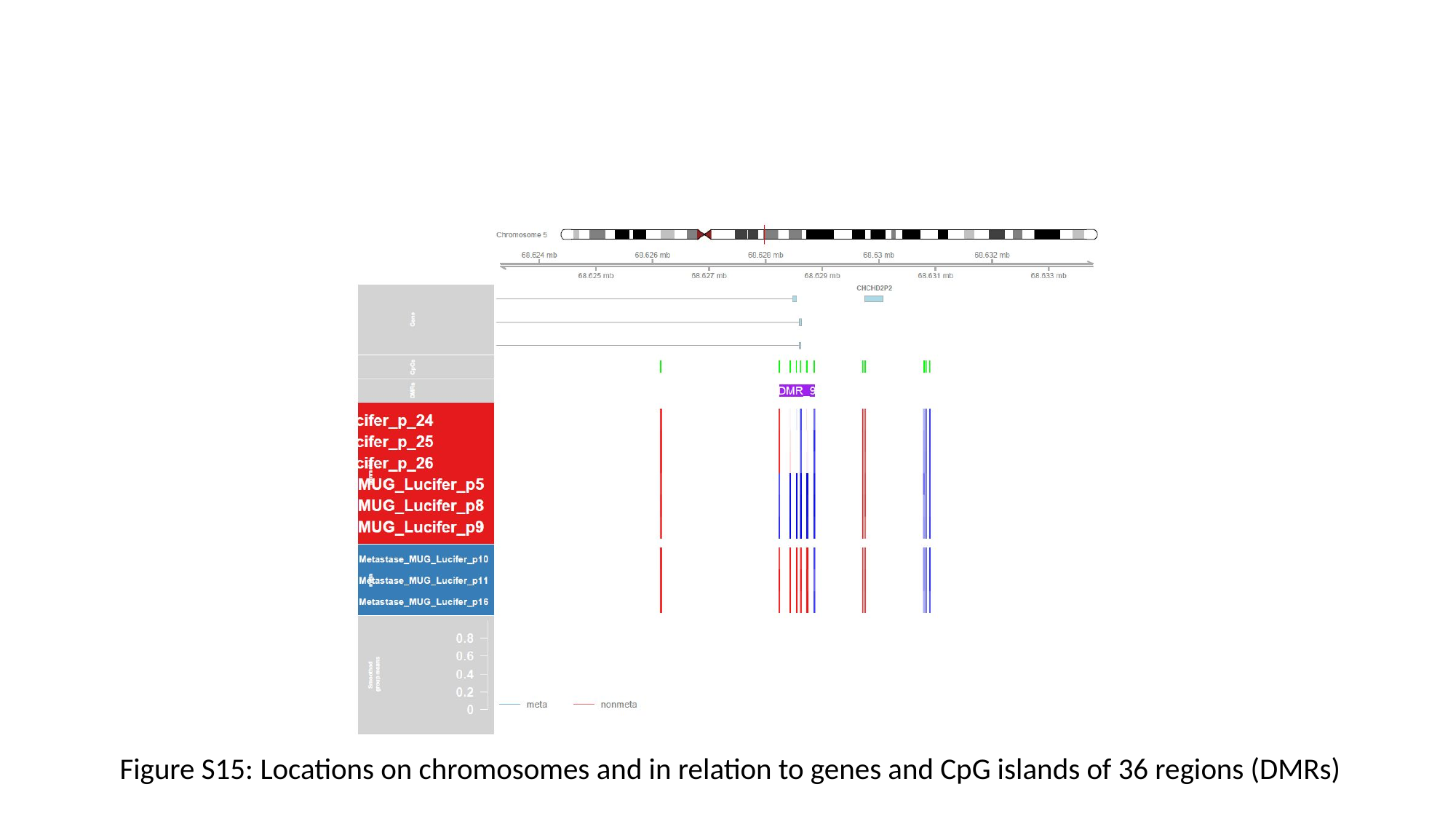

#
Figure S15: Locations on chromosomes and in relation to genes and CpG islands of 36 regions (DMRs)

## Slide 16
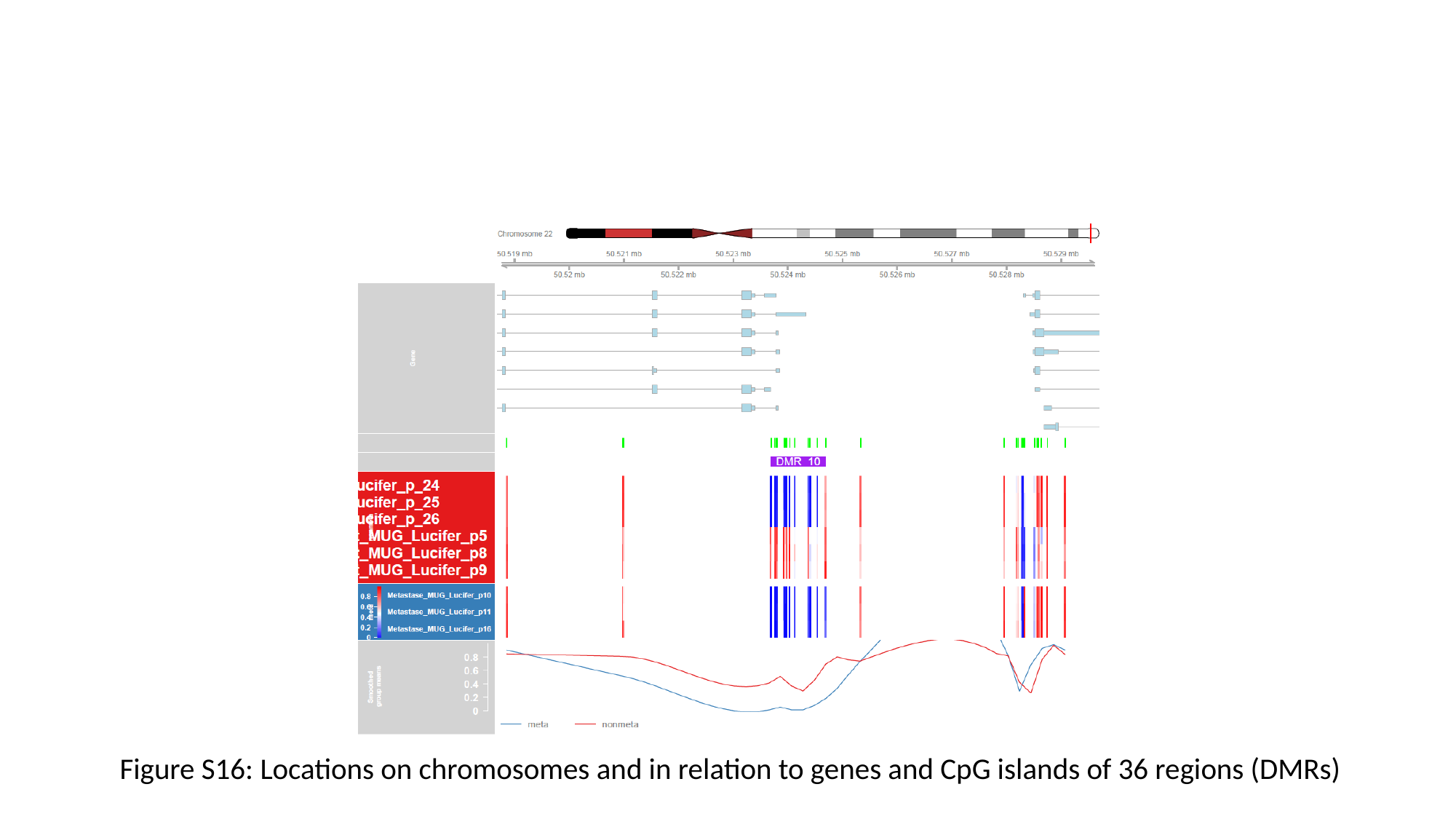

#
Figure S16: Locations on chromosomes and in relation to genes and CpG islands of 36 regions (DMRs)

## Slide 17
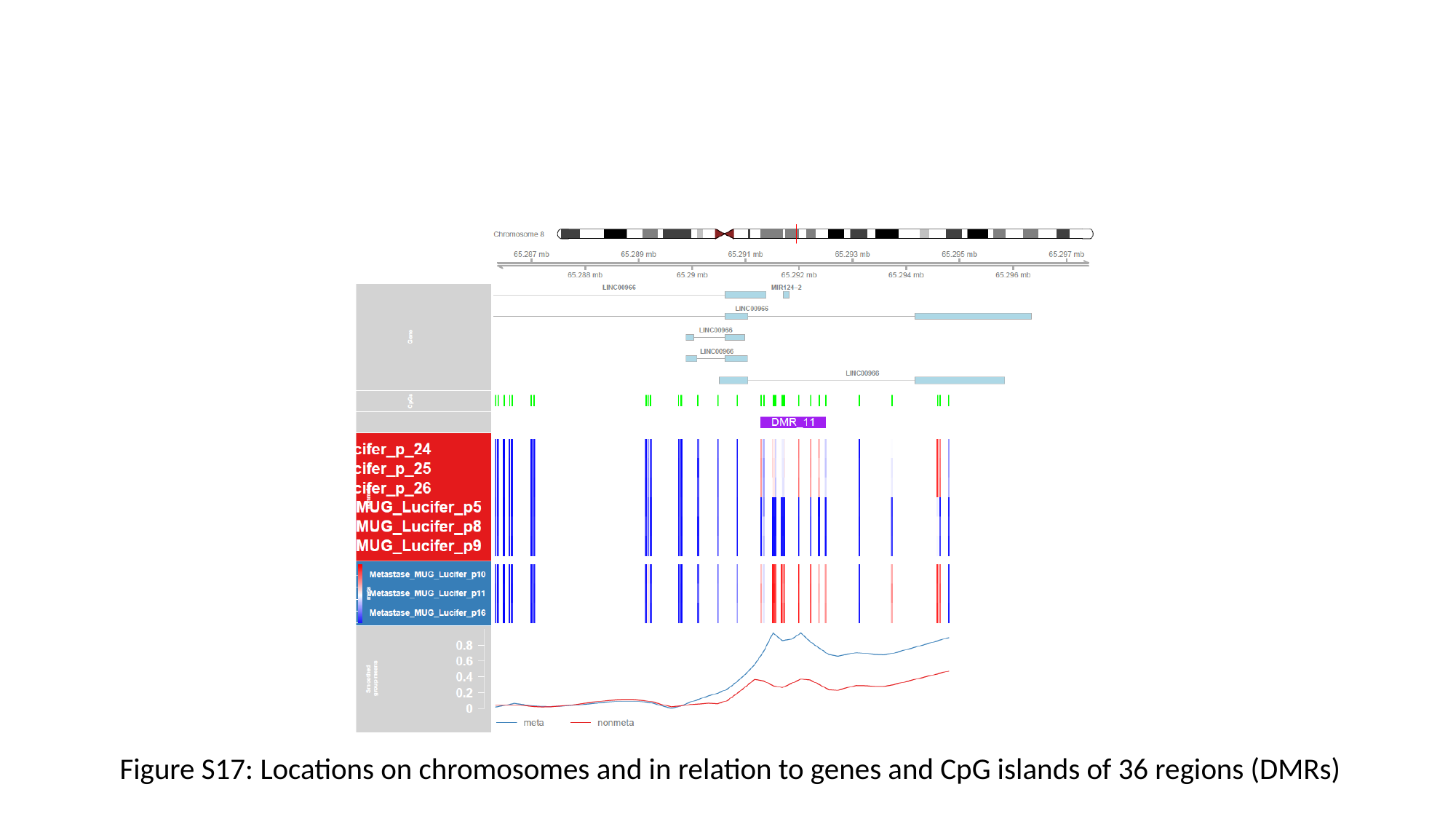

#
Figure S17: Locations on chromosomes and in relation to genes and CpG islands of 36 regions (DMRs)

## Slide 18
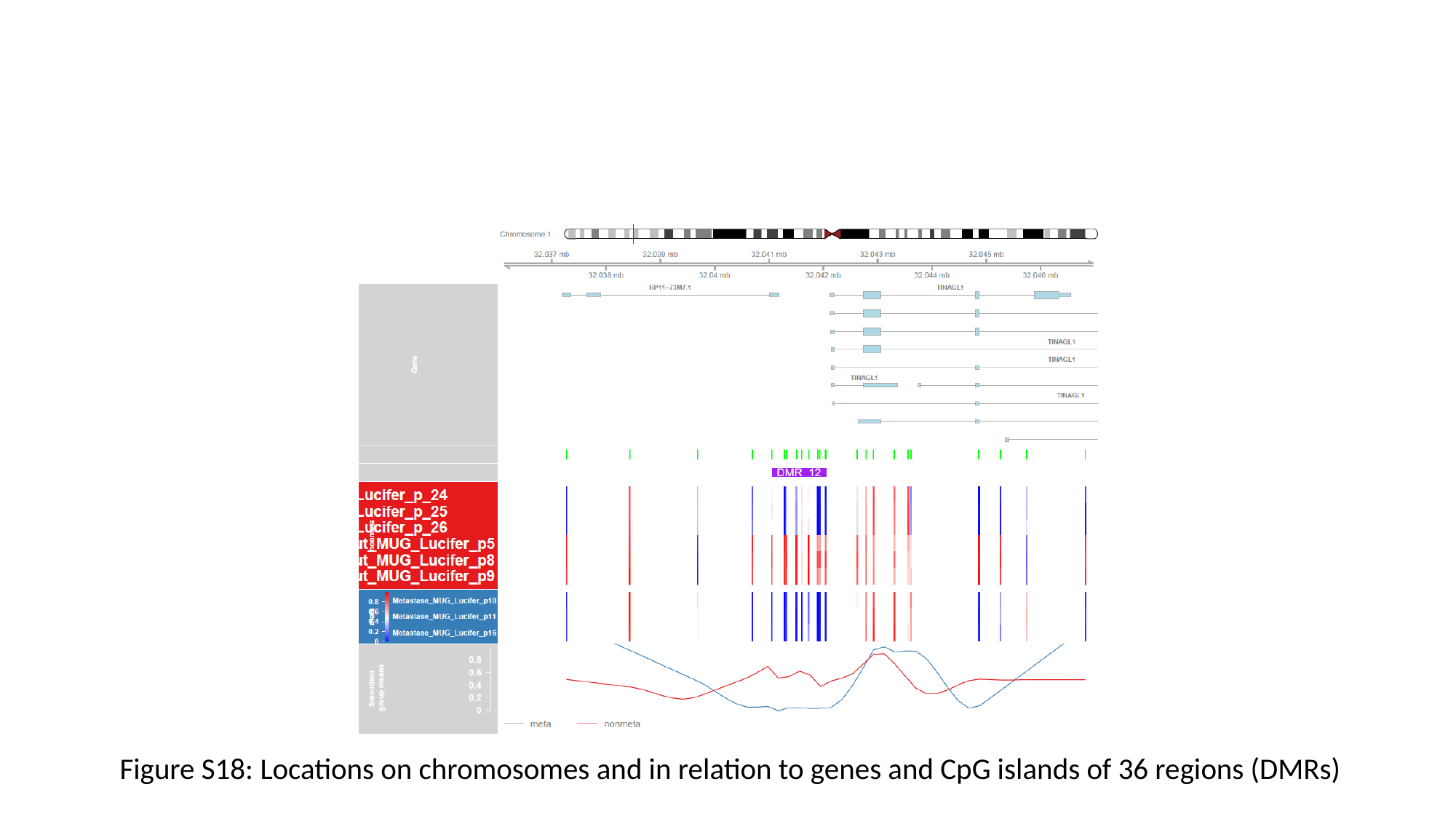

#
Figure S18: Locations on chromosomes and in relation to genes and CpG islands of 36 regions (DMRs)

## Slide 19
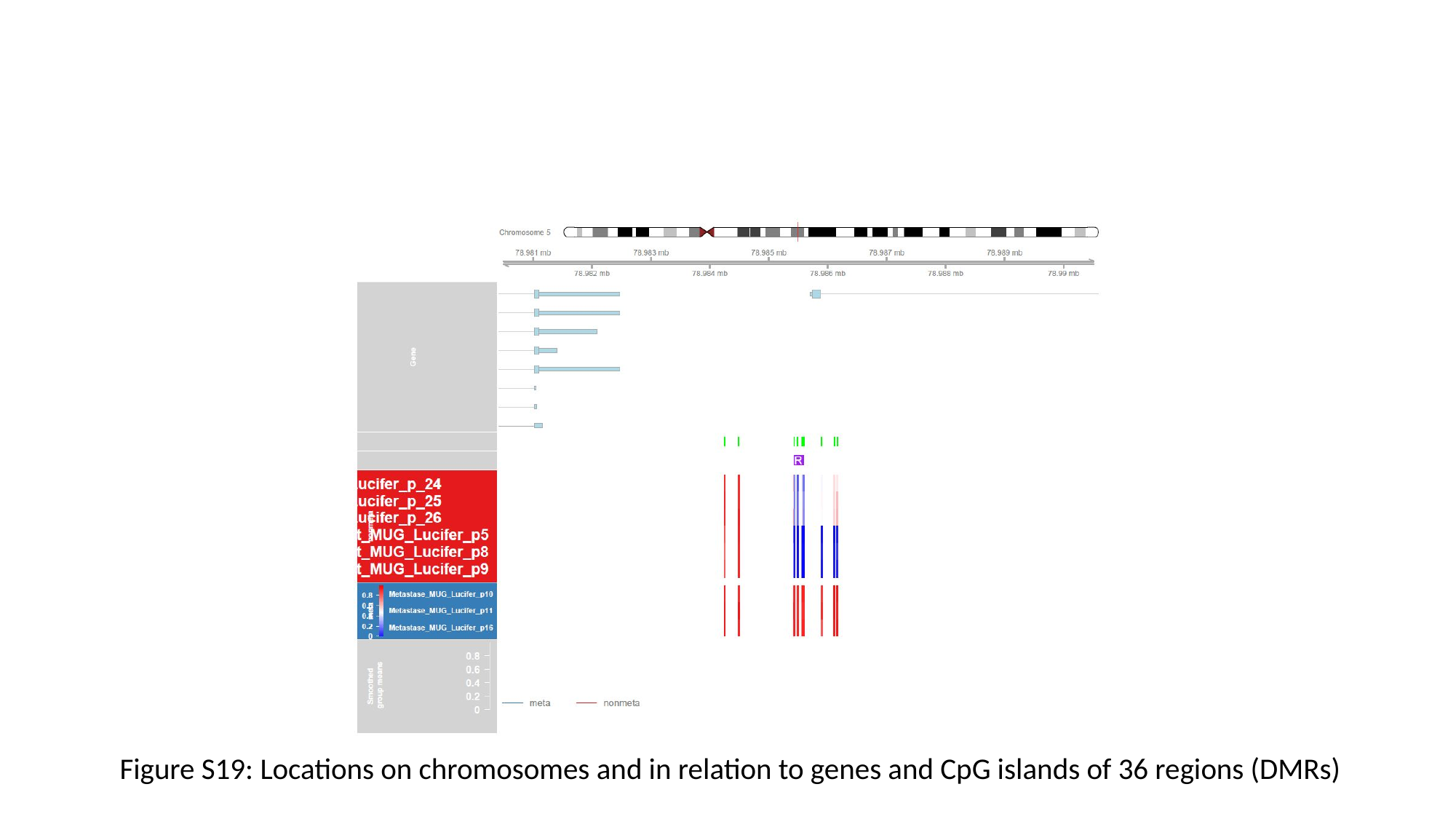

#
Figure S19: Locations on chromosomes and in relation to genes and CpG islands of 36 regions (DMRs)

## Slide 20
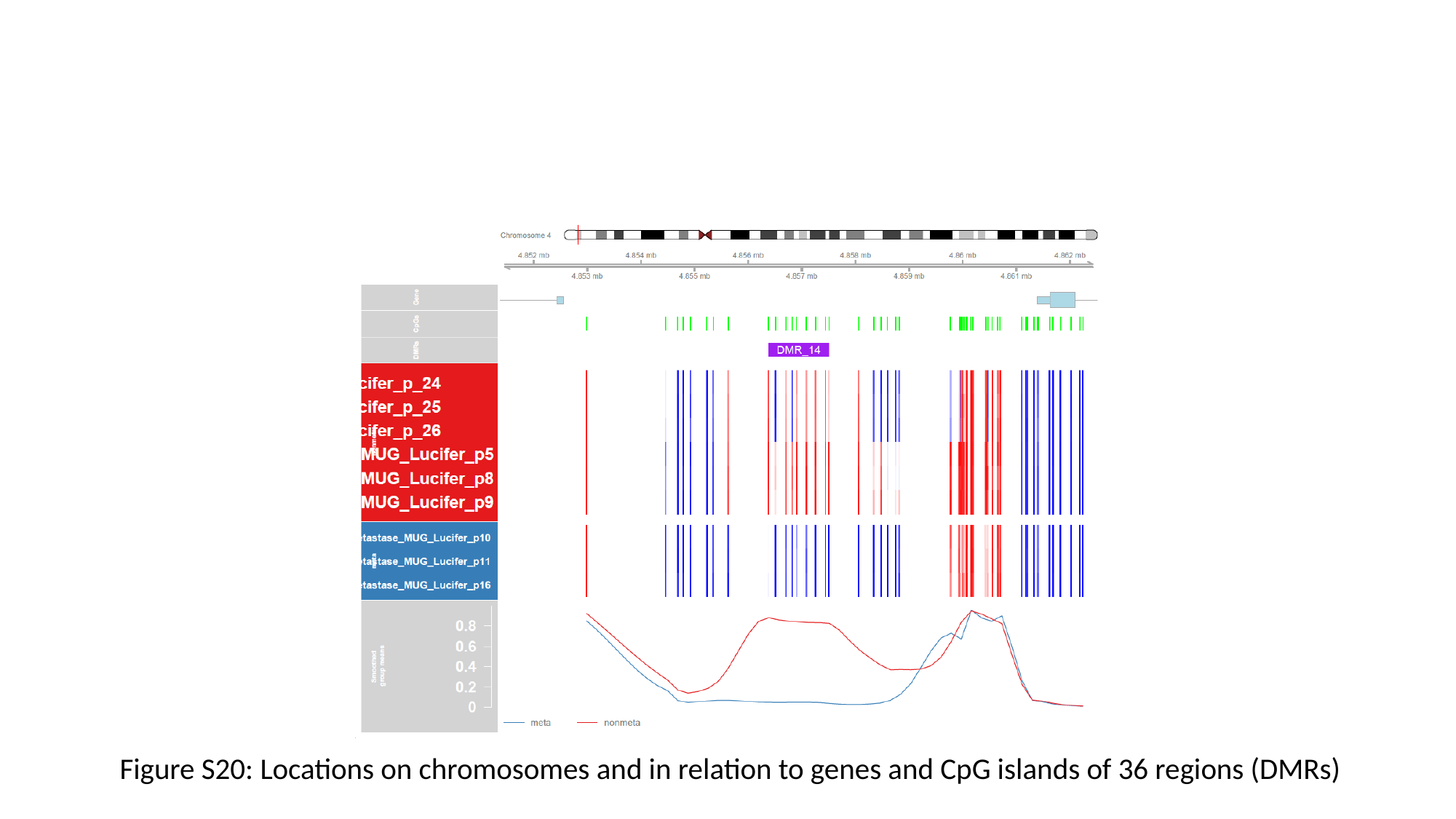

#
Figure S20: Locations on chromosomes and in relation to genes and CpG islands of 36 regions (DMRs)

## Slide 21
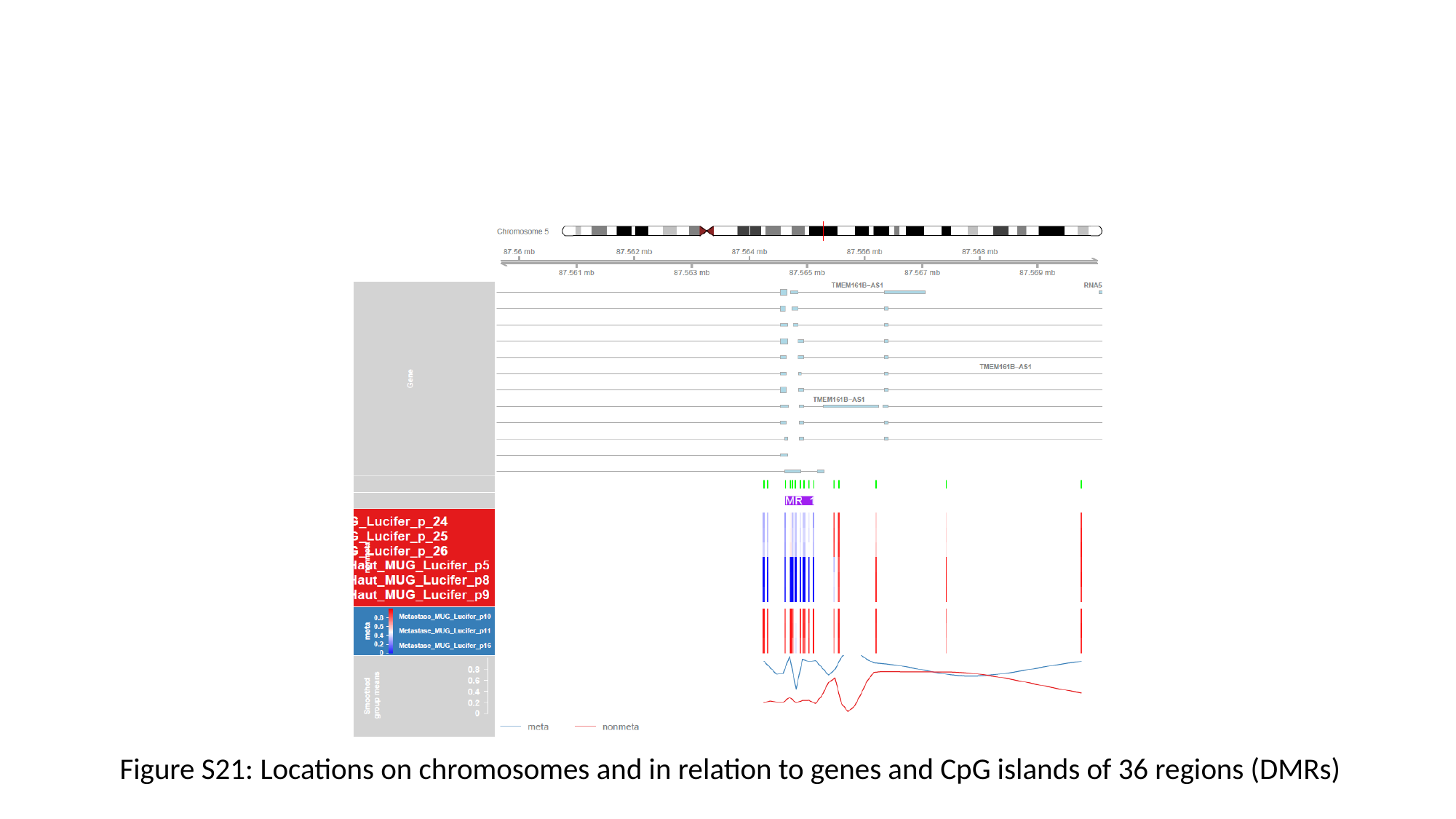

#
Figure S21: Locations on chromosomes and in relation to genes and CpG islands of 36 regions (DMRs)

## Slide 22
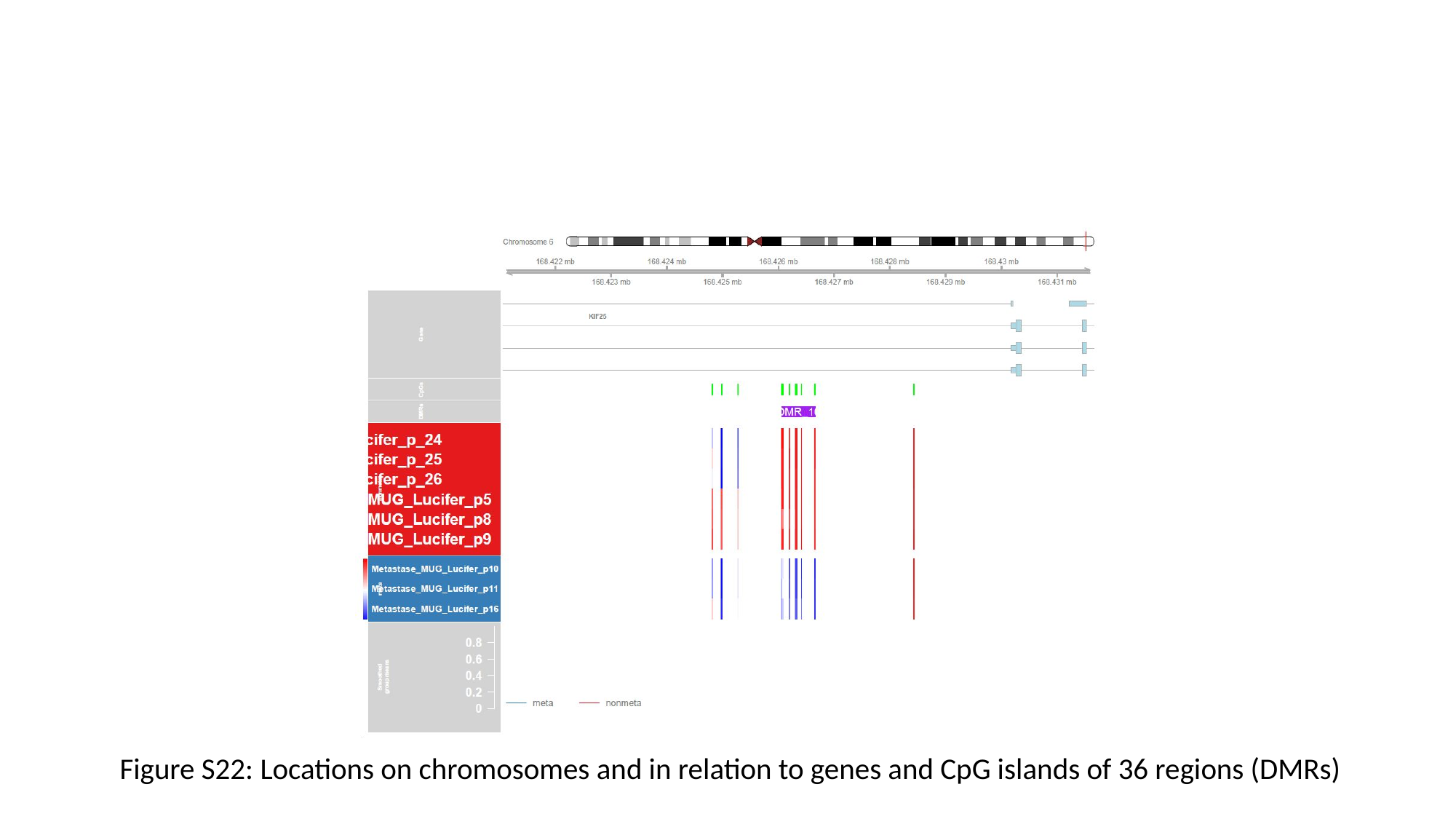

#
Figure S22: Locations on chromosomes and in relation to genes and CpG islands of 36 regions (DMRs)

## Slide 23
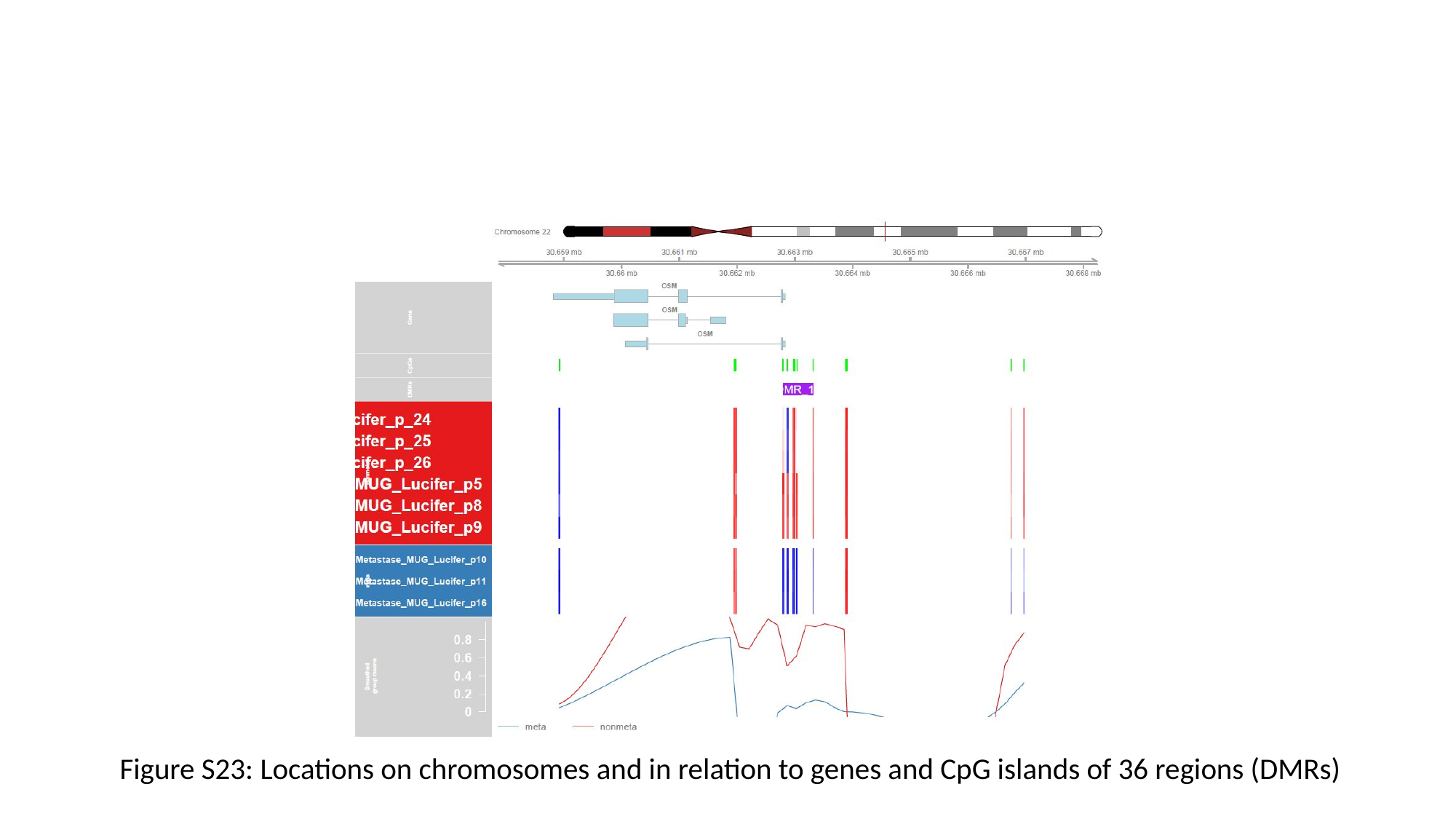

#
Figure S23: Locations on chromosomes and in relation to genes and CpG islands of 36 regions (DMRs)

## Slide 24
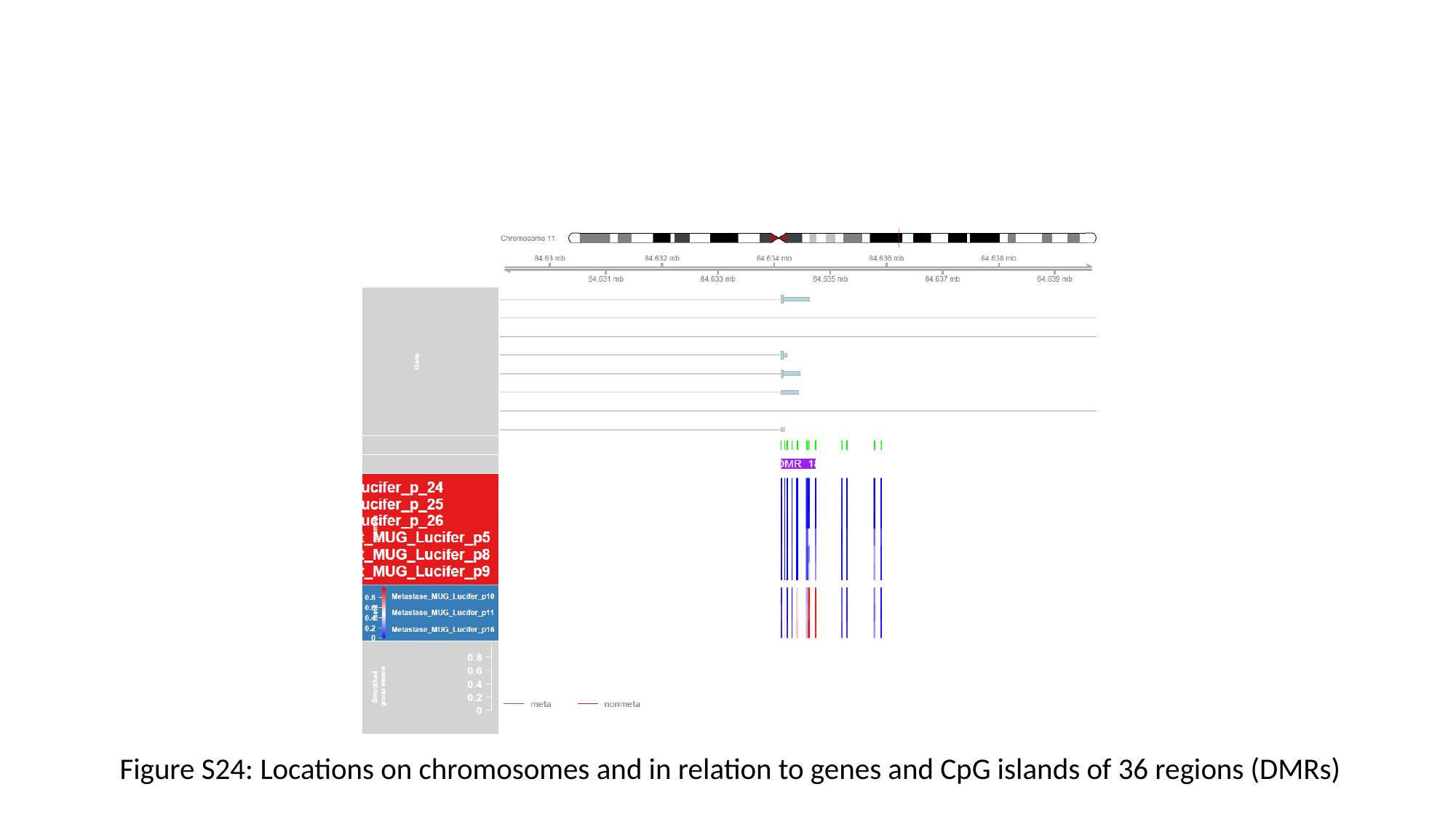

#
Figure S24: Locations on chromosomes and in relation to genes and CpG islands of 36 regions (DMRs)

## Slide 25
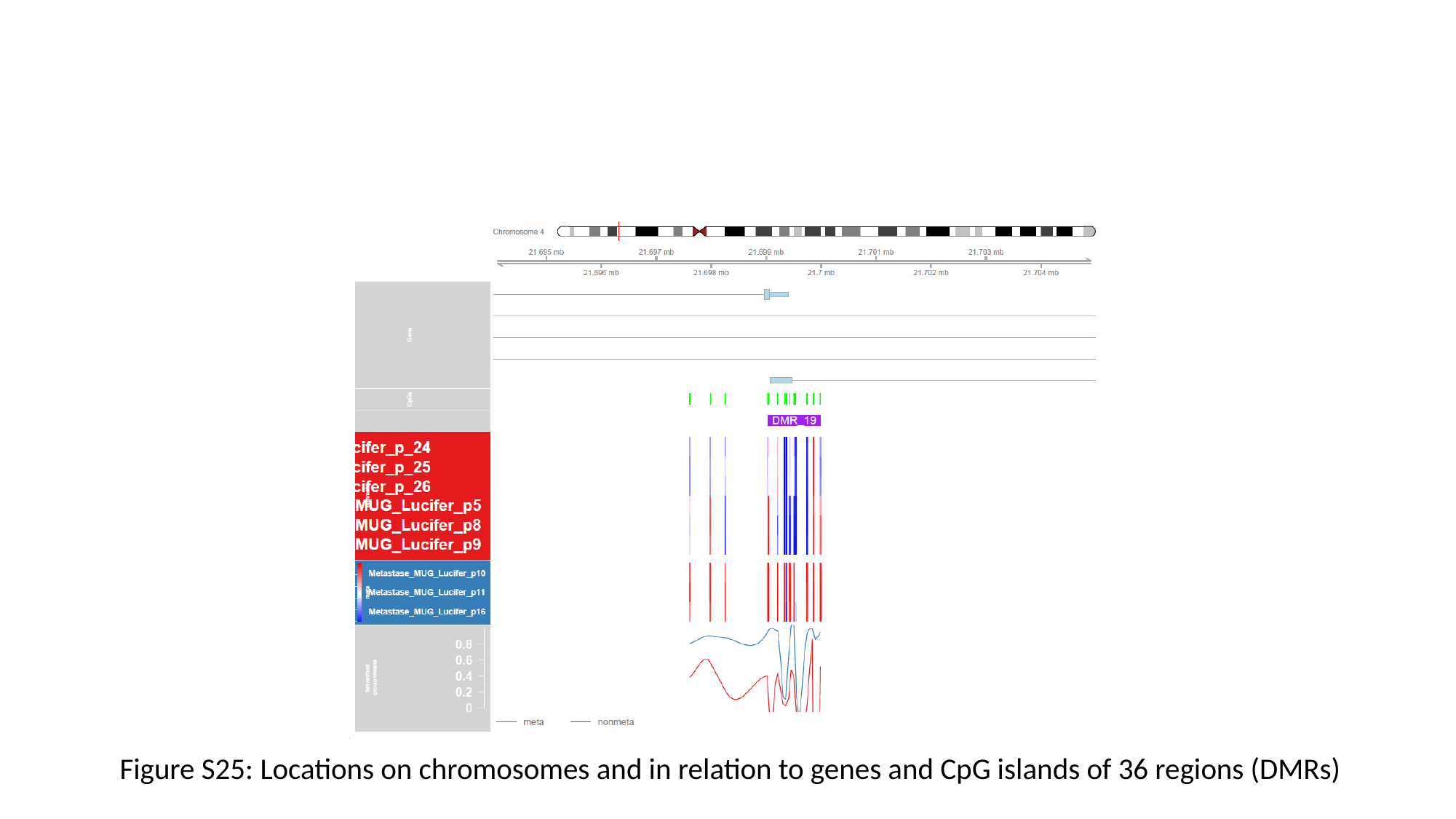

#
Figure S25: Locations on chromosomes and in relation to genes and CpG islands of 36 regions (DMRs)

## Slide 26
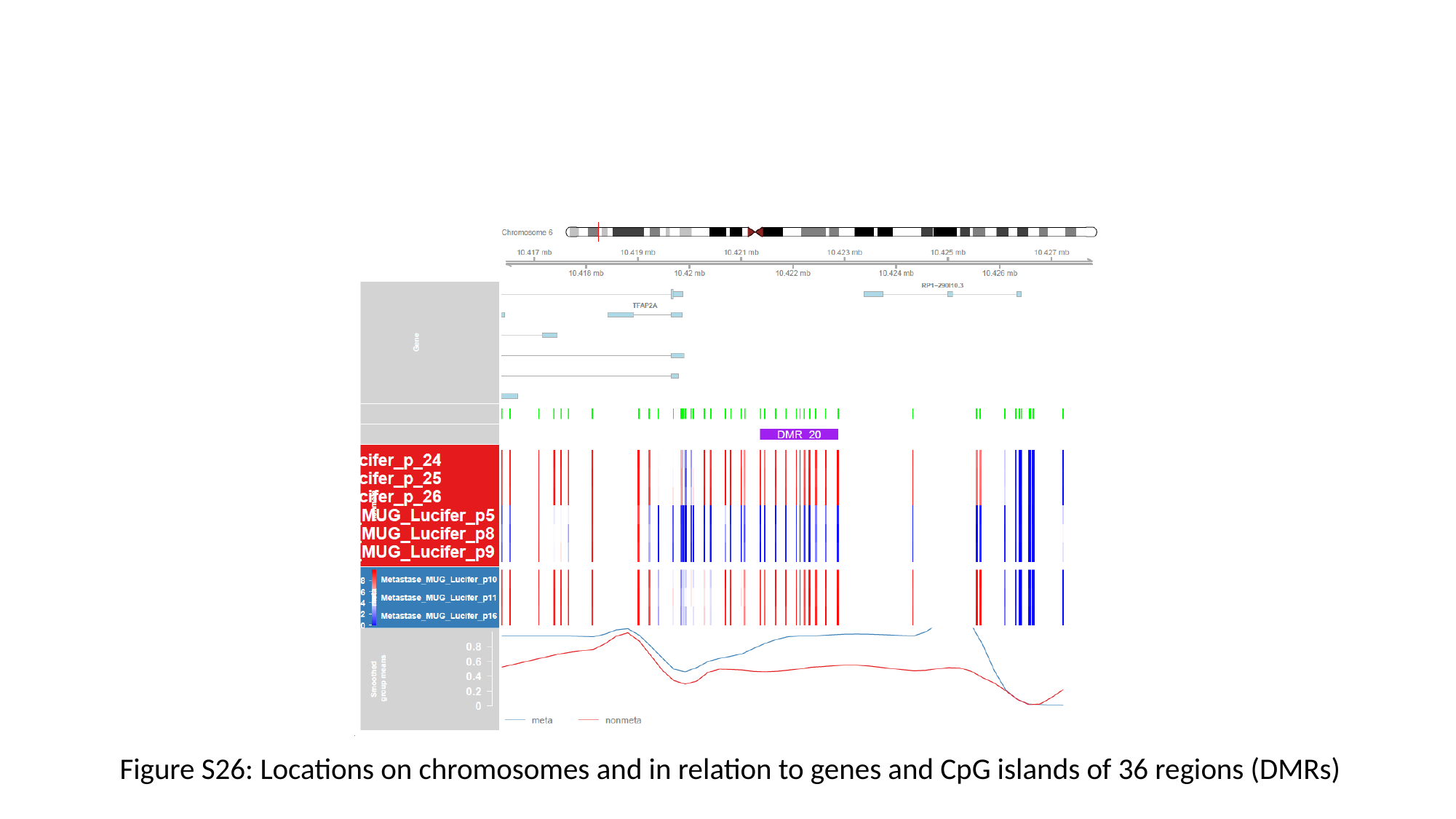

#
Figure S26: Locations on chromosomes and in relation to genes and CpG islands of 36 regions (DMRs)

## Slide 27
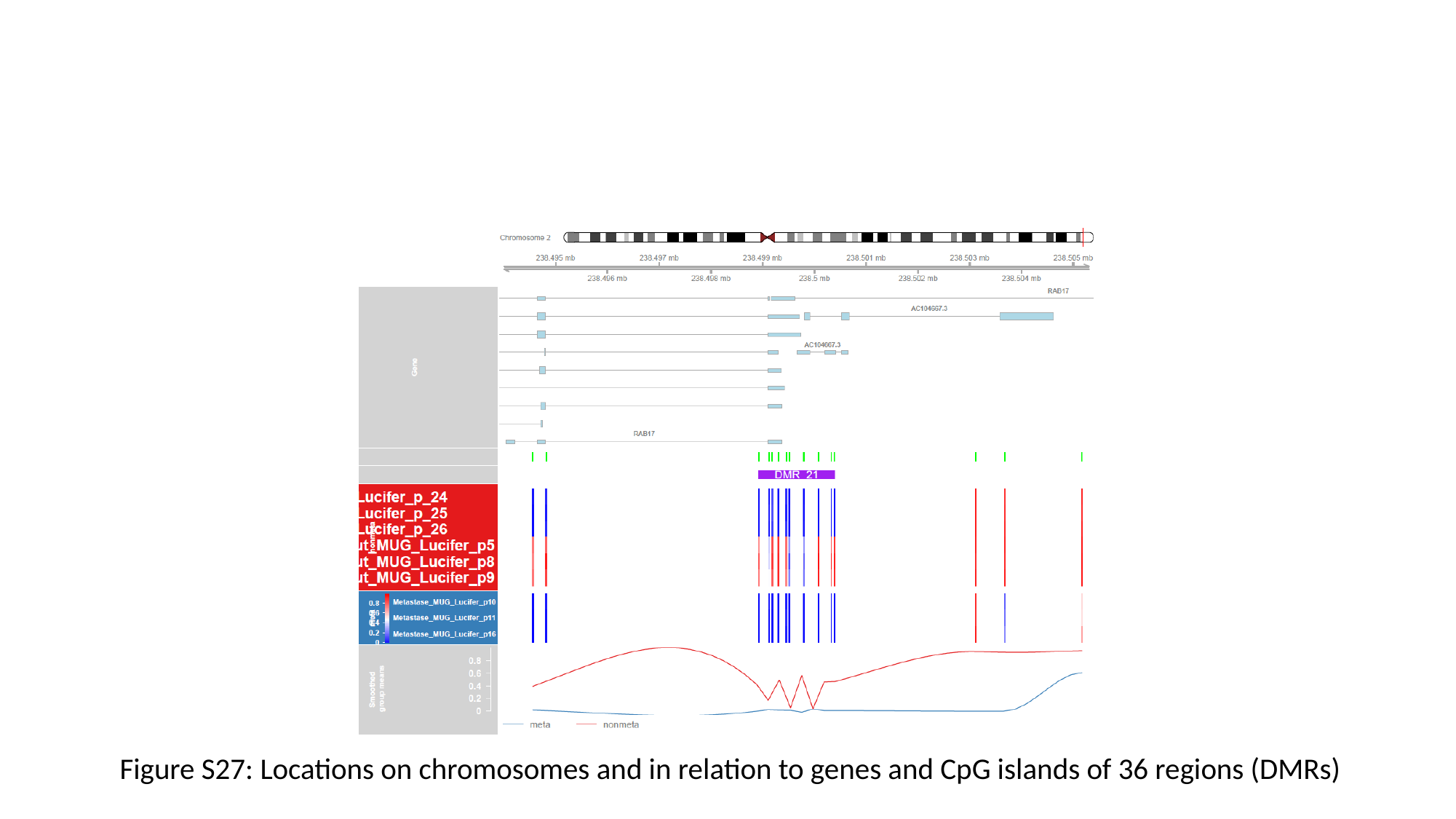

#
Figure S27: Locations on chromosomes and in relation to genes and CpG islands of 36 regions (DMRs)

## Slide 28
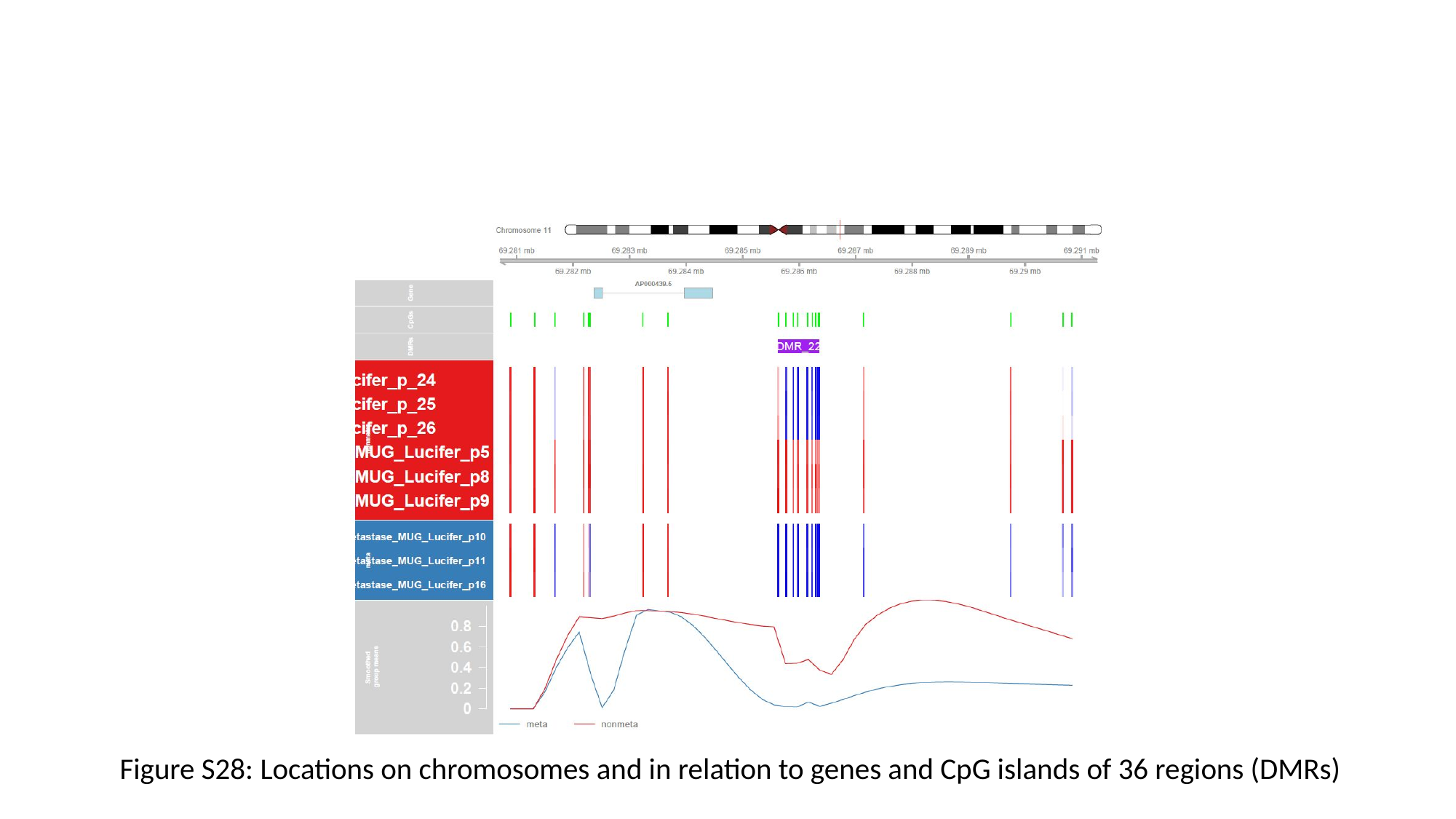

#
Figure S28: Locations on chromosomes and in relation to genes and CpG islands of 36 regions (DMRs)

## Slide 29
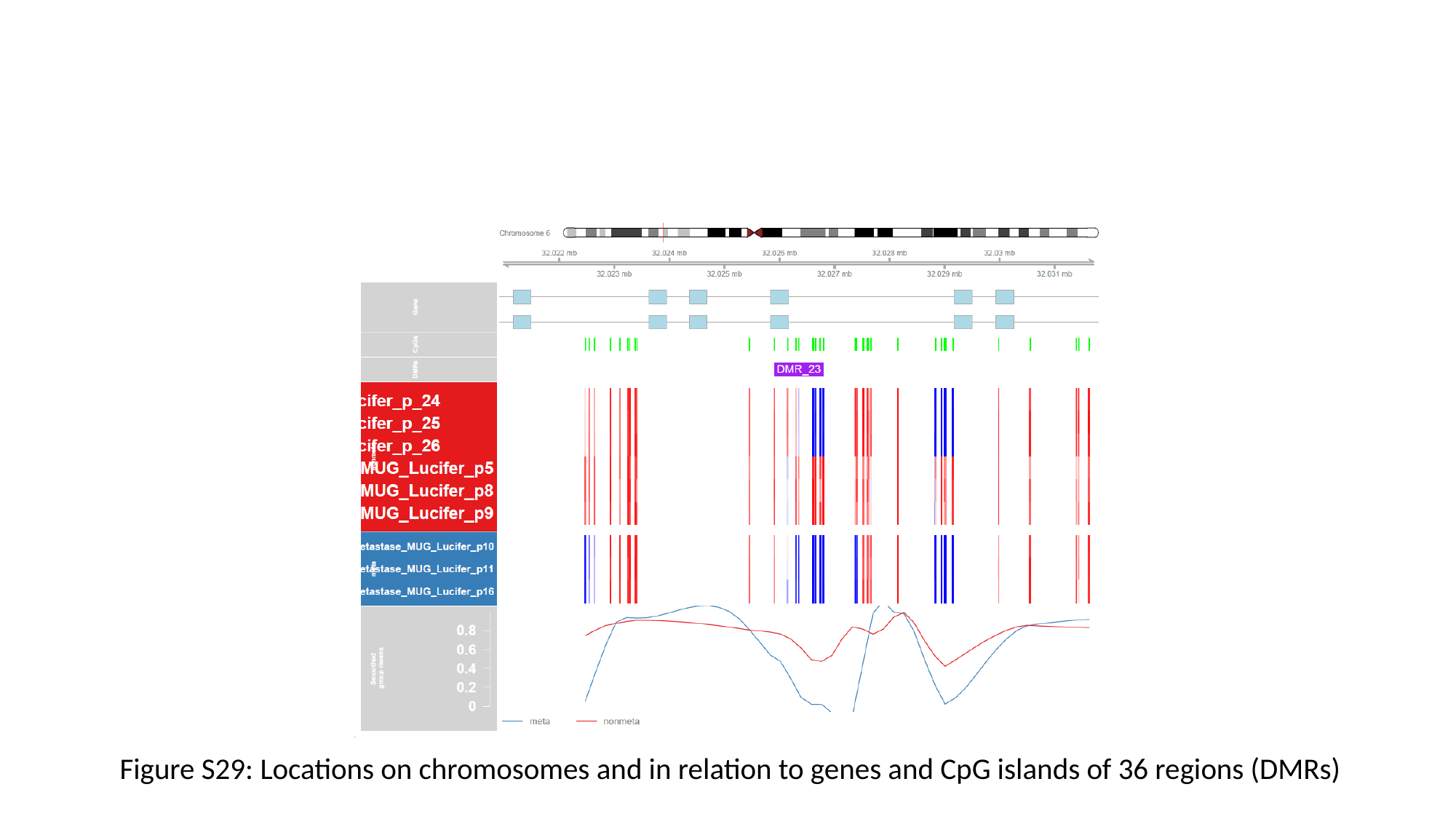

#
Figure S29: Locations on chromosomes and in relation to genes and CpG islands of 36 regions (DMRs)

## Slide 30
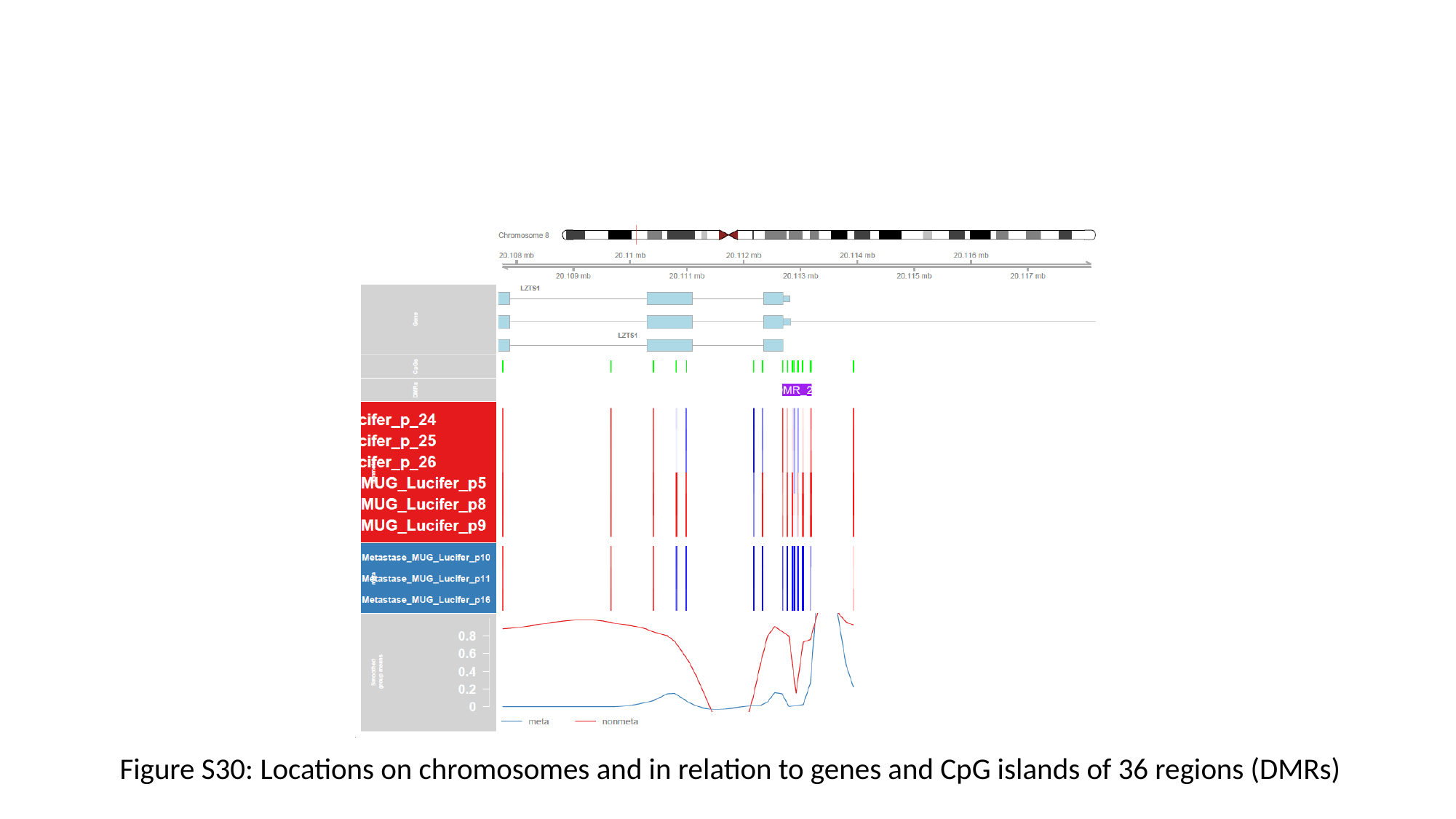

#
Figure S30: Locations on chromosomes and in relation to genes and CpG islands of 36 regions (DMRs)

## Slide 31
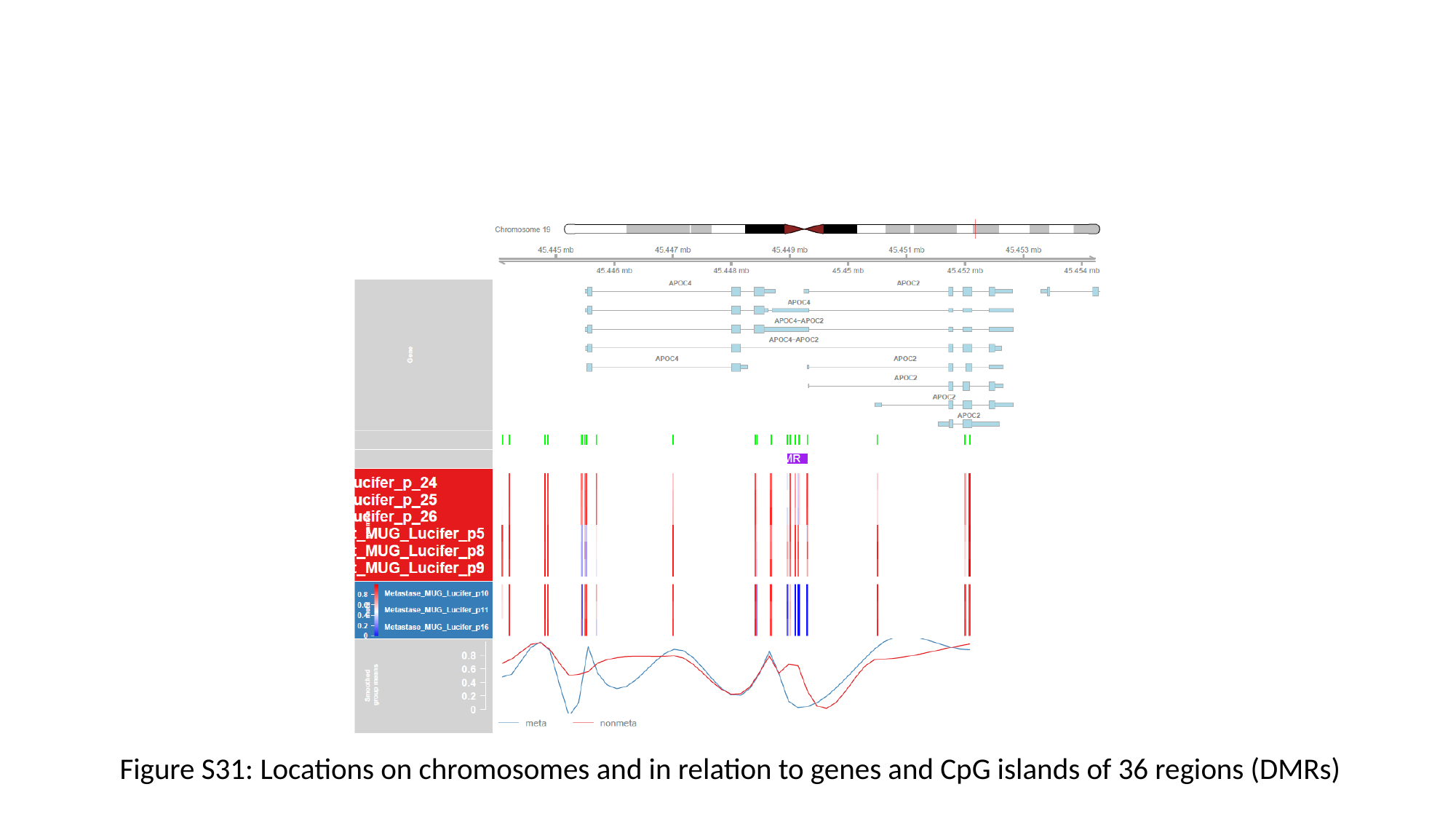

#
Figure S31: Locations on chromosomes and in relation to genes and CpG islands of 36 regions (DMRs)

## Slide 32
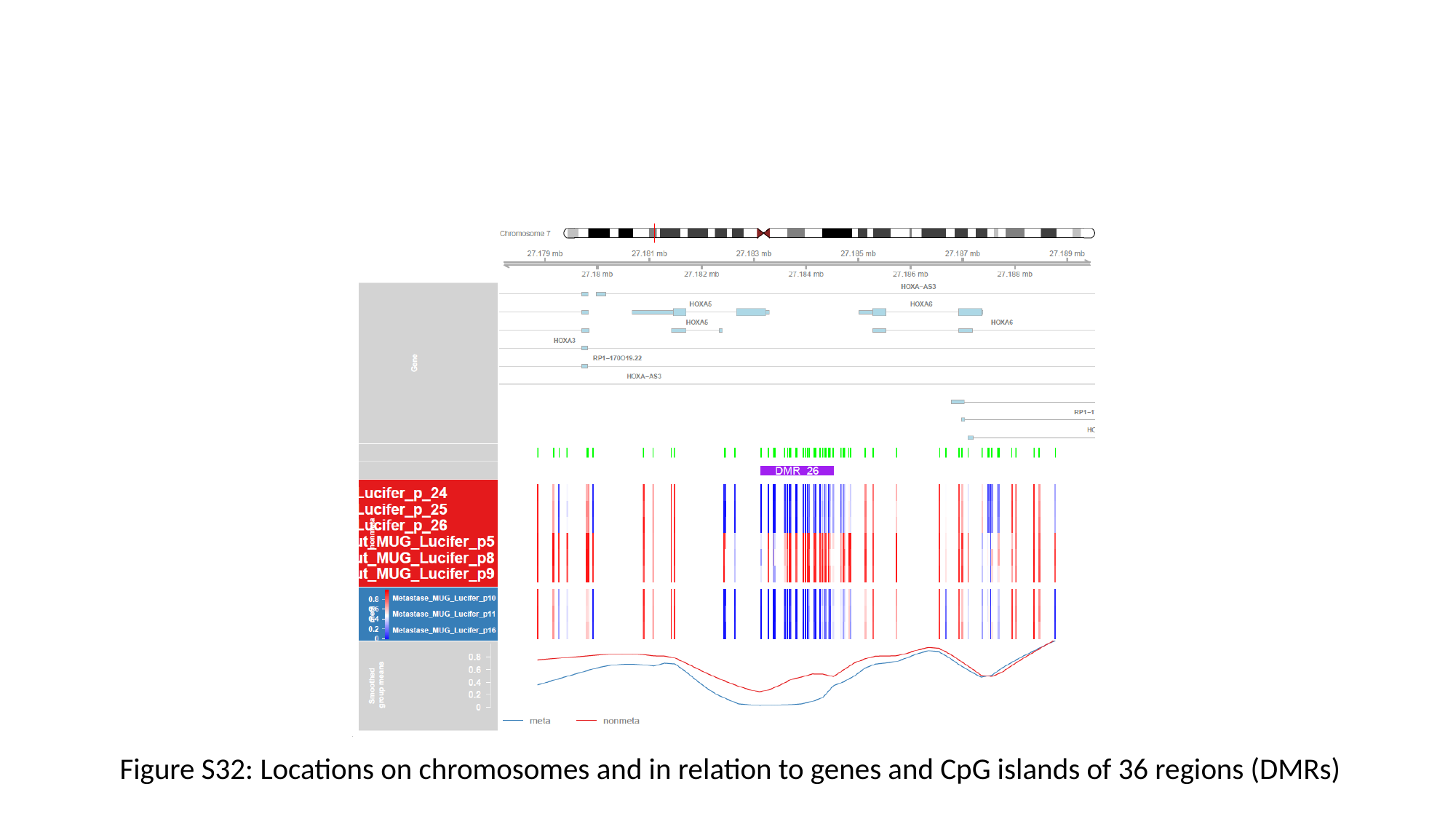

#
Figure S32: Locations on chromosomes and in relation to genes and CpG islands of 36 regions (DMRs)

## Slide 33
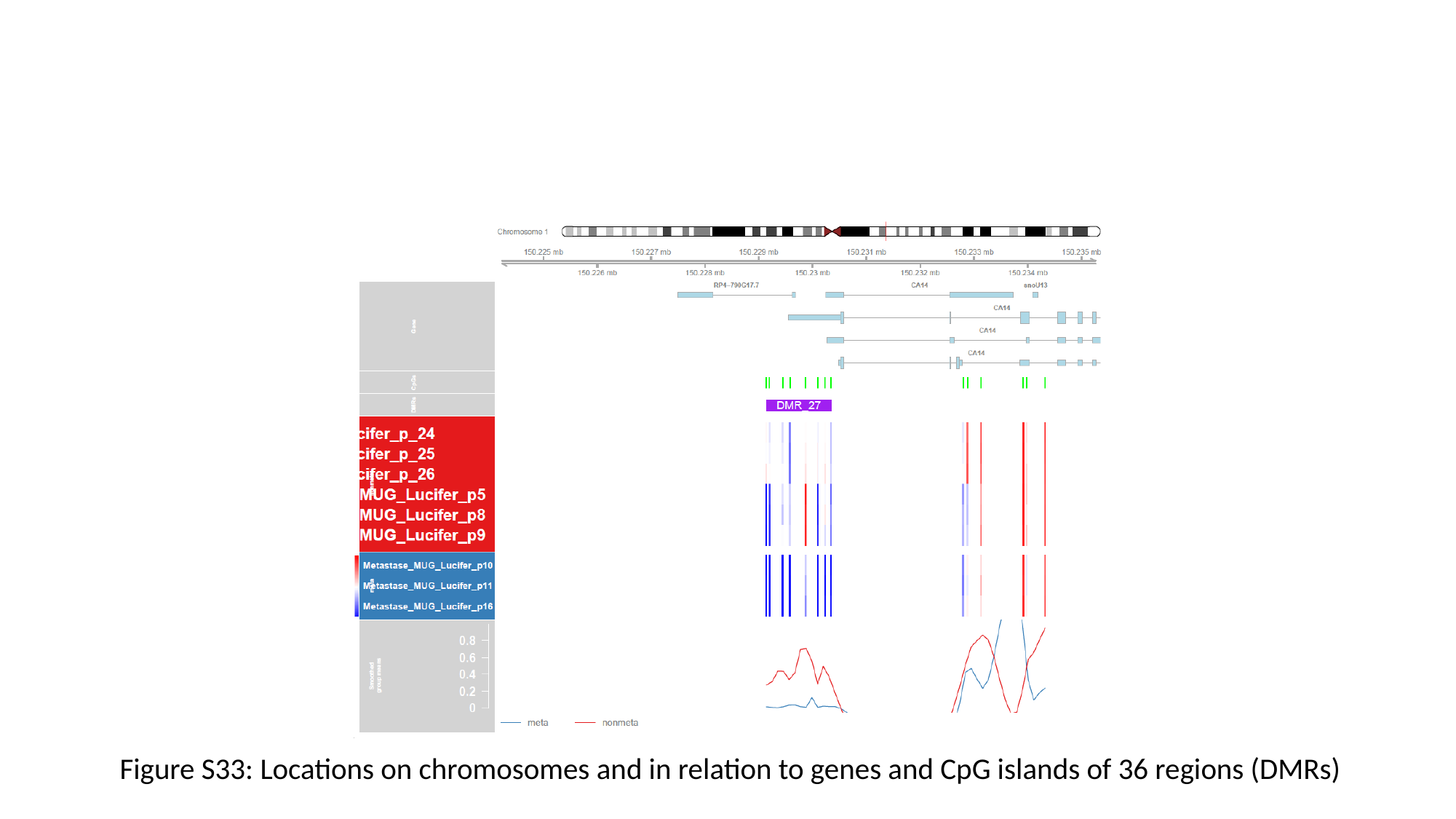

#
Figure S33: Locations on chromosomes and in relation to genes and CpG islands of 36 regions (DMRs)

## Slide 34
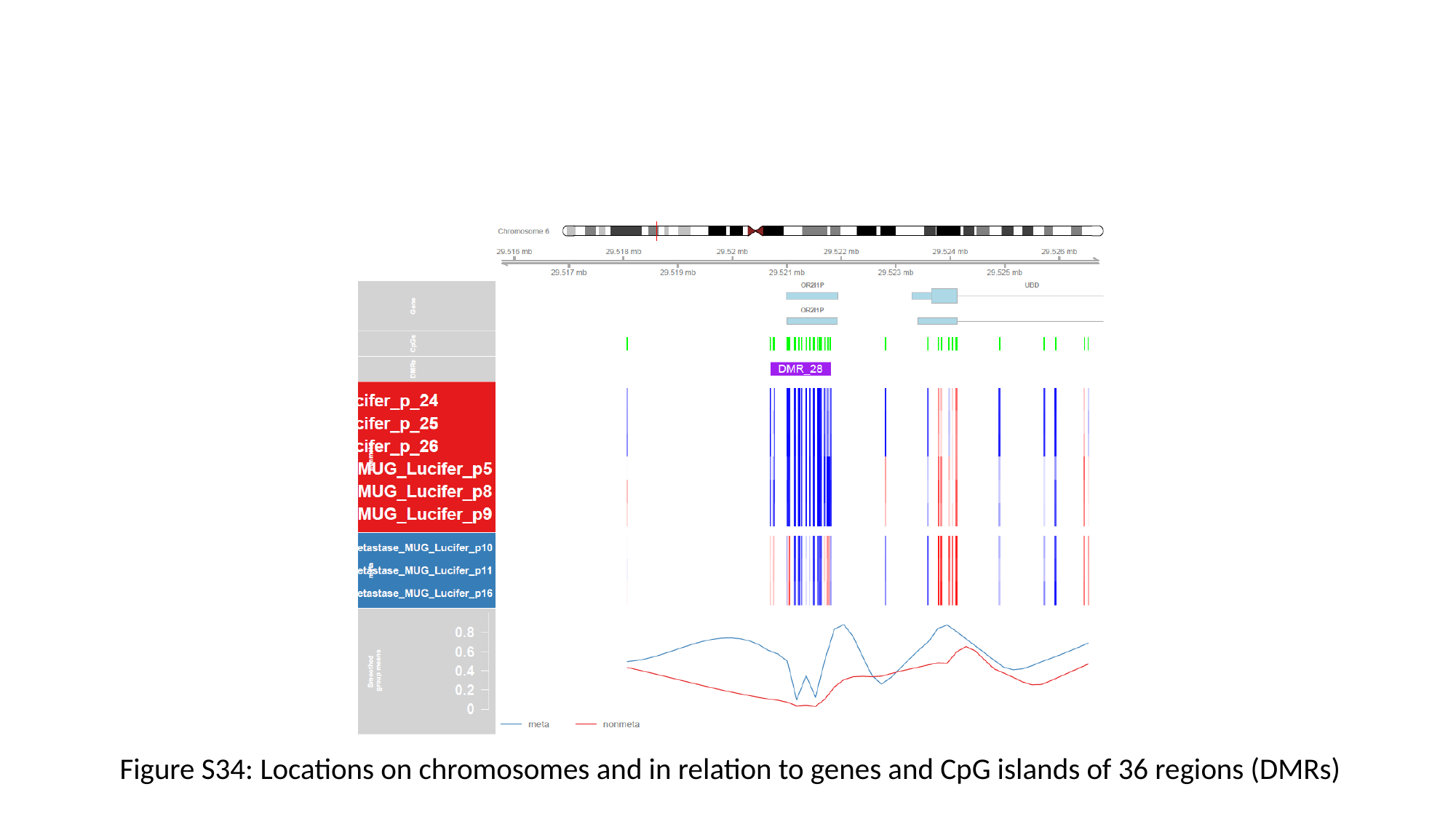

#
Figure S34: Locations on chromosomes and in relation to genes and CpG islands of 36 regions (DMRs)

## Slide 35
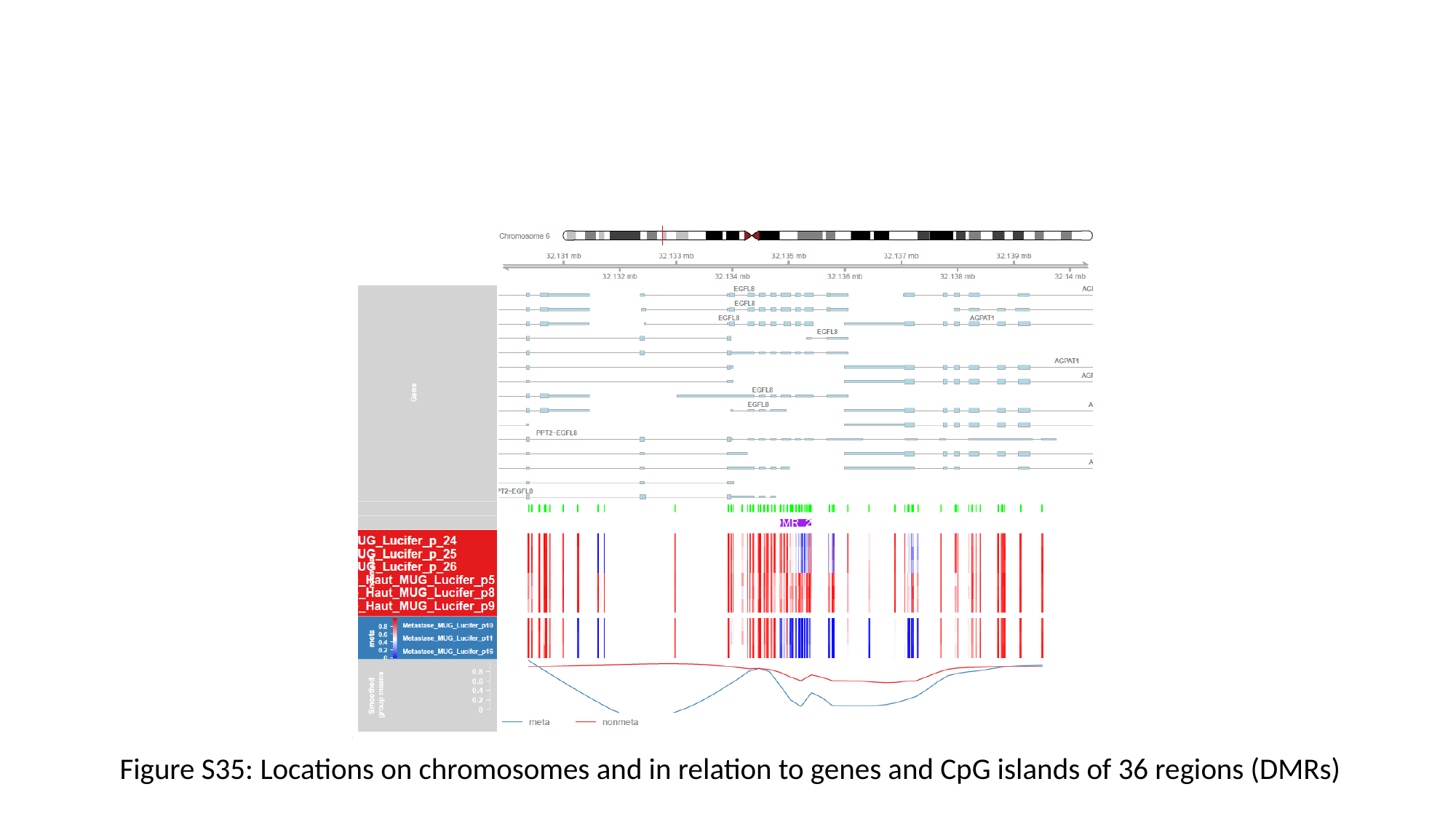

#
Figure S35: Locations on chromosomes and in relation to genes and CpG islands of 36 regions (DMRs)

## Slide 36
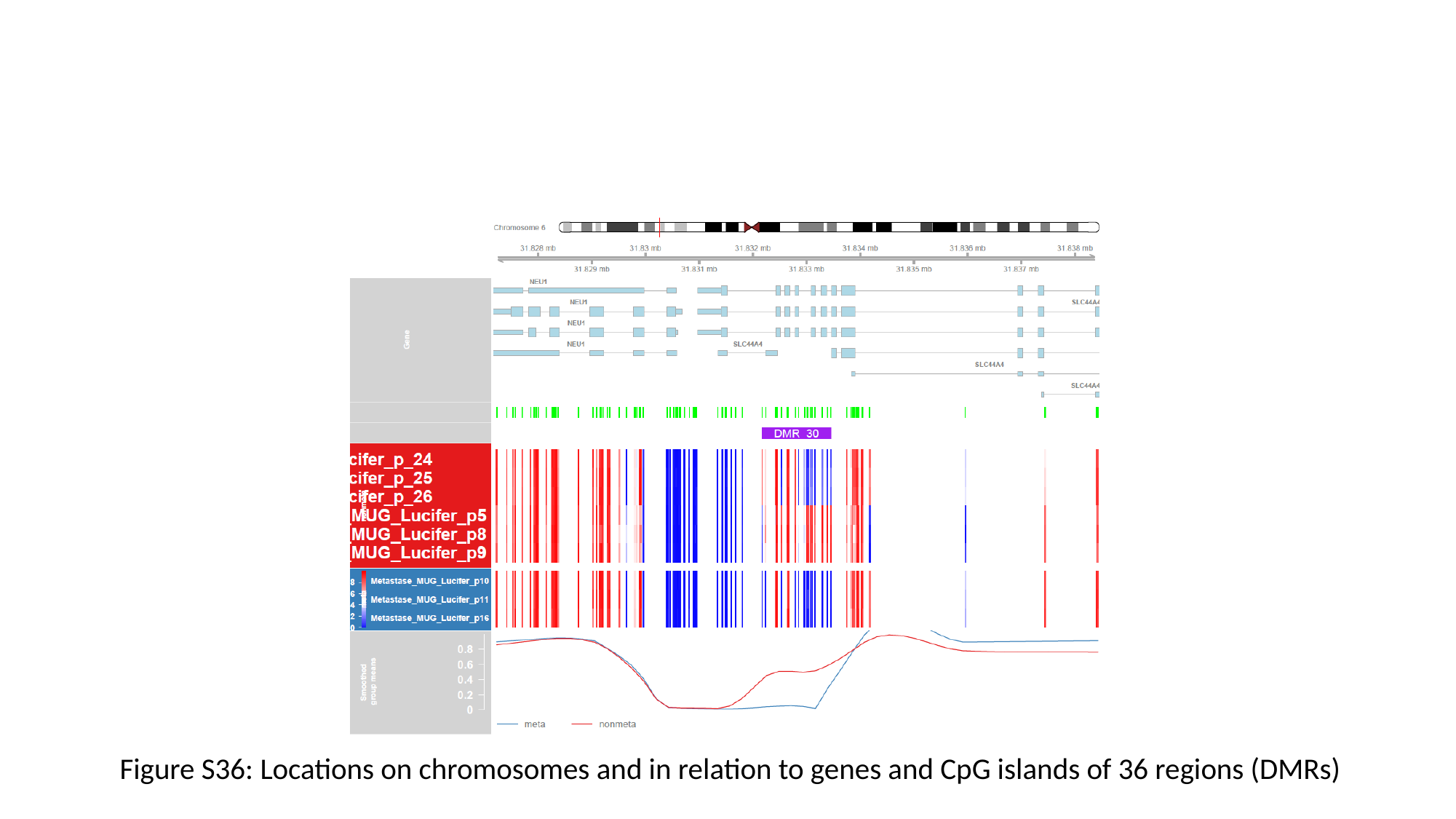

#
Figure S36: Locations on chromosomes and in relation to genes and CpG islands of 36 regions (DMRs)

## Slide 37
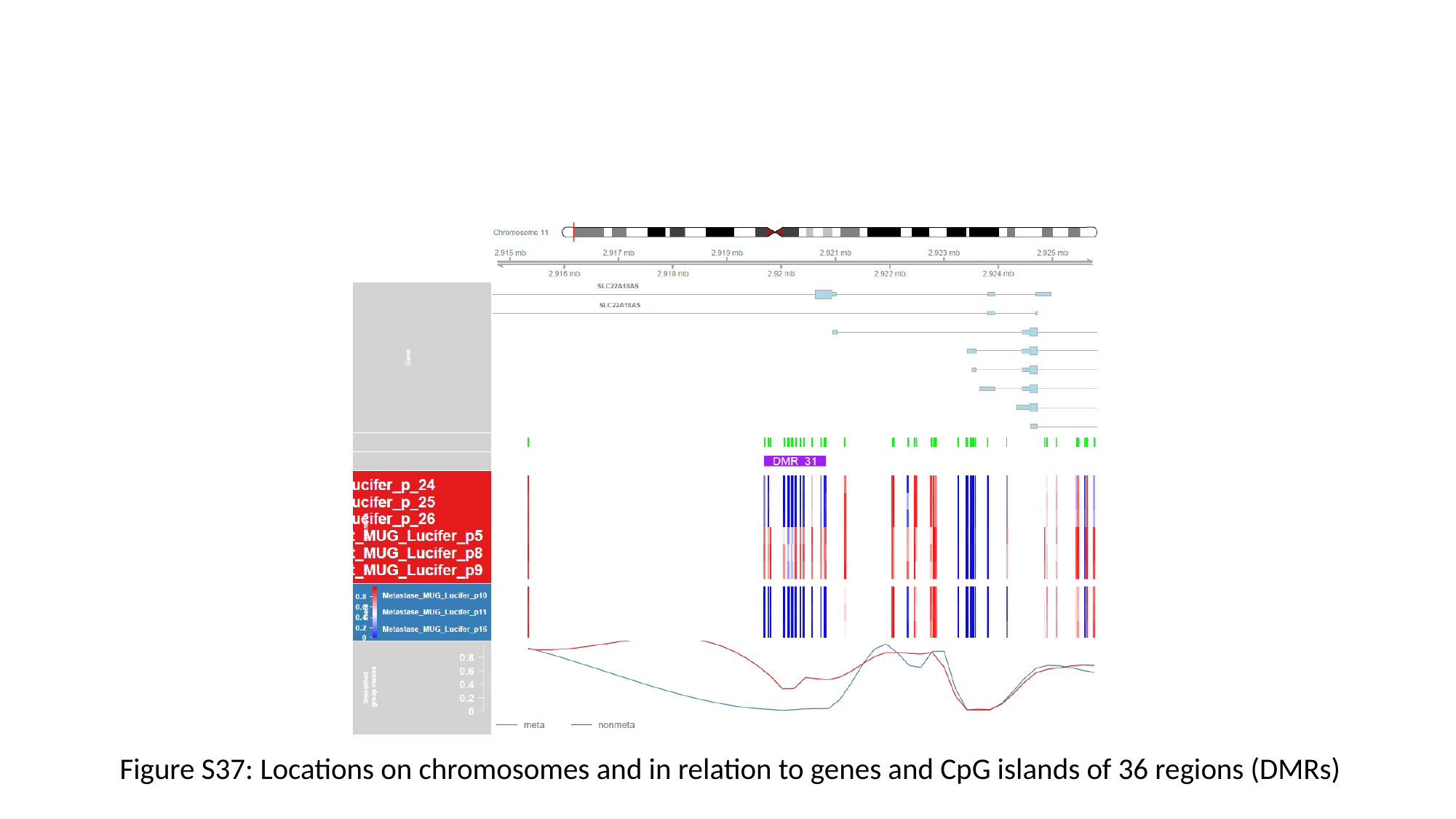

#
Figure S37: Locations on chromosomes and in relation to genes and CpG islands of 36 regions (DMRs)

## Slide 38
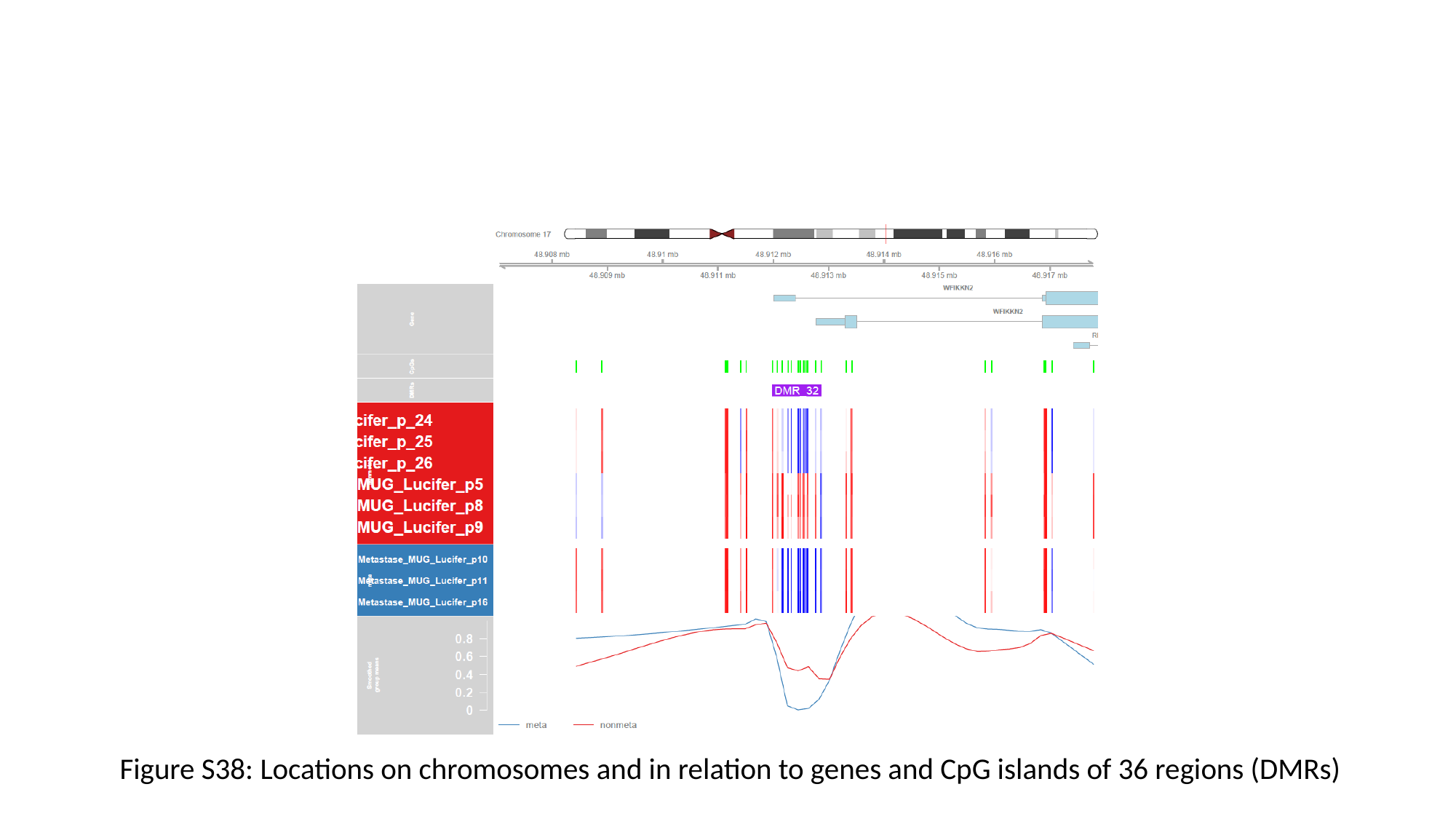

#
Figure S38: Locations on chromosomes and in relation to genes and CpG islands of 36 regions (DMRs)

## Slide 39
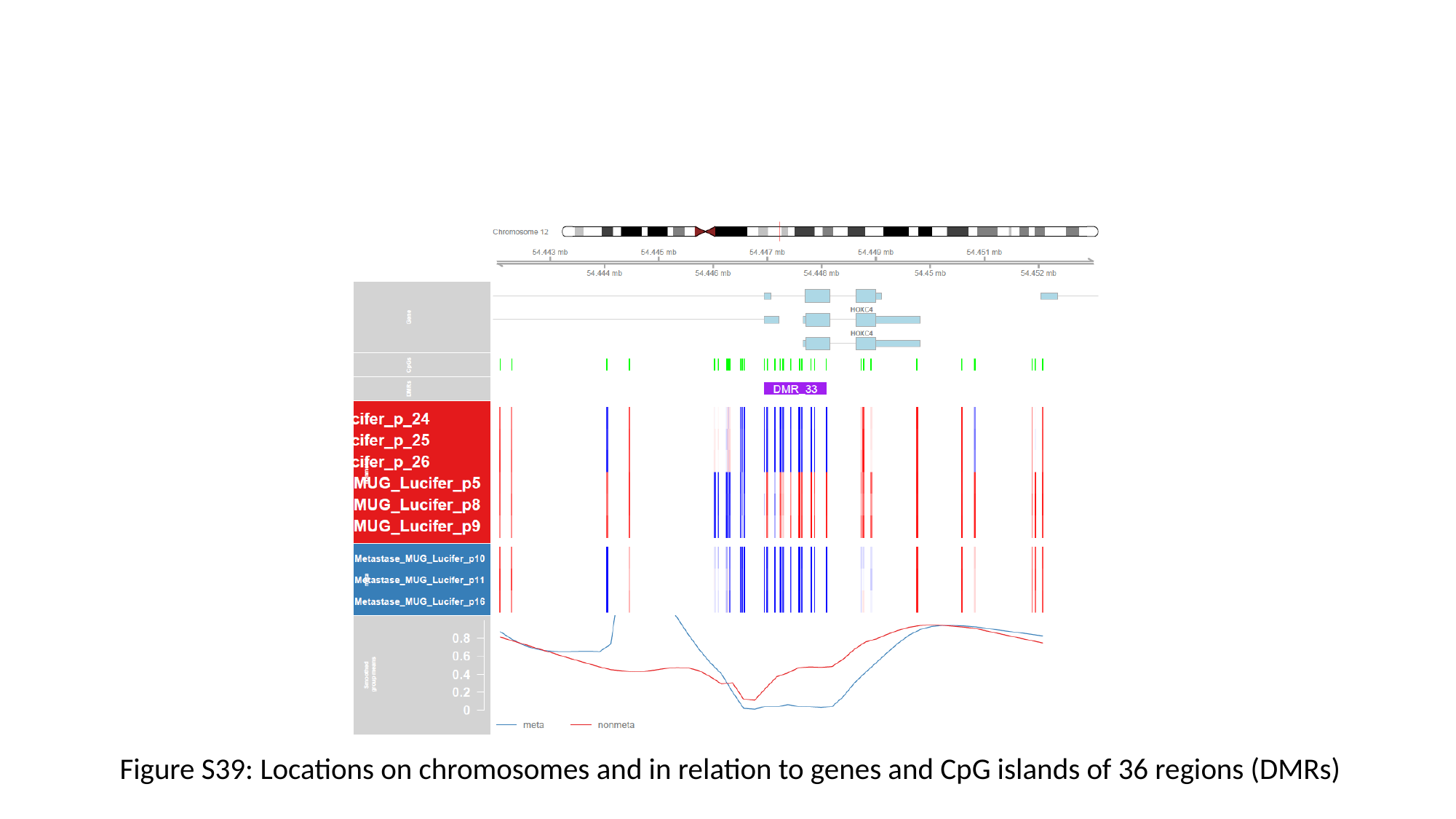

#
Figure S39: Locations on chromosomes and in relation to genes and CpG islands of 36 regions (DMRs)

## Slide 40
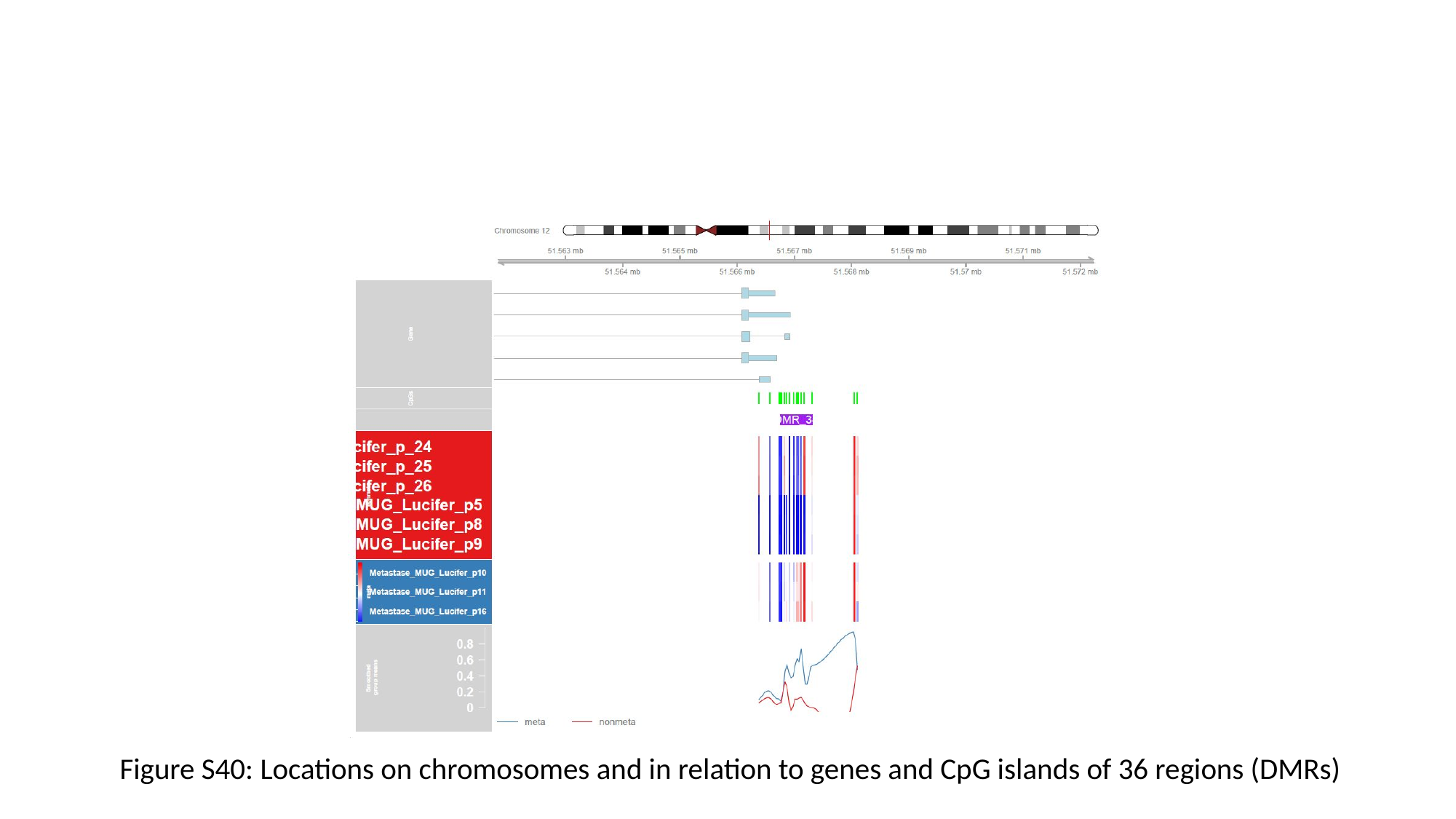

#
Figure S40: Locations on chromosomes and in relation to genes and CpG islands of 36 regions (DMRs)

## Slide 41
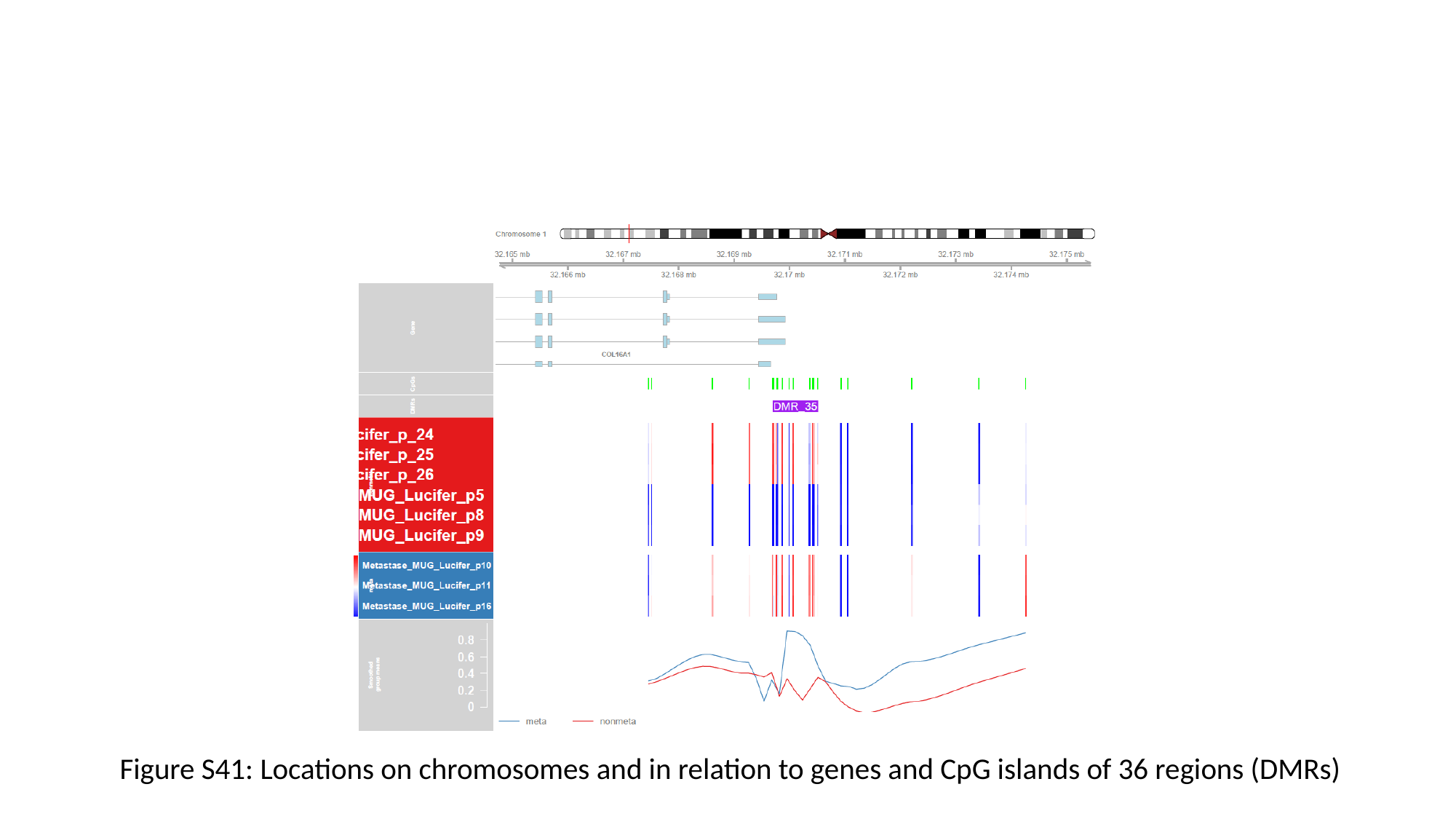

#
Figure S41: Locations on chromosomes and in relation to genes and CpG islands of 36 regions (DMRs)

## Slide 42
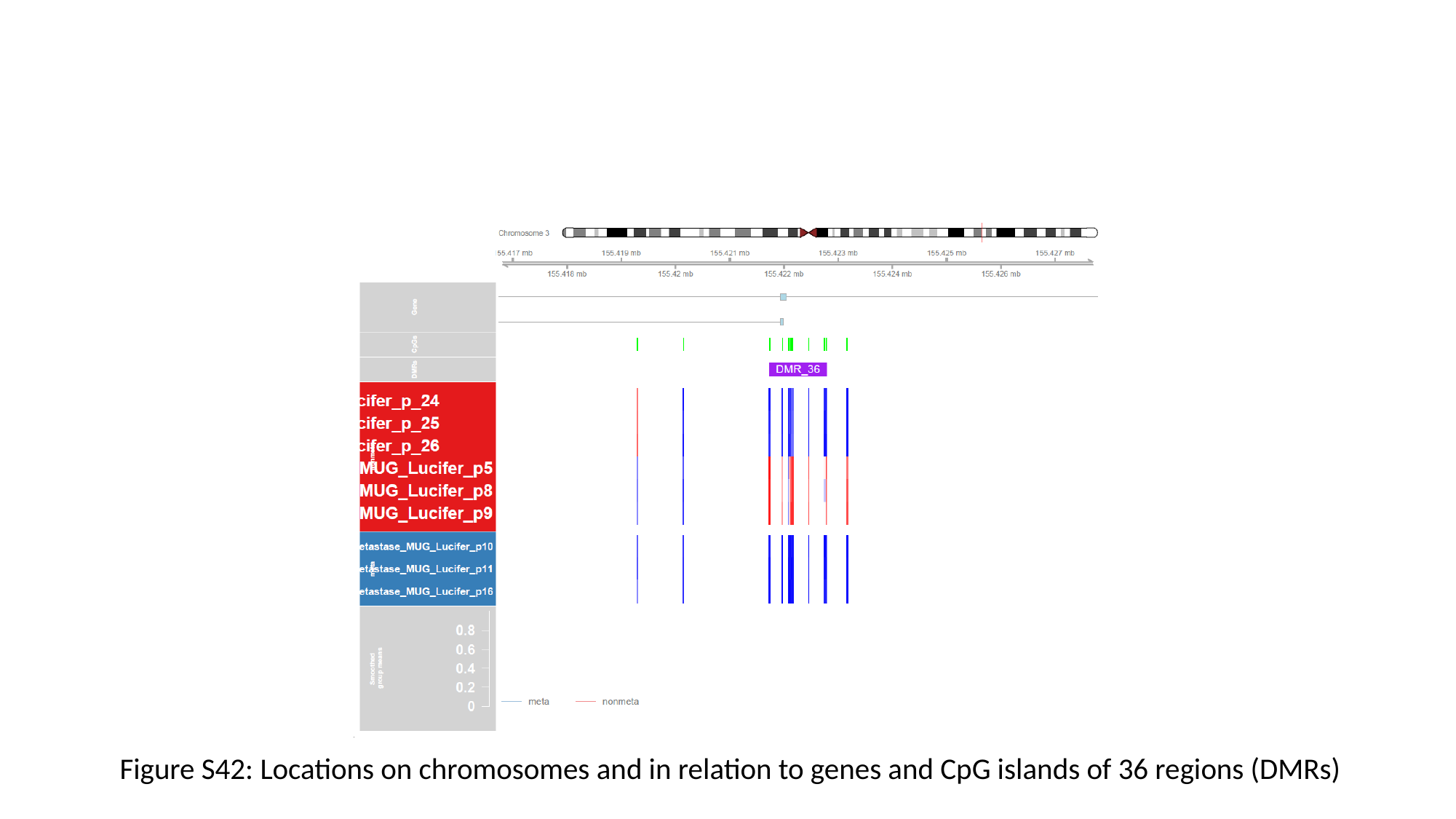

#
Figure S42: Locations on chromosomes and in relation to genes and CpG islands of 36 regions (DMRs)
