# Supplementary material for: Targeting epigenetic features in clear cell sarcomas based on patient-derived cell lines
Source: J Transl Med. 2023 Jan 29;21:54. doi: 10.1186/s12967-022-03843-4 (PMC9884415; doi:10.1186/s12967-022-03843-4)
Supplement: Supplementary file 2 — Additional file 2: Table S1. STR profiles of MUG Lucifer prim tumor tissue, tissue from the patient’s skin, MUG Lucifer prim passages p17 and p70 and MUG Lucifer met passages p5 and p62. Table S2. Copy number profiles of MUG Lucifer cell lines. Table S3. Number of DMPs with respect to CpG Island locations and gene annotations. [file 12967_2022_3843_MOESM2_ESM.docx]

**Table S1**: STR profiles of MUG Lucifer prim tumor tissue, tissue from the patient’s skin, MUG Lucifer prim passages p17 and p70 and MUG Lucifer met passages p5 and p62.

| STR-Locus | D3S1358 | TH01 | D21S11 | D18S51 | Penta E | D5S818 | D13S317 | D7S820 |
| --- | --- | --- | --- | --- | --- | --- | --- | --- |
| Tumor tissue | 16, 17 | 9, 9.3 | 30, 32.2 | 12, 19 | 13 | 11 | 13, 15 | 8, 11 |
| Skin tissue | 16, 17 | 9, 9.3 | 30, 32.2 | 12, 19 | 13 | 11 | 13, 15 | 8, 11 |
| MUG Lucifer prim, p17 | 16, 17 | 9, 9.3 | 30, 32.2 | 12, 19 | 13 | 11 | 13, 15 | 8, 11 |
| MUG Lucifer met, p5 | 16, 17 | 9, 9.3 | 30, 32.2 | 12, 19 | 13 | 11 | 13, 15 | 8, 11 |
| MUG Lucifer prim, p70 | 16, 17 | 9, 9.3 | 30, 32.2 | 12, 19 | 13 | 11 | 13, 15 | 8, 11 |
| MUG Lucifer met, p62 | 16, 17 | 9, 9.3 | 30, 32.2 | 12, 19 | 13 | 11 | 13, 15 | 8, 11 |
| STR-Locus | D16S539 | CSF1PO | Penta D | Amelogenin | vWA | D8S1179 | TPOX | FGA |
| Tumor tissue | 9, 14 | 10, 11 | 9 | X | 17, 19 | 12, 14 | 8, 12 | 21, 25 |
| Skin tissue | 9, 14 | 10, 11 | 9 | X | 17, 19 | 12, 14 | 8, 12 | 21, 25 |
| MUG Lucifer prim, p17 | 9, 14 | 10, 11 | 9 | X | 17, 19 | 12, 14 | 8, 12 | 21, 25 |
| MUG Lucifer met, p5 | 9, 14 | 10, 11 | 9 | X | 17, 19 | 12, 14 | 8, 12 | 21, 25 |
| MUG Lucifer prim, p70 | 9, 14 | 11 | 9 | X | 17, 19 | 12, 14 | 8, 12 | 21, 25 |
| MUG Lucifer met, p62 | 9, 14 | 11 | 9 | X | 17, 19 | 12, 14 | 8, 12 | 21, 25 |

**Table S2:** Copy number profiles of MUG Lucifer cell lines.

|  |  |  | **Start** | **Stop** | **Size [MB]** | **log2 ratio** | **Call** | **Genes** |
| --- | --- | --- | --- | --- | --- | --- | --- | --- |
| Chromosome 1 | | PT | 150000001 | 248000000 | 98 | -0.015 | Balanced |  |
|  |  | p17 | 150000001 | 248000000 | 98 | 0.002 | Balanced |  |
|  |  | MET | 150000001 | 248000000 | 98 | 0.437 | Gain | ABL2, ARNT, DDR2, FCGR2B, FCRL4, LMNA, MLLT11, MUC1, NTRK1, PBX1, PRCC, PRRX1, S100A7, SDHC, SETDB1, TPM3 |
|  |  | p5 | 150000001 | 248000000 | 98 | 0.594 | Gain | ABL2, ARNT, DDR2, FCGR2B, FCRL4, LMNA, MLLT11, MUC1, NTRK1, PBX1, PRCC, PRRX1, S100A7, SDHC, SETDB1, TPM3 |
| Chromosome 5 | | PT | 1000001 | 11000000 | 10 | 0.497 | Gain |  |
|  |  | PT | 11000000 | 180000000 | 169 | 0.383 | Gain | ACSL6, AFF4, APC, ARHGAP26, CD74, CSF1R, EBF1, FGFR4, IL6ST, ITK, MAP3K1, NPM1, NSD1, PDGFRB, PIK3R1, PWWP2A, RAD17, TLX3, CDH10, DROSHA, IL7R, LIFR |
|  |  | p17 | 1000001 | 46000000 | 45 | 0.625 | HL-Amp |  |
|  |  | p17 | 50000001 | 180000000 | 130 | 0.231 | Gain | ACSL6, AFF4, APC, ARHGAP26, CD74, CSF1R, EBF1, FGFR4, IL6ST, ITK, MAP3K1, NPM1, NSD1, PDGFRB, PIK3R1, PWWP2A, RAD17, TLX3 |
|  |  | MET | 1000001 | 46000000 | 45 | 0.428 | Gain |  |
|  |  | MET | 50000001 | 180000000 | 130 | 0.003 | Balanced |  |
|  |  | p5 | 1000001 | 46000000 | 45 | 0.560 | Gain |  |
|  |  | p5 | 50000001 | 180000000 | 130 | 0.010 | Balanced |  |
| Chromosome 8 | | PT | 1000001 | 125000000 | 124 | 0.824 | Gain |  |
|  |  | PT | 125000001 | 145000000 | 20 | 1.180 | HL-Amp | FAM135B, MYC, NDRG1, RECQL4 |
|  |  | p17 | 1000001 | 145000000 | 144 | 0.968 | HL-Amp | only one segment called |
|  |  | MET | 1000001 | 125000000 | 124 | 0.774 | Gain |  |
|  |  | MET | 125000001 | 145000000 | 20 | 1.034 | HL-Amp | FAM135B, MYC, NDRG1, RECQL4 |
|  |  | p5 | 1000001 | 125000000 | 124 | 0.980 | Gain |  |
|  |  | p5 | 125000001 | 145000000 | 20 | 1.301 | HL-Amp | FAM135B, MYC, NDRG1, RECQL4 |
| Chromosome 9 | | PT | 20000001 | 30000000 | 10 | -0.719 | Deletion | CDKN2A, FANCG, MLLT3, PAX5 |
|  |  | p17 | 19000001 | 30000000 | 11 | -1.074 | Deletion | CDKN2A, FANCG, MLLT3, PAX5 |
|  |  | MET | 21000001 | 30000000 | 9 | -1.769 | Deletion | CDKN2A, FANCG, PAX5 |
|  |  | p5 | 21000001 | 30000000 | 9 | ?? | Deletion | CDKN2A, FANCG, PAX5 ?? Segment called, but not visible in plot |
| Chromosome 15 | | PT | 25000001 | 102000000 | 77 | -0.013 | Balanced |  |
|  |  | p17 | 38000001 | 49000000 | 11 | -0.977 | Deletion | B2M, BUB1B, HMGN2P46, KNL1, KNSTRN |
|  |  | MET | 25000001 | 60000000 | 35 | -0.669 | Deletion | B2M, BUB1B, C15orf65, HMGN2P46, KNL1, KNSTRN, MYO5A, NUTM1, TCF12, USP8 |
|  |  | p5 | 25000001 | 60000000 | 35 | -0.968 | Deletion | B2M, BUB1B, C15orf65, HMGN2P46, KNL1, KNSTRN, MYO5A, NUTM1, TCF12, USP8 |
| Chromosome 16 | | PT | 73000001 | 90000000 | 17 | -0.643 | Deletion | FUS, IL21R, PALB2, PRKCB |
|  |  | p17 | 1000001 | 90000000 | 89 | -0.056 | Balanced |  |
|  |  | MET | 1000001 | 90000000 | 89 | -0.021 | Balanced |  |
|  |  | p5 | 1000001 | 90000000 | 89 | -0.028 | Balanced |  |
| Chromosome 17 | | PT | 1000001 | 81000000 | 80 | 0.449 | Gain |  |
|  |  | p17 | 1000001 | 81000000 | 80 | 0.619 | Gain |  |
|  |  | MET | 1000001 | 53000000 | 52 | 0.427 | Gain |  |
|  |  | MET | 53000001 | 81000000 | 28 | 0.762 | HL-Amp | AXIN2, BRIP1, CANT1, CD79B, CLTC, DDX5, H3F3B, HLF, MSI2, PPM1D, PRKAR1A, RNF213, RNF43, SEPT9, SRSF2 |
|  |  | p5 | 1000001 | 53000000 | 52 | 0.575 | Gain |  |
|  |  | p5 | 53000001 | 81000000 | 28 | 0.998 | HL-Amp | AXIN2, BRIP1, CANT1, CD79B, CLTC, DDX5, H3F3B, HLF, MSI2, PPM1D, PRKAR1A, RNF213, RNF43, SEPT9, SRSF2 |

**Table S3**: Number of DMPs with respect to CpG Island locations and gene annotations.

|  | **prim vs. met**  **_down** | **prim vs. met**  **_up** | **prim vs. NHDF**  **_down** | **prim vs. NHDF**  **_up** | **NHDF vs. met**  **_down** | **NHDF vs. met**  **_up** |
| --- | --- | --- | --- | --- | --- | --- |
| **island** | 8 | 5 | 81 | 153 | 90 | 217 |
| **opensea** | 341 | 212 | 2899 | 947 | 2352 | 997 |
| **shelf** | 9 | 17 | 132 | 81 | 189 | 73 |
| **shore** | 20 | 53 | 254 | 272 | 324 | 291 |
| **TSS1500** | 24 | 18 | 210 | 149 | 184 | 154 |
| **TSS200** | 4 | 4 | 70 | 43 | 73 | 57 |
| **5'UTR** | 13 | 25 | 229 | 147 | 216 | 133 |
| **1stExon** | 9 | 1 | 23 | 26 | 20 | 33 |
| **Body** | 124 | 165 | 1350 | 657 | 1407 | 645 |
| **ExonBnd** | 3 | 3 | 19 | 1 | 25 | 4 |
| **3'UTR** | 6 | 11 | 66 | 20 | 53 | 21 |
| **IGR** | 195 | 60 | 1399 | 410 | 977 | 531 |
